# Synthesis of chiral cyclohexanes and carbasugars by 6-*exo*-*dig* radical cyclisation reactions

**DOI:** 10.3762/bjoc.4.43

**Published:** 2008-11-19

**Authors:** Rajeev K Shrivastava, Elise Maudru, Gurdial Singh, Richard H Wightman, Keith M Morgan

**Affiliations:** 1 Department of Chemistry, University of Sunderland, Sunderland, SR1 3SD, UK; 2 Sciences & PE Department, Natural and Applied Sciences Building, Edge Hill University, Ormskirk, L39 4QP, UK (present address); 3 Department of Chemistry, The University of the West Indies, St. Augustine, Trinidad and Tobago (present address); 4 Department of Chemistry, Heriot-Watt University, Edinburgh, EH14 4AS, UK.

**Keywords:** carbasugars, cyclisation, cyclohexanes, 6-membered ring, stereoisomers

## Abstract

Treatment of 5-(*tert*-butyldimethylsilyl)-2,3-*O*-isopropylidene-D-ribose with lithium acetylides gave mixtures of *syn*- and *anti*-alkynols **2a–2c** which were separated following protection as methoxymethyl ethers. These were converted to the corresponding iodides which underwent 6-*exo*-*dig* radical cyclisation to afford chiral cyclohexanes and carbasugars. Oxidation of the primary alcohols **6a–b** gave the corresponding aldehydes which on treatment with Grignard reagents afforded a mixture of alcohols. The corresponding iodides underwent similar 6-*exo*-*dig* cyclisation to give fully functionalised cyclohexanes.

## Introduction

The use of carbohydrates as precursors for highly functionalised carbocyclic rings systems has found wide utility in organic synthesis [1–11]. In particular there is a large amount of literature devoted to the synthesis of cyclopentanes [12–13] that employs a 5-*exo*-*dig* ring closure of a radical onto an alkyne [14]. In contrast there are fewer reports of the corresponding 6-*exo*-*dig* cyclisation to prepare fully functionalised cyclohexanes and carbasugars. The reason for this may be inferred from the observation that the formation of six-membered rings by a radical ring closure is some 40 times slower than the counterpart 5-hexenyl cyclisation [15–19]. In the case of the 6-hexenyl radical this results in the attack of the acyclic radical on the tin reagent becoming a more effective process compared to the 5-hexenyl case. An additional problem is that a 1,5-hydrogen abstraction leading to a resonance stabilised allylic radical is favourable, a reaction which may in fact be synthetically useful. We have previously reported the synthesis of cyclohexanes and carbasugars that uses a 6-*exo*-*dig* cyclisation [20] and herein we report on our current endeavours in this area.

## Results and Discussion

**Scheme 1:**
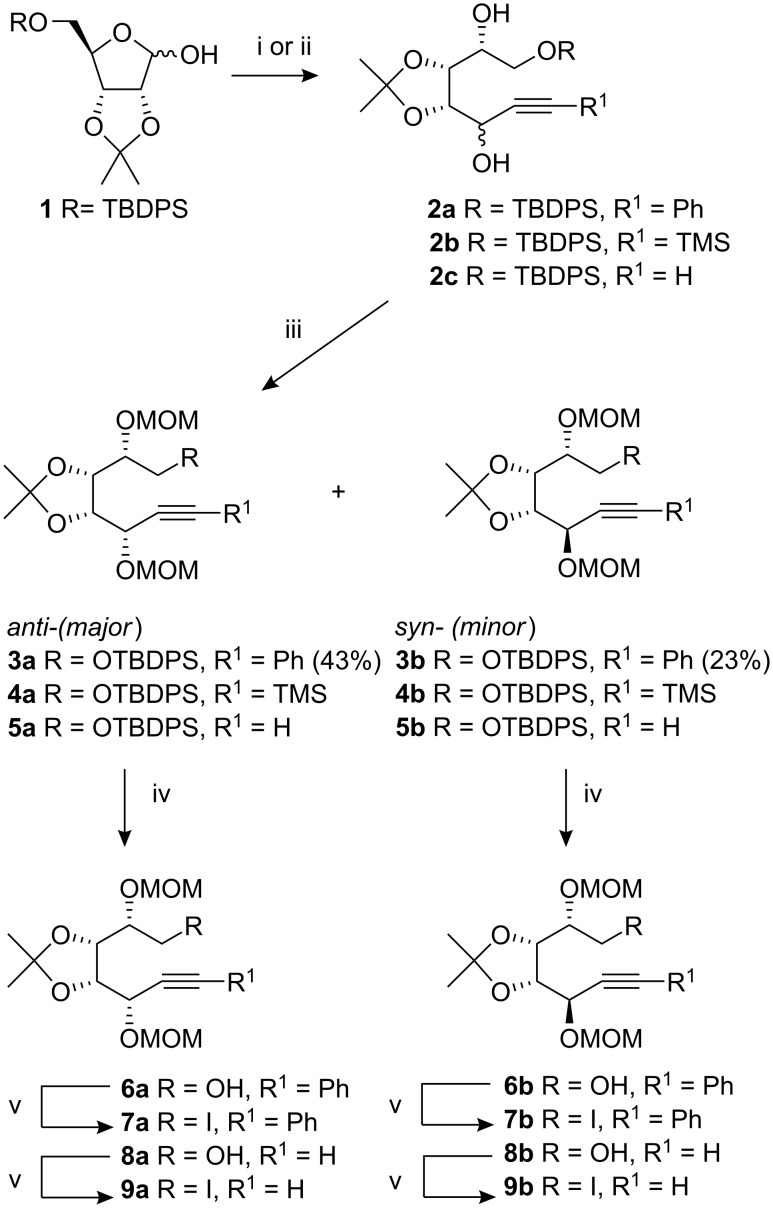
Synthesis of iodides for radical cyclisation. *Reagents and conditions:* (i) LiC≡CPh, THF, −78 °C to RT 12 h; (ii) LiC≡CTMS, THF, −78 °C; (iii) MOMCl, iPr_2_NEt, DCM, 0 °C to RT 48 h; (iv) TBAF, THF, RT, 12 h; (v) I_2_, PPh_3_, Im, PhMe, Δ.

Acetonisation [21] of D-ribose with acetone and hydrochloric acid afforded the corresponding acetonide (72%) which was converted into silyl ether **1** (83%) by treatment with *tert*-butyldiphenylsilyl chloride (TBDPSCl) and triethylamine using DMAP as the catalyst. The silylated D-riboses **1** were treated with lithium phenylacetylide to give an inseparable mixture of diastereoisomeric diols **2a** in 89% yield (Scheme 1). Analysis of the ^1^H NMR spectrum showed a 1:2 isomeric mixture of diastereoisomers, on the basis of integration of the *tert*-butyl group resonances, which were slightly separated (*syn*: 1.08 ppm and *anti*: 1.09 ppm).

The stereochemistry at the new chiral centre was assigned on the basis of our previous findings [22]. The mixtures of diols **2** were treated with chloromethyl methyl ether to afford the corresponding MOM ethers, in a combined yield of 66%. At this juncture we were able to separate the diastereoisomers, **3a** (*anti*) and **3b** (*syn*), by flash column chromatography on silica gel. Alternatively the *syn* isomer **3b** could be prepared almost exclusively using D-ribonolactone as the starting material [23].

Desilylation of **3a** was effective with TBAF in THF and afforded the primary alcohol **6a** in 87% yield, which was converted to the corresponding iodide **7a** with triphenylphosphine, imidazole and iodine [24]. Similar chemistry with the isomer **3b** gave the alcohol **6b** and subsequently the iodide **7b** in comparable yields.

**Scheme 2:**
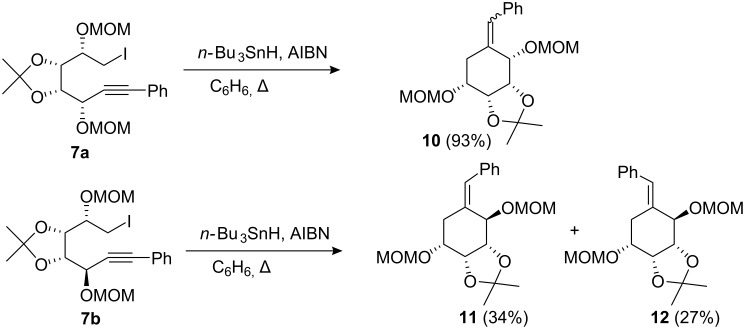
Radical cyclisation of compounds **7a** and **7b**.

With the availability of the iodides **7a** and **7b** we investigated their radical cyclisation reactions. Treatment of **7a** with tri-*n*-butyltin hydride and AIBN in refluxing benzene afforded (Scheme 2) the 6-*exo*-cyclisation [25–28] products **10** in 93% yield as an inseparable mixture of *E* and *Z* geometric isomers in a ratio of 1:1.6 as determined by ^1^H NMR analysis. The diastereomer **7b** on similar treatment underwent a 6-*exo*-cyclisation and afforded the corresponding cyclohexanes **11** and **12** in 61% yield. In this case we were able to separate the geometric *E* and *Z* isomers by silica gel chromatography, in a ratio of 3:2, respectively.

The major isomer **11** was assigned having the *Z* geometry about the double bond on the basis of NOE experiments. Irradiation of the vinylic resonance at δ 6.66 resulted in enhancement of the aromatic resonances of H-6α at δ 2.93, and H-6β at δ 2.39. Analysis of the ^1^H NMR data (Table 1) allowed assignment of the stereochemistry at C-2. A large coupling constant *J*_6α,5_ 11.8 Hz at δ 2.93 was observed, which suggests an *axial-axial* relationship between H-6α and H-5, whilst the resonance at δ 2.39, H-6β, showed a coupling constant *J*_6β,5_ 5.3 Hz indicative of an *axial*-*equatorial* relationship.

**Table 1:** 270 MHz ^1^H NMR of compound **11**.

Proton	Chemical shift (ppm)	Multiplicity	Coupling constants (Hz)

6β-H	2.39	dd	*J*_gem_ 12.5 and *J*_6β,5_ 5.3
6α-H	2.93	td	*J*_gem_ 12.5 and *J*_6α,5_ 11.8
5-H	3.88	dt	*J*_5,6β_ 5.3, *J*_5,6α_ 11.9 and *J*_5,3_ 4.6
4-H	4.20	t	*J*_4,5_ = *J*_4,3_ 5.3
3-H	4.54	t	*J*_3,4_ = *J*_3,2_ 5.3 and *J*_3,5_ 4.6
2-H	4.61	d	*J*_2,3_ 5.3
7-H	6.66	s	

Having been successful in our initial goal we turned our attention to the preparation of a cyclohexane that had an unsubstituted *exo*-methylene group. Treatment of the silylated ribose derivative with lithium trimethylsilylacetylide in THF gave the diols **2b**, in a combined yield of 45% along with the alkynes **2c** in 28% yield. The diastereomeric mixture **2b** was converted to the MOM ethers **4a** and **4b** that could be separated in a combined yield of 64% with an *anti:syn* ratio of 3.5:1. Both of these diastereoisomers were processed separately. Removal of the silyl protecting group of **4a** and **4b** gave the corresponding primary alcohols **8a** and **8b** in 96% and 91% yields, respectively. These compounds were identical to those obtained from **2c** after the diastereoisomers had been subjected to protection as MOM ethers on treatment with MOM chloride to give **5a** and **5b** followed by desilylation, the *anti:syn* ratio being 2.3:1.

**Scheme 3:**
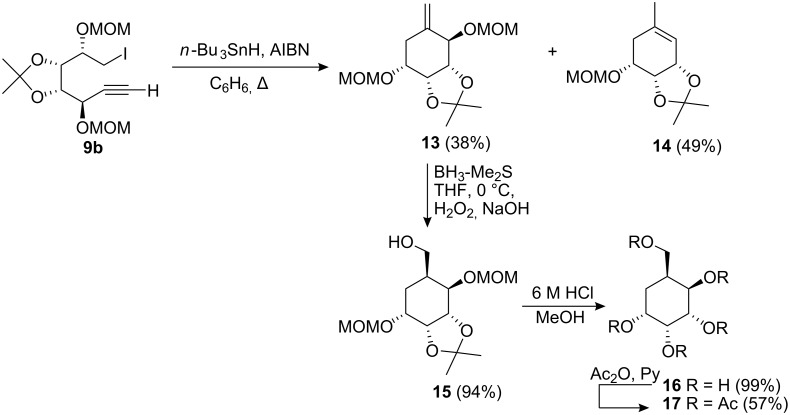
Radical cyclisation of compound **9b**.

The *syn*-product was correlated with the syn isomer **2c** [23]. Conversion of the alcohols **8a** and **8b** to the iodides **9a** and **9b** was accomplished by treatment with triphenylphosphine, imidazole and iodine in 73% and 78% yields, respectively. At this point we proceeded to investigate the 6-*exo* radical cyclisation of these iodides. The *syn*-isomer **9b** was treated with tri-*n*-butyltin hydride and AIBN in refluxing benzene, (Scheme 3) and gave the expected *exo*-methylene cyclohexane **13** in 38% yield along with the cyclohexene **14** in 49% yield where the OMOM group had been lost. The structure of **14** was clearly evident from its ^1^H NMR spectrum which had a resonance only for one MOM group in addition to the observation of a resonance at δ 5.33 due to the vinylic proton and a resonance at δ 1.73 due the methyl group.

**Figure 1:**
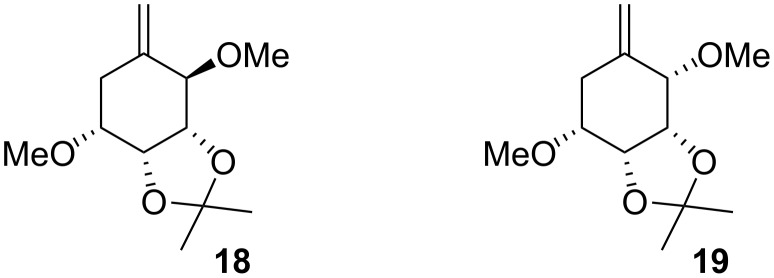
Structures of compounds **18** and **19**.

Following these findings we subjected the isomer **9a** to the same reaction conditions and we observed that the cyclisation was appreciably slower, taking 24 h to go to completion. However this reaction was much more selective in that we only isolated **14** in a yield of 99%. In this case the primary radical formed from the iodide **9a** undergoes a 6-*exo* cyclisation onto the alkyne, which results in the formation of a vinyl radical which is capable of abstracting a hydrogen from the methylene carbon of the OMOM group subsequently followed by β-scission [29–30] resulting in the formation of an allylic radical which gives rise to the observed product. The formation of **14** reflects the geometry of the vinyl radical and that these are bent with a bond angle of ca. 135° whilst in the case of an α-phenyl radical the geometry is linear [31]. In order to gain additional insight as to the geometries of the transition state(s) for these processes we undertook Monte Carlo calculations using much simpler structures (Figure 1), in order to simplify the computation, (**18** and **19**).

**Figure 2:**
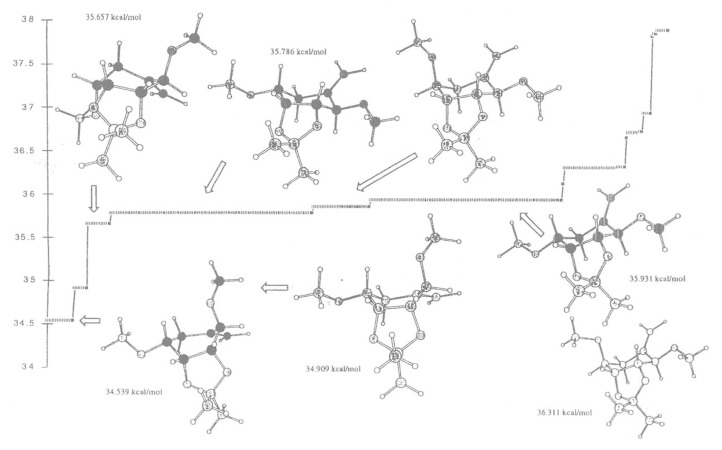
Monte Carlo search on the simplified “*trans*” structures (structures shown are within 2 kcal/mol).

**Figure 3:**
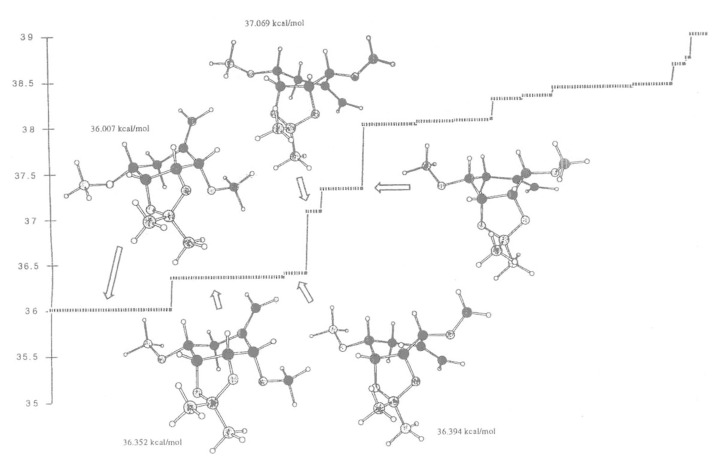
Monte Carlo search on the simplified “*cis*” structures (structures shown are within 2 kcal/mol).

At low energies of 34.539 and 34.909 kcal/mol (Figure 2), the “*trans*” isomer **18** favours a boat conformation, in which the carbon-carbon double bond is above the plane of the ring and the methoxy group is in *axial* position and as a consequence the vinyl radical cannot abstract a hydrogen radical from the methoxy group. However at higher energies 35.657 kcal/mol, the “*trans*” isomer adopts a boat conformation and the ring can flip to different conformations at 35.786 and 35.931 kcal/mol and allows the radical to abstract a hydrogen atom from the methoxy group (Figure 2). The “*cis*” isomer **19** favours the chair conformation at low energies (36.007 kcal/mol) but can flip to the boat conformation more easily (36.394 kcal/mol) where the vinyl radical (Figure 3) can abstract the hydrogen atom from the methoxy group.

**Scheme 4:**
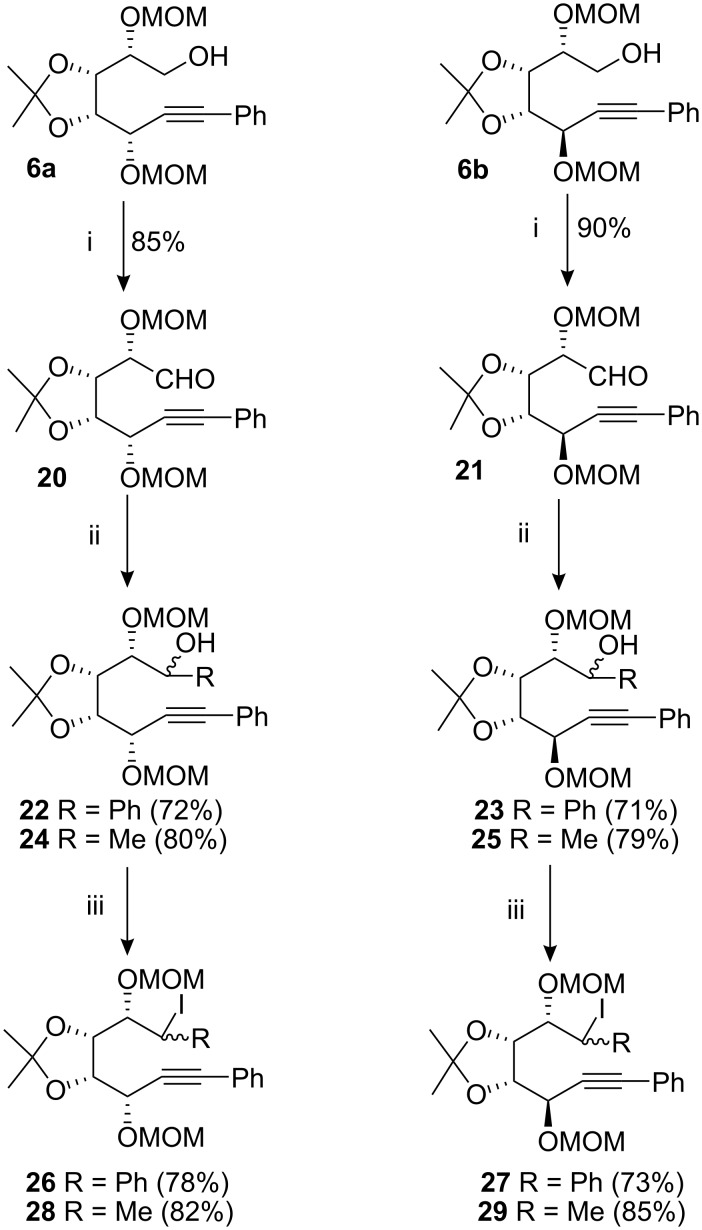
Synthesis of iodides from compounds **6a** and **6b**. *Reagents and conditions*: (i) PCC, 4 Å molecular sieves, DCM, RT, 2 h; (ii) PhMgCl or MeMgBr, THF, 0 °C, 1 h; (iii) PPh_3_, imidazole, I_2_, toluene, 60 °C, 2 h.

Having established that the 6-*exo* cyclisation was of synthetic value we proceeded to investigate the possibility of carrying out such a process using a secondary radical and thereby obtaining access to a cyclohexane ring that had substituents on all six carbons. Thus the alcohols **6a** and **6b** were oxidised most efficiently with PCC [32–37] to afford the corresponding aldehydes **20** and **21** in 85% and 90% yield, respectively. Both these aldehydes were found to be somewhat unstable at room temperature, as evidenced by their polymerisation and hence were used in the subsequent reaction as soon as possible. The aldehyde **20** was treated with phenylmagnesium chloride in THF and gave a mixture of two-diastereoisomeric alcohols **22** in 72% yield, which were separated using flash chromatography on silica gel. The stereochemistry at the newly created chiral centre could not be defined at this juncture. However, this was deemed to be unnecessary, as both of these diastereoisomeric alcohols would give rise to the same secondary radical and as a result we employed the diastereomeric mixture **22** in the subsequent chemistry. This mixture of alcohols **22** was converted to the corresponding iodides [25–30] **26** on treatment with triphenylphosphine, imidazole and iodine in 78% yield. The iodides **26** were prone to rapid decomposition even when stored at 4 °C as evidenced by TLC and ^1^H, ^13^C NMR analysis. Similar chemistry was performed with aldehyde **21** which gave a mixture of secondary alcohols **23** in 71% yield and subsequently the mixture of alcohol gave the corresponding iodide **27** in 73% yield. Similar treatment of the aldehydes **20** and **21** with methylmagnesium bromide afforded the analogous diols **24** and **25** in 80% and 79% yields, respectively; these diols were converted to the iodides **28** and **29** employing similar chemistry (Scheme 4).

**Scheme 5:**
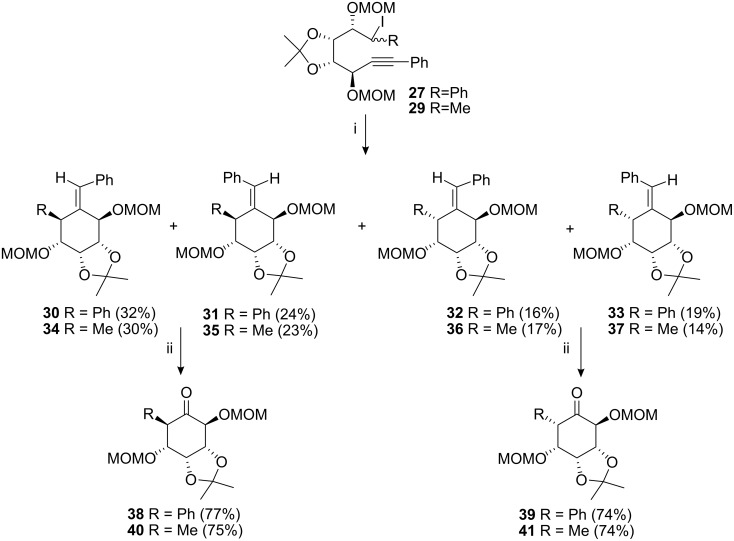
Radical cyclisation of compounds **27** and **29**. *Reagents and conditions*: (i) Bu_3_SnH, AIBN, benzene, 80 °C, 3 h; (ii) DCM, MeOH, O_3_, −78 °C, 40 min.

**Scheme 6:**
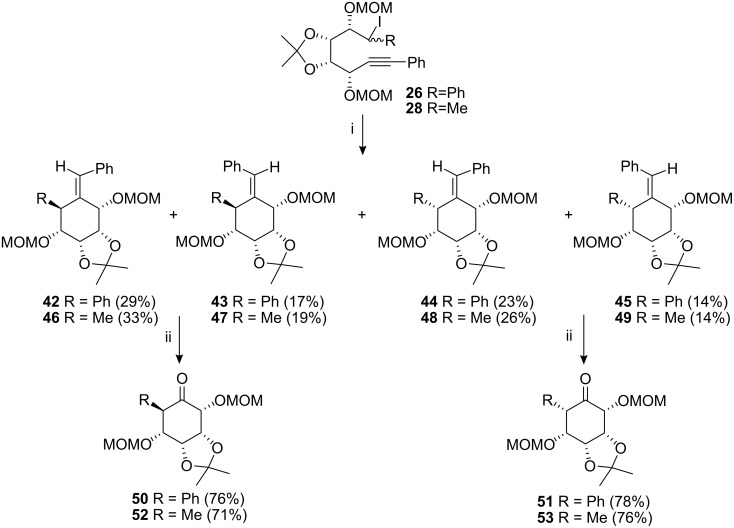
Radical cyclisation of compounds **26** and **28**. *Reagents and conditions:* (i) Bu_3_SnH, AIBN, benzene, 80 °C, 3 h; (ii) DCM, MeOH, O_3_, −78 °C, 40 min.

Radical cyclisation of the iodides **27** was conducted via dropwise addition of tri-*n*-butyltin hydride in refluxing benzene, using AIBN as a radical initiator. This resulted in the formation of the two pairs of diastereoisomers at C-2 and C-6 along with two pairs of geometric isomers in a combined yield of 91%, (Scheme 5). We were able to separate these isomers successfully by flash chromatography, eluting with light petroleum-diethyl ether (3:1 to 1:1). A similar outcome was obtained for the isomers **26** (Scheme 6) and resulted in a total cyclisation yield of 83%. We also investigated the cyclisation reactions of the “methyl” analogues **28** and **29** and these gave a similar outcome, (Scheme 6 and Scheme 5). Ozonolysis of the pair of geometric isomers **30** and **31** afforded the protected ketone **38** in 77% yield.

**Scheme 7:**
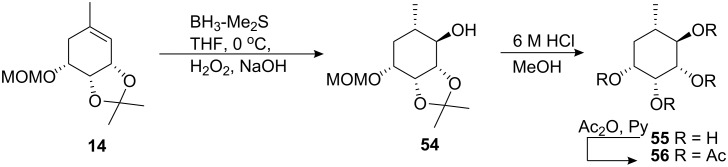
Synthesis of compounds **55** and **56**.

The diastereomeric ketone **39** was obtained in 74% yield after similar treatment of **32** and **33**. Removal of the MOM and isopropylidene with 6 M HCl resulted in the aromatisation of the ring system. A similar outcome was obtained if we conducted acetylation of the crude reaction mixture in the presence of acetic anhydride and pyridine. In contrast to these findings the protecting groups of the cyclohexane **54**, (Scheme 7), could be successfully removed on treatment using these conditions. Hydroboration of cyclohexene **14** obtained from cyclisation of **9a**, following oxidative workup gave the cyclohexanol **54** in 77% yield. Removal of the isopropylidene and MOM protecting groups afforded a fully deprotected carba-β-D-rhamnose **55** in 99% yield. The structural integrity of **55** was established by acetylation with acetic anhydride and pyridine which gave the tetraacetate **56** in 99% yield.

## Conclusion

We have established that carbohydrate derived alkynes undergo 6-*exo* radical cyclisation and provide access to fully functionalised cyclohexanes and carbasugars which are not easily accessible via other routes.

## Supporting Information

File 1 Experimental and Data
