# Supplementary material for: Synthesis of chiral cyclohexanes and carbasugars by 6-exo-dig radical cyclisation reactions
Source: Beilstein J Org Chem. 2008 Nov 19;4:43. doi: 10.3762/bjoc.4.43 (PMC2605619; doi:10.3762/bjoc.4.43)
Supplement: File 1 — Experimental and Data [file Beilstein_J_Org_Chem-04-43-s001.doc]

# Experimental and Data

**Synthesis of chiral cyclohexanes and carbasugars by** 6‑***exo-dig* radical cyclisation reactions**

Rajeev K. Shrivastava^*,1,2^, Elise Maudru^1^, Gurdial Singh^*,1,3^, Richard H. Wightman^4^ and Keith M. Morgan^4^

Address: ^1^Department of Chemistry, University of Sunderland, Sunderland, SR1 3SD, UK, ^2^Sciences & PE Department, Natural and Applied Sciences Building, Edge Hill University, Ormskirk, L39 4QP, UK (present address), ^3^Department of Chemistry, The University of the West Indies, St. Augustine, Trinidad and Tobago (present address) and ^4^Department of Chemistry, Heriot-Watt University, Edinburgh, EH14 4AS, UK.

Email: Rajeev K. Shrivastava^*^ - [Rajeev.Shrivastava@edgehill.ac.uk](mailto:Rajeev.Shrivastava@edgehill.ac.uk); Gurdial Singh^*^ - [Gurdial.Singh@sta.uwi.edu](mailto:Gurdial.Singh@sta.uwi.edu)

* Corresponding author

**General**

IR spectra were recorded on a UNICAM research series FT-instrument. ^1^H NMR spectra were measured at 270 MHz with a JEOL GSX 270 FT NMR or at 400 MHz with a JEOL GX 400 FT NMR instrument. Chemical shifts were measured relative to internal tetramethylsilane (δ = 0). ^13^C NMR spectra were recorded at 67.8 MHz or 100 MHz with δ TMS = 0 as internal reference or the solvent as internal reference. CDCl_3_ was used to dissolve samples for the determination of NMR spectra unless otherwise stated. Melting points were measured on a Gallenkamp capillary melting point apparatus and are uncorrected. Specific rotations were measured in chloroform solution using a Bellingham & Stanley ADP 220 polarimeter and the values are given in 10^-1^ deg·cm^2^·g^-1^. Mass spectra and high-resolution mass spectra were carried out by the EPSRC National Mass Spectrometry Centre in the Department of Chemistry at the University of Wales, Swansea. Flash chromatography was carried out on Fluka silica gel 60 (230–400 mesh). Thin layer chromatography was carried out using pre-coated aluminium plates (Merck Kieselgel 60 F_254_) which were visualised under UV light and then using either acidic ammonium molybdate [ammonium molybdate(VI) tetrahydrate (25 g), conc. H_2_SO_4_ (50 ml) and water (450 ml)] or basic aqueous potassium permanganate [potassium permanganate (6 g), potassium carbonate (40 g), NaOH (0.5 g) and water (610 ml)] as appropriate. THF was distilled from sodium-benzophenone in a recycling still. Dichloromethane, toluene, benzene and pyridine were distilled from calcium hydride and stored over 3Å molecular sieves. Petroleum ether (boiling range 40–60 °C) was distilled prior to use; other organic solvents and reagents were purified by standard methods. All anhydrous reactions were carried out in flame- or oven dried apparatus under argon or nitrogen atmosphere. Anhydrous transfers were carried out using standard syringe techniques.

**7-*O-tert*-Butyldiphenylsilyl-1,2-dideoxy-4,5-*O*-isopropylidene-1-phenyl-d-hept-1-ynitols (2a):** Phenylacetylene (13.21 g, 14.21 ml, 129.37 mmol) was dissolved in dry THF (60 ml) at −78 °C under argon. To this solution *n*-BuLi (1.6 M in hexane, 72.8 ml, 116.43 mmol) was added and the mixture was stirred for 40 min. Following this 5-*O*-*tert*-butyldiphenylsilyl-2,3-*O*-isopropylidene-d-ribose (**1**) (11.07 g, 25.87 mmol) dissolved in dry THF (70 ml) was added dropwise over 20 min; maintaining the temperature at −78 °C. The solution was stirred and allowed to warm up to room temperature and stirring continued for 12 h. The solvent was evaporated under reduced pressure and the residue was diluted with ethyl acetate (250 ml), washed with water (200 ml) and extracted with ethyl acetate (2 x 250 ml). The organic layers were combined, dried (Na_2_SO_4_), concentrated and purified by flash chromatography on silica gel using light petroleum/diethyl ether (2:1) as eluent to afford the title compound **2a** (12.18 g, 89%) as a colourless oil, [α]_D_ 0 (*c* 1.2 in CHCl_3_); ν_max_(film)/cm^-1^ 3413 (OH), 3072, 3018, 2958, 2934, 2877, 2854, 2228 (C≡C), 1646, 1598, 1490, 1463, 1428, 1382, 1317, 1216, 1112, 1072, 1008 and 946; δ_H_ (270 MHz) (mixture of diastereoisomers minor *altro*:major *allo* 1:2) 1.08 (3 H, s, minor CMe_3_), 1.09 (6 H, s, major CMe_3_), 1.35 (4 H, s, major CMe_2_), 1.37 (1 H, s, minor CMe_2_), 1.47 (1 H, s, minor CMe_2_), 3.19 (0.4 H, bs, minor OH), 3.21 (0.6 H, bs, major OH), 3.81 (1 H, m, major and minor C*H*HOSi), 3.94 (1 H, m, major and minor CH*H*OSi), 4.05 (1 H, m, major and minor C*H*CH_2_OSi), 4.20 (0.6 H, d, *J* 5.6 and 10.2, major and minor C*H*OR), 4.37 (0.4 H, d, *J* 5.9 major and 9.8, minor C*H*OR), 4.41 (0.8 H, d, *J* 9.9, major C*H*OR), 4.93 (0.2 H, d, *J* 5.8, minor C*H*OR), 5.30 (1 H, d, *J* 8.5, C≡C-C*H*(OH)), 7.29–7.36 (3 H, m, Ph), 7.39–7.46 (9 H, m, Ph), 7.65–7.70 (3 H, m, Ph); δ_C_ (67.8 MHz) (major, *allo*- diastereoisomer) 24.82 (1 C, *C*Me_3_), 25.44 (1 C, Me), 26.78 (3 C, *C*Me_3_), 27.61 (Me), 61.88, 65.14, 69.33, 76.53, 77.47 (5 C, CH_2_OSi, 2 x CHOR, 2 x CHOH), 85.51 and 87.76 (2C, carbon triple bond), 109.28 (*C*Me_2_), 122.64 (1 C, Ph), 127.76 (2 C, Ph), 127.80 (3 C, Ph), 127.84 (2 C, Ph), 128.13 (2 C, Ph), 128.21 (Ph), 128.35 (Ph), 131.88 (2 C, Ph), 135.45 (2 C, Ph), 135.51 (2 C, Ph) and unresolved; δ_C_ (67.8 MHz) (minor, *altro*- diastereoisomer) 24.65 (*C*Me_3_), 25.31 (1 C, Me), 26.73 (3 C, *C*Me_3_), 27.49 (Me), 61.82, 65.06, 69.27, 76.41, 77.36 (5 C, CH_2_OSi, 2 x CHOR, 2 x CHOH), 85.32 and 87.59 (2C, C≡C), 108.98 (*C*Me_2_), 121.96 (Ph), 127.43 (2 C, Ph), 127.28 (3 C, Ph), 127.59 (2 C, Ph), 128.23 (2 C, Ph), 128.12 (Ph), 128.23 (Ph), 131.76 (2 C, Ph), 134.48 (2 C, Ph), 134.49 (2 C, Ph); *m/z* (CI, NH_3_) 548 (M + NH_4_^+^), 531 (M^+^) 377 (M^+^−2Ph), 202, 157,143,78,58,52; [Found M + NH_4_^+^, 548.2830 C_32_H_42_O_5_NSi requires M + NH_4_^+^, 548.2832].

**7-*O*-*tert*-Butyldiphenylsilyl-1,2-dideoxy-4,5-*O*-isopropylidene-1-trimethylsilyl-d-hept-1-ynitols (2b) and 7-*O*-*tert*-butyldiphenylsilyl-1,2-dideoxy-4,5-*O*-isopropylidene-d-hept-1-ynitols (2c)**: In a 250 ml round bottom flask, dry THF (80 ml) was cooled to −78 °C and trimethylsilylacetylene (6.2 ml, 43.71 mmol) was added, followed by *n*-BuLi (1.6 M in hexanes, 23.5 ml, 37.47 mmol). After 40 min, 5-*O*-*tert*-butyldiphenylsilyl-2,3-*O*-isopropylidene-α,β-d-ribofuranose (**1**) (5.34 g, 12.49 mmol) dissolved in dry THF (20 ml) was added to the mixture at −78 °C *via* canula, and the mixture allowed to warm up to room temperature. After 12 h, the reaction mixture was concentrated and the residue was diluted in ethyl acetate (200 ml), washed with water (100 ml) and extracted with ethyl acetate (2 x 200 ml). The organic layers were combined, dried (Na_2_SO_4_), concentrated and purified by flash chromatography on silica gel using light petroleum/diethyl ether (3:1) as eluent to afford the title compounds **2b** (2.93 g, 45%) and **2c** (1.56 g, 28%) as yellowish oils.

*R_f_* = 0.5 in light petroleum/diethyl ether (2:1) **2b**, [α]_D_ −8.1 (*c* 17.95 in CHCl_3_); ν_max_(film)/cm^-1^ 3392 (OH), 3072, 3050, 2958, 2933, 2896, 2859, 2177 (C≡C), 1589, 1471, 1427, 1380, 1249, 1218, 1168, 1112, 1068, 1008, 941, 844, 761; δ_H_ (270 MHz) (mixture of diastereoisomers α,β as 1:3.6) 0.18 (9 H, s, major and minor SiMe_3_), 1.07 (7.2 H, s, major CMe_3_), 1.09 (1.8 H, s, minor CMe_3_), 1.32 (2.5 H, s, major Me), 1.33 (2.5 H, s, major Me), 1.44 (0.5 H, s, minor Me), 1.47 (0.5 H, s, minor Me), 3.07 (0.21 H, d, *J* 5.27, minor OH), 3.16 (0.64 H, d, *J* 3.96, major OH), 3.63–3.69 (0.22 H, dd, *J* 3.3 and 11.21, minor CH_2_OSi), 3.73–3.81 (1.82 H, dd, *J* 3.95 and 9.89, major CH_2_OSi), 3.88–3.99 (1.67 H, m, C*H*CH_2_OSi and major OH), 4.12–4.18 (0.73 H, dd, *J* 5.28 and 9.89, C*H*CHCH_2_OSi), 4.24–4.44 (1.57 H, m, major C*H*CHC≡, minor C*H*CHC≡ and minor OH), 4.60–4.73 (1.25 H, m, major C*H*C≡), 5.33 (0.21 H, d, *J* 10.56, minor C*H*C≡), 7.33–7.47 (6.7 H, m, *meta-* and *para-*Ph), 7.64–7.69 (4.4 H, m, *ortho-*Ph); δ_C_ (67.8 MHz) (major, α anomer) −0.06 (3 C, SiMe_3_), 19.29 (1C, *C*Me_3_), 25.40 (1 C, Me), 26.87 (3 C, C*Me_3_*), 27.52 (1 C, Me), 61.98, 65.32, 69.43, 76.40 and 80.13 (5 C, *C*H_2_ and 4 x CHOR), 87.15 and 90.62 (2 C, C≡C), 109.19 (1 C, *C*Me_2_), 127.77 (2 C, Ph), 127.80 (2 C, Ph), 127.85 (1 C, Ph), 129.90 (1 C, Ph), 129.94 (1 C, Ph), 132.99 (1 C, Ph), 135.52 (2 C, Ph), 135.57 (2 C, Ph); δ_C_ (37.8 MHz) (minor, β anomer) 11.34 (3 C, SiMe_3_), 18.72 (1 C, *C*Me_3_), 24.93 (1 C, Me), 27.14 (3 C, C*Me_3_*), 29.04 (1 C, Me), 61.98, 65.51, 69.43, 76.60 and 79.14 (5 C, CH_2_, 4 x CHOR), 87.32 and 90.56 (2 C, C≡C), 104.30 (1 C, *C*Me_2_), 127.77 (1 C, Ph), 128.02 (1 C, Ph), 128.06 (2 C, Ph), 130.19 (1 C, Ph), 130.36 (1 C, Ph), 133.05 (1 C, Ph), 134.24 (1 C, Ph), 135.20 (1 C, Ph), 135.37 (1 C, Ph), 135.74 (2 C, Ph); *m/z* (CI, NH_3_) 544 (M + NH_4_^+^), 527 (MH^+^), 392, 313, 90. [Found M + NH_4_^+^ 544.2930. C_29_H_46_O_5_NSi_2_ requires 544.2915].

*R_f_* = 0.26 in light petroleum/diethyl ether (2:1) **2c**, [α]_D_ −9.5 (*c* 8.9 in CHCl_3_); ν_max_(film)/cm^-1^ 3386 (OH), 3305 (C≡C-H), 3070, 3030, 2985, 2935, 2892, 2857, 1724, 1465, 1427, 1380, 1245, 1218, 1164, 1110, 1068, 871, 821; δ_H_ (270 MHz)(mixture of diastereoisomers α,β as 1:1.8) 1.08 (9 H, s, major and minor CMe_3_), 1.31 (1.77 H, s, major Me), 1.33 (1.77 H, s, major Me), 1.35 (1.25 H, s, minor Me), 1.45 (1 H, s, minor Me), 2.47 (0.36 H, d, *J* 1.98, minor C≡C-H), 2.50 (0.66 H, d, *J* 1.98, major C≡C-H), 2.98 (0.43 H, d, *J* 5.28, minor OH), 3.28 (0.63 H, d, *J* 3.3, major OH), 3.44–3.54 (0.8 H, m, major C*H*HOSi), 3.73–3.83 (0.9 H, m, major CH*H*OSi), 3.88–3.93 (1.65 H, dt, *J* 3.3 and 10.56, major C*H*CH_2_OSi and major OH), 4.09–4.18 (1.1 H, m, minor OH, minor C*H*CH_2_OSi, minor C*H*CHCH_2_OSi), 4.26–4.31 (0.75 H, m, major C*H*CHCH_2_OSi), 4.37 (1 H, dd, *J* 3.3 and 6.6, major and minor C*H*CHC≡C), 4.65 (0.74 H, m, major C*H*C≡C), 4.76 (0.47 H, dt, *J* 2.64 and 9.24, minor C*H*C≡), 7.36–7.47 (6 H, m, *para-* and *meta-*Ph), 7.63–7.68 (4 H, m, *ortho-*Ph); *m/z* (CI, NH_3_) 472 (M + NH_4_^+^), 336, 319, 274, 196, 94 and 78. [Found M + H^+^ 455.2247. C_26_H_35_O_5_Si requires 455.2254].

**7-*O-tert*-Butyldiphenylsilyl-1,2-dideoxy-3,6-di-*O*-methoxymethyl-4,5-*O*-isopropylidene-1-phenyl-d-*allo*-hept-1-ynitol (3a)** **and 7-*O-tert*-butyldiphenylsilyl-1,2-dideoxy-3,6-di-*O*-methoxymethyl-4,5-*O*-isopropylidene-1-phenyl-d-*altro*-hept-1-ynitol (3b):** Compound **2a** (4.13 g, 7.8 mmol) was dissolved in DCM (50 ml) and cooled to 0 °C. To this *N,N-*diisopropylethylamine (10.07 g, 13.58 ml, 78 mmol) was added, followed by chloromethylmethyl ether (6.24 g, 5.9 ml, 78 mmol). The mixture was allowed to warm to room temperature and stirred for 48 h. The solvent was evaporated and the residue was diluted with ethyl acetate (150 ml). The resultant solution was washed with water (100 ml) and the aqueous phase extracted with ethyl acetate (2 x 150 ml). The organic layers were combined, dried over Na_2_SO_4_ and concentrated. The residue was purified by flash chromatography on silica gel, using light petroleum/diethyl ether (6:1) as eluent to afford the two diastereoisomers **3a** (2.07 g, 43%) and **3b** (1.11 g, 23%) as colourless oils.

*More polar* **3a** *(anti product). R_f_* = 0.30 in light petroleum/diethyl ether (3:1); [α]_D_ +74.7 (*c* 0.5 in CHCl_3_); ν_max_ (film)/cm^-1^ 3073, 3052, 2988, 2937, 2887, 2858, 2824, 2238 (carbon triple bond), 1645, 1594, 1494, 1421, 1439, 1378, 1327, 1258, 1213, 1156, 1109, 1049 and 923; δ_H_ (270 MHz) 1.09 (9 H, s, CMe_3_), 1.35 (3 H, s, Me), 1.52 (3 H, s, Me), 3.39 (3 H, s, OMe), 3.41 (3 H, s, OMe), 3.67 (1 H, dd, J 4.0 and 12.0, C*H*HOSi), 3.96 (1 H, dd, J 2.5 and 11.3, CH*H*OSi), 4.13–4.17 (1 H, m, C*H*CH_2_OSi), 4.39 (1 H, dd, *J* 2.5 and 7.1, C*H*CHC triple bond), 4.61 (1 H, dd, *J* 6.8 and 8.4, C*H*CHCH_2_), 4.75 (1 H, d, *J* 6.8, OCH*H*O), 4.81 (1 H, d, *J* 6.8, *OCH*H*O*), 4.83 (1 H, d, *J* 6.6, *OCH*H*O*), 4.98 (1 H, d, *J* 2.3, CHC triple bond), 5.07 (1 H, d, *J* 6.9, *OC*H*HO*), 7.38 (11 H, m, Ph), 7.68 (4 H, m, Ph); δ_C_ (67.8 MHz) 19.60 (1 C, *C*Me_3_), 25.13 (1 C, Me), 26.89 (1 C, Me), 26,96 (3 C, C*Me_3_*), 55.77 and 56.21 (2 C, 2 x O*Me*), 63.69 (1 C, CH_2_) 65.87, 74.39, 76.58 and 79.18 (4 C, 4 x *C*HOR), 87.88 and 88.89 (2 C, Carbon triple bond), 95.09 and 96.19 (2 C, 2 x O*C*H_2_O), 110.11 (1 C, *C*Me_2_), 127.71 (6 C, Ph), 129.75 (4 C, Ph), 133.30 (2 C, Ph) and 135.53 (6 C, Ph); *m/z* (CI, NH_3_) 636.6 (M + NH_4_^+^), 557.5 (M^+^ −Ph), 274.4 (-*tert*-butyldiphenylsilyl), 216.3, 196.3, 171.3, 169.3, 167.3 and 76.4 [Found: M + NH_4_^+^ 636.3360. C_36_H_50_O_7_NSi requires 636.3357].

*Less polar* **3b** *(syn product). R_f_* = 0.44 in light petroleum/diethyl ether (3:1); [α]_D_ −65.4 (*c* 0.6 in CHCl_3_); ν_max_ (film)/cm^-1^ 3074, 3053, 2992, 2943, 2887, 2859, 2825, 2230 (C≡C), 1645, 1592, 1494, 1471, 1439, 1376, 1328, 1254, 1210, 1108 and 1024; δ_H_ (270 MHz) 1.09 (9 H, s, CMe_3_), 1.35 (3 H, s, Me), 1.51 (3 H, s, Me), 3.30 (3 H, s, OMe), 3.39 (3 H, s, OMe), 3.94 (2 H, m, C*H*_2_OSi), 4.19–4.21 (1 H, m, C*H*CH_2_OSi), 4.43 (1 H, d, *J* 6.2, C*H*CHC triple bond), 4.58 (1 H, d, *J* 6.2, C*H*CHCH_2_), 4.75 (3 H, m, C*H*C triple bond and OC*H*_2_O), 4.89 (1 H, d, *J* 6.9, *OCH*H*O*), 5.02 (1 H, d, *J* 6.3, *OC*H*HO*), 7.28–7.43 (12 H, m, Ph), 7.69–7.74 (3 H, m, Ph); δ_C_ (67.8 MHz) 19.57 (1 C, *C*Me_3_), 25.26 (1 C, Me), 26.76 (1 C, Me), 26,93 (3 C, C*Me_3_*), 55.78 and 55.93 (2 C, 2 x O*Me*), 64.48 (1 C, CH_2_) 65.78, 76.32, 76.94 and 78.33 (4 C, 4 x *C*HOR), 85.29 and 87.48 (2 C, Carbon triple bond), 95.03 and 96.38 (2 C, 2 x O*C*H_2_O), 109.78 (1 C, *C*Me_2_), 127.04 (2 C, Ph), 127.68 (2 C, Ph), 128.39 (1 C, Ph), 128.68 (2 C, Ph), 129.08 (2 C, Ph), 129.17 (1 C, Ph), 131.89 (2 C, Ph), 135.86 (2 C, Ph), 135.98 (2 C, Ph) and 136.06 (2 C, Ph); *m/z* (CI, NH_3_) 636.3 (M + NH_4_^+^) 557.3 (M^+^ − Ph), 274.2 (M^+^ -*tert*-butyldiphenylsilyl), 216(-OMOM), 196.1, 185.1, 169.1, 167.1, 157.1, 108.1 and 78.1 [Found: M + NH_4_^+^ 636.3360. C_36_H_50_O_7_NSi requires 636.3357].

**7-*O*-*tert*-Butyldiphenylsilyl-1,2-dideoxy-3,6-di-*O*-methoxymethyl-4,5-*O*-isopropylidene-1-trimethylsilyl-d-*allo*-hept-1-ynitol (4a) and 7-*O*-*tert*-butyldiphenylsilyl-1,2-dideoxy-3,6-di-*O*-methoxymethyl-4,5-*O*-isopropylidene-1-trimethylsilyl-d-*altro*-hept-1-ynitol (4b):** 7-*O*-*tert*-Butyldiphenylsilyl-1,2-dideoxy-4,5-*O*-isopropylidene-1-trimethylsilyl-d-hept-1-ynitols (**2b**, 1.871 g, 3.56 mmol) were dissolved in dry DCM (50 ml) and placed at 0 °C. *N,N*-Diisopropylethylamine (4.6 g, 6.2 ml, 35. 6 mmol) was added followed by chloromethylmethyl ether (2.86 g, 2.7 ml, 35.8 mmol). The reaction mixture was left at 0 °C for 20 min and warmed up to room temperature. After 48 h, the solvent was evaporated, the residue dissolved in ethyl acetate (100 ml), washed with water (75 ml) and extracted with ethyl acetate (2 x 100 ml), dried (Na_2_SO_4_), concentrated and the residue purified by flash chromatography on silica gel using light petroleum/diethyl ether (6:1) as eluent to afford the 2 diastereoisomers **4a** (1.099 g, 50%) and **4b** (0.303 g, 14%) as colourless oils.

*R_f_* = 0.32 in light petroleum/diethyl ether (4:1) **4a**, [α]_D_ +28.7 (*c* 6.75 in CHCl_3_); ν_max_(film)/cm^-1^ 2956, 2933, 2892, 2857, 2823, 2175 (C≡C), 1589, 1471, 1428, 1378, 1251, 1214, 1151, 1105, 1037, 919, 844, 823, 759 and 742; δ_H_ (270 MHz) 0.14 (9 H, s, SiMe_3_), 1.07 (9 H, s, C*Me_3_*), 1.37 (3 H, s, Me), 1.55 (3 H, s, Me), 3.38 (3 H, s, OMe), 3.40 (3 H, s, OMe), 3.83–3.89 (1 H, dd, *J* 3.96 and 11.22, C*H*HOSi), 4.01 (1 H, dd, *J* 1.98 and 11.21, CH*H*OSi), 4.16–4.21 (1 H, m, *J* 1.98, 2.64 and 9.24, C*H*CH_2_OSi), 4.43 (1 H, dd, *J* 1.97, 2.64 and 6.59, 7.26, C*H*CHCH_2_OSi), 4.51–4.57 (1 H, dd, *J* 7.26 and 8.57, C*H*CHC≡), 4.63 (1 H, d, *J* 6.59, OCH*H*O), 4.73 (1 H, d, *J* 1.98, C*H*C≡), 4.77 (1 H, d, *J* 6.6, OC*H*HO), 4.84 (1 H, d, *J* 7.26, OC*H*HO), 5.00 (1 H, d, *J* 6.6, OCH*H*O), 7.38–7.43 (6 H, m, *meta-* and *para-*Ph), 7.69–7.74 (4 H, m, *ortho-*Ph); δ_C_ (67.8 MHz) −0.34 (3 C, SiMe_3_), 19.27 (1 C, *C*Me_3_), 24.87 (1 C, Me), 26.59 (1 C, Me), 26.78 (3 C, *Me* and C*Me_3_*), 55.69 and 56.20 (2 C, 2 x OMe), 63.50 (1 C, CH_2_), 65.75 (1 C, C≡*C*-TMS), 74.22, 76.39, 76.53 and 77.18 (4 C, 4 x CHOR), 78.80 (1 C, *C*≡C-TMS), 93.81 and 96.38 (2 C, 2 x O*C*H_2_O), 108.93 (1 C, *C*Me_2_), 127.55 (2 C, Ph), 127.64 (2 C, Ph), 129.59 (1 C, Ph), 129.64 (1 C, Ph), 133.43 (1 C, quat. Ph), 133.54 (1 C, quat. Ph), 135.66 (2 C, Ph) and 135.74 (2 C, Ph); *m/z* (CI, NH_3_) 632 (M + NH_4_^+^), 583, 553, 525, 463, 431, 197 and 90. [Found M + NH_4_^+^ 632.3440. C_33_H_54_NO_7_Si_2_ requires 632.3440].

*R_f_* = 0.45 in light petroleum/diethyl ether (4:1) **4b**, [α]_D_ −8.2 (*c* 17.6 in CHCl_3_); ν_max_(film)/cm^-1^ 2958, 2933, 2892, 2857, 2823, 2173 (C≡C), 1589, 1471, 1428, 1378, 1251, 1214, 1153, 1112, 1039, 919, 846, 740; δ_H_ (270 MHz) 0.17(9 H, s, SiMe_3_), 1.07 (9 H, s, CMe_3_), 1.35 (3 H, s, Me), 1.37 (3 H, s, Me), 3.35 (3 H, s, OMe), 3.38 (3 H, s, OMe), 3.92 (2 H, m, C*H_2_*OSi), 4.17 (1 H, q, *J* 3.3, C*H*CH_2_OSi), 4.34–4.39 (1 H, t, *J* 6.59, C*H*CHCH_2_OSi), 4.45–4.49 (2 H, dd, *J* 3.95 and 5.93, C*H*CHC≡ and C*H*C≡), 4.62 (1 H, d, *J* 6.6, OC*H*HO), 4.75 (1 H, d, *J* 6.59, OCH*H*O), 4.83 (1 H, d, *J* 7.26, OC*H*HO), 4.92 (1 H, d, *J* 6.6, OCH*H*O), 7.38 (6 H, m, *para-* and *meta-*Ph), 7.69–7.73 (4 H, m, *ortho-*Ph); δ_C_ (37.8 MHz) −0.31 (3 C, SiMe_3_), 19.26 (1 C, *C*Me_3_), 25.16 (1 C, Me), 26.52 (1 C, Me), 26.82 (3 C, C*Me_3_*), 55.77 and 55.85 (2 C, 2 x OMe), 64.48 (1 C, CH_2_OSi), 65.69 (1 C, C≡*C*-TMS), 73.55, 76.21, 76.37 and 77.24 (4 C, 4 x CHOR), 78.60 (1 C, *C*≡C-TMS), 94.32 and 96.69 (2 C, 2 x OCH_2_O), 108.93 (1 C, *C*Me_2_), 127.58 (2 C, Ph), 127.65 (3 C, Ph), 129.56 (1 C, Ph), 129.62 (1 C, Ph), 135.66 (3 C, Ph), 135.74 (2 C, Ph); *m/z* (CI, NH_3_) 632 (M + NH_4_^+^), 615, 583, 490, 463, 461, 234 and 90 [Found: M + NH_4_^+^ 632.3440. C_33_H_54_O_7_NSi_2_ requires 632.3440].

**7-*O*-*tert*-Butyldiphenylsilyl-1,2-dideoxy-3,6-di-*O*-methoxymethyl-4,5-*O*-isopropylidene-d-*allo*-hept-1-ynitol (5a) and 7-*O*-*tert*-butyldiphenylsilyl-1,2-dideoxy-3,6-di-*O*-methoxymethyl-4,5-*O*-isopropylidene-d-*altro*-hept-1-ynitol (5b):** 7-*O*-*tert*-butyldiphenylsilyl-1,2-dideoxy-4,5-*O*-isopropylidene-d-hept-1-ynitols (**2c**, 0.994 g, 2.190 mmol) were dissolved in dry DCM (15 ml) and placed at 0 °C. *N,N*-Diisopropylethylamine (2.82 g, 3.8 ml, 21.8 mmol) was added, followed by chloromethylmethyl ether (1.8 g, 1.7 ml, 22.5 mmol) and after 20 min the reaction mixture was warmed to room temperature and left to stir for 48 h. The solvent was removed under reduced pressure, the residue was dissolved in ethyl acetate (100 ml), washed with water (70 ml) and extracted with ethyl acetate (2 x 100 ml). The organic layers were combined, dried (Na_2_SO_4_) and purified by flash chromatography on silica gel using light petroleum/diethyl ether (4:1) as eluent to yield two separable diastereoisomers **5a** (0.576 g, 49%) and **5b** (0.255 g, 21%) as colourless oils.

The most polar **5a**, [α]_D_ −5.4 (*c* 8.1 in CHCl_3_); δ_H_ (270 MHz) 1.07 (9 H, s, CMe_3_), 1.38 (3 H, s, Me), 1.54 (3 H, s, Me), 2.44 (1 H, d, *J* 1.98, C≡C-*H*), 3.38 (3 H, s, OMe), 3.39 (3 H, s, OMe), 3.85–3.90 (1 H, dd, *J* 3.3 and 11.21, C*H*HOSi), 3.99–4.04 (1 H, dd, *J* 1.98 and 11.21, CH*H*OSi), 4.11–4.15 (1 H, dt, *J* 2.64 and 8.57, C*H*CH_2_OSi), 4.42–4.45 (1 H, dd, *J* 2.64, 3.3 and 7.26, C*H*CHC≡), 4.54–4.60 (1 H, dd, *J* 7.26 and 8.57, C*H*CHCH_2_OSi), 4.63 (1 H, t, *J* 3.3, C*H*C≡), 4.74 (2 H, d, *J* 7.25, OC*H_2_*O), 4.84 (1 H, d, *J* 7.26, OC*H*HO), 4.99 (1 H, d, *J* 6.59, OCH*H*O), 7.34–7.42 (6 H, m, *meta-* and *para-*Ph), 7.64–7.71 (4 H, m, *ortho-*Ph); δ_C_ (67.8 MHz) 19.29 (1 C, *C*Me_3_), 24.83 (1 C, Me), 26.55 (1 C, Me), 26.82 (3 C, C*Me_3_*), 55.83 and 56.24 (2 C, 2 x OMe), 63.44 (1 C, CH_2_OSi), 65.26 (1 C, C≡*C*-H), 74.34, 75.96, 76.22 and 76.43 (4 C, 4 x CHOR), 78.60(1 C, *C*≡C-H), 93.95 and 96.21 (2 C, 2 x O*C*H_2_O), 108.99 (1 C, *C*Me_2_), 127.58 (2 C, Ph), 127.65 (2 C, Ph), 129.62 (1 C, Ph), 129.67 (1 C, Ph), 129.78 (1 C, quat. Ph), 133.55 (1 C, quat. Ph), 135.68 (2 C, Ph), 135.74 (2 C, Ph); *m/z* (CI, NH_3_) 560 (M + NH_4_^+^), 511, 553, 389, 331, 78, 58 and 52. [Found M + NH_4_^+^ 560.3044. C_30_H_46_NO_7_Si requires 560.3044].

The less polar **5b**, δ_H_ (270 MHz) 1.06 (9 H, s, CMe_3_), 1.36 (3 H, s, Me), 1.43 (3 H, s, Me), 2.48 (1 H, d, *J* 1.98, C≡C-*H*), 3.37 (3 H, s, OMe), 3.38 (3 H, s, OMe), 3.86–3.92 (1 H, dd, *J* 3.95 and 11.22, C*H*HOSi), 3.96–4.01 (1 H, dd, *J* 1.98, 2.64 and 11.87, CH*H*OSi), 4.08–4.12 (1 H, ddd, *J* 1.98, 4.62 and 7.26, C*H*CH_2_OSi), 4.37–4.40 (1 H, dd, *J* 4.62 and 5.93, C*H*CHC≡), 4.45–4.50 (1 H, t, *J* 7.25, C*H*CHCH_2_OSi), 4.61 (1 H, dd, *J* 1.98, 2.64 and 4.62, C*H*C≡), 4.65 (1 H, d, *J* 6.59, OC*H*HO), 4.73 (1 H, d, *J* 6.59, OCH*H*O), 4.86 (1 H, d, *J* 6.59, OC*H*HO), 4.96 (1 H, d, *J* 6.6, OCH*H*O), 7.30–7.44 (6 H, m, *para-* and *meta-*Ph) and 7.64–7.77 (4 H, m, *ortho-*Ph); δ_C_ (67.8 MHz) 19.26 (1 C, *C*Me_3_), 25.31 (1 C, Me), 26.49 (1 C, Me), 26.81 (3 C, C*Me_3_*), 55.98 and 56.17 (2 C, 2 x OMe), 64.15 (1 C, *C*H_2_OSi), 65.20 (1 C, C≡*C*-H), 75.09, 75.55, 76.76 and 78.94 (4 C, 4 x CHOR), 80.27 (1 C, *C*≡C-H), 94.58 and 96.46 (2 C, 2 x O*C*H_2_O), 109.22 (1 C, *C*Me_2_), 127.59 (2 C, Ph), 127.65 (2 C, Ph), 129.61 (2 C, Ph), 133.46 (1 C, quat. Ph), 133.58 (1 C, quat. Ph), 135.68 (2 C, Ph) and 135.74 (2 C, Ph); *m/z* (CI, NH_3_) 560 (M + NH_4_^+^), 511, 553, 389, 331, 78, 58 and 52. [Found M + NH_4_^+^ 560.3044. C_30_H_46_NO_7_Si requires 560.3044].

**1,2-Dideoxy-3,6-di-*O*-methoxymethyl-4,5-*O*-isopropylidene-1-phenyl-d-*allo*-hept-1-ynitol (6a):** 7-*O*-*tert*-Butyldiphenylsilyl-1,2-dideoxy-3,6-di-*O*-methoxymethyl-4,5-*O*-isopropylidene-1-phenyl-d-*allo*-hept-1-ynitol (**3a**, 1.120 g, 1.813 mmol) was dissolved in dry THF (20 ml), to this was added TBAF (1 M in THF, 5.4 ml, 5.4 mmol). After 12 h, the solvent was evaporated, the residue was diluted in ethyl acetate (100 ml), washed with water (50 ml) and extracted with ethyl acetate (2 x 100 ml), the organic layers were combined, dried (Na_2_SO_4_) and concentrated. The residue was purified by flash chromatography on silica gel using light petroleum/diethyl ether (1:3) as eluent to afford the title compound **6a** (0.6 g, 87%) as a colourless oil, [α]_D_ +125 (*c* 0.27 in CHCl_3_); ν_max_(film)/cm^-1^ 3467 (OH), 2985, 2937, 2894, 2825, 2229 (C≡C), 1598, 1490, 1444, 1378, 1255, 1214, 1149, 1099, 1035, 917; δ_H_ (270 MHz) 1.40 (3 H, s, Me), 1.53 (3 H, s, Me), 3.30–3.35 (1 H, dd, *J* 5.27 and 8.57, CH_2_O*H*), 3.43 (3 H, s, OMe), 3.46 (3 H, s, OMe), 3.63–3.72 (1 H, dt, *J* 5.28 and 12.21, C*H*HOH), 3.90–3.94 (1 H, ddd, *J* 2.64 and 8.58, CH*H*OH), 4.13–4.19 (1 H, dq, *J* 2.64 and 8.91, C*H*CH_2_OH), 4.25–4.31 (1 H, dd, *J* 6.92 and 8.91, CHOR), 4.48–4.51 (1 H, dd, *J* 2.64 and 6.93, CHOR), 4.70 (1 H, d, *J* 6.93, OC*H*HO), 4.80 (2 H, ab. q, *J* 7.26, OC*H_2_*O), 4.87 (1 H, d, *J* 2.64, C*H*C≡), 5.10 (1 H, d, *J* 6.93, OCH*H*O), 7.31–7.33 (3 H, m, *meta-* and *para-*Ph), 7.42–7.46 (2 H, m, *ortho-*Ph); δ_C_ (67.8 MHz) 24.97 (1 C, Me), 26.80 (1 C, Me), 55.80 and 56.02 (2 C, 2 x OMe), 63.97(1 C, CH_2_), 65.34, 75.23, 78.88 and 79.98 (4 C, 4 x CHOR), 84.73 and 87.54 (2 C, C≡C), 93.83 and 97.07 (2 C, 2 x OCH_2_O), 109.31 (1 C, *C*Me_2_), 122.21 (1 C, Ph), 128.27 (2 C, Ph), 128.53 (1 C, Ph), 131.58 (2 C, Ph); *m/z* (CI, NH_3_) 398 (M + NH_4_^+^), 381 (M^+^+ H), 320, 319 (M^+^ − OMOM), 287 and 261 [Found: M + NH_4_^+^ 398.2179. C_20_H_32_O_7_N requires 398.2179].

**1,2-Dideoxy-3,6-di-*O*-methoxymethyl-4,5-*O*-isopropylidene-1-phenyl-d-*altro*-hept-1-ynitol (6b):** 7**-***O*-*tert*-Butyldiphenylsilyl-1,2-dideoxy-3,6-di-*O*-methoxymethyl-4,5-*O*-isopropylidene-1-phenyl-d-*altro*-hept-1-ynitol (**3b**, 0.778 g, 1.26 mmol) was dissolved in dry THF (15 ml). TBAF (1 M in THF, 3.8 ml, 3.75 mmol) was added and this solution was stirred for 16 h at room temperature. The solvent was evaporated and the residue diluted in ethyl acetate (100 ml), washed with water (50 ml) and extracted with ethyl acetate (2 x 100 ml). The organic layers were combined, dried over Na_2_SO_4_ and concentrated to be purified by flash chromatography on silica gel using light petroleum/diethyl ether (1:1) as eluent to yield the title compound **6b** (0.437 g, 92%) as a colourless oil, [α]_D_ −52 (*c* 0.12 in CHCl_3_); ν_max_(film)/cm^-1^ 3463 (OH), 3058, 2985, 2937, 2892, 2825, 2225 (C≡C), 1298, 1490, 1444, 1380, 1253, 1216, 1151, 1079, 1033, 917; δ_H_ (270 MHz) 1.40 (3 H, s, Me), 1.53 (3 H, s, Me), 3.24–3.29 (1 H, dd, *J* 4.62 and 8.91, CH_2_O*H*), 3.43 (3 H, s, OMe), 3.45 (3 H, s, OMe), 3.72–3.78 (1 H, ddd, *J* 4.62, 10.89 and 16.83, C*H*HOH), 3.87–3.92 (1 H, ddd, *J* 2.97 and 8.91, CH*H*OH), 4.16–4.21 (1 H, td, *J* 2.97 and 6.27, C*H*CH_2_OH), 4.30–4.35 (1 H, t, *J* 5.94, CHOR), 4.45–4.50 (1 H, t, *J* 5.94, CHOR), 4.70–4.78 (4 H, dd and d, *J* 2.31 and 6.93, C*H*C≡, OC*H_2_*O, OC*H*HO), 5.06 (1 H, d, *J* 6.59, OCH*H*O), 7.26–7.33 (3 H, m, *meta-* and *para-*Ph), 7.43–7.46 (2 H, m, *ortho-*Ph); δ_C_ (67.8 MHz) 25.24 (1 C, Me), 26.85 (1 C, Me), 55.99 and 55.85 (2 C, 2 x OMe), 63.84 (1 C, CH_2_), 65.69, 77.11, 78.67 and 79.89 (4 C, 4 x CHOR), 85.01 and 87.42 (2 C, C≡C), 94.36 and 97.26 (2 C, 2 x O*C*H_2_O), 109.26 (1 C, *C*Me_2_), 122.23 (1 C, Ph), 128.27 (2 C, Ph), 128.63 (1 C, Ph), 131.73 (2 C, Ph); *m/z* (CI, NH_3_) 398 (M + NH_4_^+^), 381 (M^+^+ H), 319 (M^+^ −OMOM), 287 and 261 [Found: M + NH_4_^+^ 398.2179. C_20_H_32_O_7_N requires 398.2179].

**7-Iodo-1,2,7-trideoxy-3,6-di-*O*-methoxymethyl-4,5-*O*-isopropylidene-1-phenyl-d-*allo*-hept-1-ynitol (7a):** To a solution of 1,2-dideoxy-3,6-di-*O*-methoxymethyl-4,5-*O*-isopropylidene-1-phenyl-d-*allo*-hept-1-ynitol (**6a**, 0.387 g, 1.015 mmol) in dry toluene (10 ml), triphenylphosphine (0.8 g, 3.046 mmol) and imidazole (0.21 g, 3.046 mmol) were added under vigorous stirring. The mixture was placed under reflux and iodine (0.56 g, 2.234 mmol) was added in small portions. After 2 h, the reaction mixture was cooled to room temperature and poured onto a saturated solution of sodium thiosulphate (20 ml) and extracted with ethyl acetate (3 x 50 ml). The organic layers were combined, dried over Na_2_SO_4_ and evaporated under reduced pressure. The residue was purified by flash chromatography on silica gel using light petroleum/diethyl ether (4:1) as eluent to afford the title compound **7a** (0.429 g, 86%) as a colourless oil, [α]_D_ +87 (*c* 0.21 in CHCl_3_); ν_max_(film)/cm^-1^ 3054, 2985, 2892, 2823, 2782, 2229 (C≡C), 1598, 1490, 1442, 1378, 1255, 1214, 1151, 1095, 1031; δ_H_ (270 MHz) 1.42 (3 H, s, Me), 1.57 (3 H, s, Me), 3.42 (3 H, s, OMe), 3.52 (3 H, s, OMe), 3.56–3.61 (1 H, m, C*H*CH_2_I), 3.68–3.78 (2 H, m, CH_2_I), 4.31–4.37 (1 H, t, *J* 7.92 and 6.6, C*H*CHCH_2_I), 4.51 (1 H, dd, *J* 2.0, 2.63 and 7.25, C*H*CHC≡), 4.68 (1 H, d, *J* 7.26, OC*H*HO), 4.76–4.82 (2 H, t, *J* 7.26, OCH_2_O), 4.87 (1 H, d, *J* 2.64, C*H*C≡), 5.09 (1 H, d, *J* 7.26, OCH*H*O), 7.29–7.34 (3 H, m, *meta-* and *para-* Ph), 7.43 (2 H, m, *ortho-*Ph); δ_C_ (67.8 MHz) 12.06 (1 , *C*H_2_I), 24.77 (1 , Me), 26.73 (1 , Me), 55.80 and 57.42 (2 , 2 x OMe), 65.63, 73.67, 77.47 and 78.54 (4 , 4 x CHOR), 84.89 and 87.79 (2 C, C≡C), 93.93 and 96.78 (2 C, 2 x OCH2O), 109.30 (1 C, *C*Me_2_), 122.36 (1 C, Ph), 128.37 (2 C, Ph), 128.66 (1 C, Ph), 131.72 (2 C, Ph); *m/z* (CI, NH_3_) 508 (M + NH_4_^+^), 461, 429. [Found: M + NH_4_^+^ 508.1196. C_20_H_31_O_6_NI requires 508.1196].

**7-Iodo-1,2,7-trideoxy-3,6-di-*O*-methoxymethyl-4,5-*O*-isopropylidene-1-phenyl-d-*altro*-hept-1-ynitol (7b):** To a solution of 1,2-dideoxy-3,6-di-*O*-methoxymethyl-4,5-*O*-isopropylidene-1-phenyl-d-*altro*-hept-1-ynitol (**6b**, 0.338 g, 0.886 mmol) in dry toluene (10 ml), triphenylphosphine (0.71 g, 2.657 mmol) and imidazole (0.20 g, 2.657 mmol) were added under vigorous stirring. The mixture was placed under reflux. Iodine (0.5 g, 1.95 mmol) was added in small portions. After 2 h, the reaction mixture was cooled and washed with a saturated solution of sodium thiosulphate (20 ml) and extracted with ethyl acetate (3 x 50 ml). The organic layers were combined, dried over Na_2_SO_4_, filtered and evaporated under reduced pressure, the residue was purified by flash chromatography on silica gel using light petroleum/diethyl ether (4:1) as eluent to afford the title compound **7b** (0.329 g, 76%) as a colourless oil, [α]_D_ −57 (*c* 0.32 in CHCl_3_); ν_max_(film)/cm^-1^ 3056, 2985, 2938, 2892, 2825, 2782, 2225, 1598, 1490, 1371, 1249, 1216, 1151, 1078, 1060 and 919; δ_H_ (270 MHz) 1.42 (3 H, s, Me), 1.53 (3 H, s, Me), 3.44 (3 H, s, OMe), 3.50 (3 H, s, OMe), 3.58–3.62 (1 H, dd, *J* 3.29 and 10.55, C*H*HI), 3.65–3.70 (1 H, dd, *J* 2.97 and 13.86, CH*H*I), 3.8 (1 H, dt, *J* 2.97 and 7.26, C*H*CH_2_I), 4.31–4.36 (1 H, t, *J* 5.94, CHOR), 4.44 (1 H, t, *J* 5.61, CHOR), 4.69–4.72 (3 H, m, *J* 5.61, C*H*C≡ and OC*H_2_*O), 4.83 (1 H, d, *J* 7.26, OC*H*HO), 5.10 (1 H, d, *J* 6.6, OCH*H*O), 7.31 (3 H, m, *meta-* and *para-*Ph), 7.45 (2 H, m, *ortho-*Ph); δ_C_ (67.8 MHz) 11.61 (1 C, CH_2_I), 25.11 (1 C, Me), 26.88 (1 C, Me), 56.01 and 57.12 (2 C, 2 x OMe), 66.01, 74.52, 78.24 and 78.42 (4 C, 4 x CHOR), 85.12 and 87.50 (2 C, C≡C), 94.30 and 97.34 (2 C, 2 x O*C*H_2_O), 109.13 (1 C, *C*Me_2_), 122.32 (1 C, Ph), 128.32 (2 C, Ph), 128.66 (1 C, Ph), 131.76 (2 C, Ph); *m/z* (CI, NH_3_) 508 (M + NH_4_^+^), 303, 132. [Found M + NH_4_^+^, 508.1196. C_20_H_31_O_6_NI requires 508.1196].

**1,2-Dideoxy-3,6-di-*O*-methoxymethyl-4,5-*O*-isopropylidene-d-*allo*-hept-1-ynitol (8a):** 7-*O*-*tert*-Butyldiphenylsilyl-3,6-di-*O*-methoxymethyl-4,5-*O*-isopropylidene-1-trimethylsilyl-d-*allo*-hept-1-ynitol (**4a**, 0.793 g, 1.284 mmol) was dissolved in dry THF (20 ml) at room temperature. TBAF (1 M in THF, 6.4 ml, 6.42 mmol) was added. After 12 h, the reaction mixture was concentrated, the residue was dissolved in ethyl acetate (50 ml), washed with water (25 ml) and extracted with ethyl acetate (2 x 50 ml), the organic layers were combined, dried over Na_2_SO_4_, and purified by flash chromatography on silica gel with light petroleum/diethyl ether (1:3) as eluent to afford the title compound **8a** (0.373 g, 96%) as a colourless oil, [α]_D_ +108 (*c* 3.42 in CHCl_3_); ν_max_(film)/cm^-1^ 3467 (OH), 3262 (C≡C-H), 2985, 2938, 2896, 2827, 2113 (C≡C), 1457, 1376, 1257, 1214, 1149, 1079, 1037, 917, 890, 844, 755; δ_H_ (270 MHz) 1.38 (3 H, s, Me), 1.55 (3 H, s, Me), 2.52 (1 H, d, *J* 1.98, C≡C-H), 3.33–3.38 (1 H, m, OH), 3.40 (3 H, s, OMe), 3.46 (3 H, s, OMe), 3.62–3.70 (1 H, m, C*H*HOH), 3.88–3.97 (1 H, qd, *J* 2.64, 8.58 and 11.22, CH*H*OH), 4.06–4.12 (1 H, ddd, *J* 2.64, 5.28 and 8.58, C*H*CH_2_OH), 4.20–4.26 (1 H, dd, *J* 6.6 and 8.58, CHOR), 4.38–4.41 (1 H, dd, *J* 2.64 and 6.59, CHOR), 4.65 (2 H, m, *J* 6.6, C*H*C≡ and OC*H*HO), 4.73–4.81 (2 H, dd, *J* 6.6 and 12.54, OC*H_2_*O), 5.00 (1 H, d, *J* 6.6, OCH*H*O); δ_C_ (67.8 MHz) 24.97 (1 C, Me), 26.81 (1 C, Me), 56.00 and 56.11 (2 C, 2 x OMe), 64.00 (1 C, *C*H_2_OH), 64.91 (1 C, C≡*C*-H), 75.43, 76.18, 78.59 and 79.63 (4 C, 4 x CHOR), 79.81 (1 C, *C*≡C-H), 94.04 and 97.19 (2 C, 2 x O*C*H_2_O) and 109.30 (1 C, *C*Me_2_); *m/z* (CI, NH_3_) 322 (M^+^+ NH_4_^+^), 290 (M^+^ − Me), 273 (-HO), 246, 241, 229, 215 and 204. [Found: MH^+^ 305.1601. C_14_H_25_O_7_ requires MH, 305.1600].

**1,2-Dideoxy-3,6-di-*O*-methoxymethyl-4,5-*O*-isopropylidene-d-*altro*-hept-1-ynitol (8b):** 7-*O*-*tert*-Butyldiphenylsilyl-1,2-dideoxy-3,6-di-*O*-methoxymethyl-4,5-*O*-isopropylidene-1-trimethylsilyl-d-*altro*-hept-1-ynitol (**4b**, 0.241 g, 0.39 mmol) was dissolved in dry THF (15 ml) at room temperature. TBAF (1 M in THF, 1.9 ml, 1.95 mmol) was added. After 12 h, the reaction mixture was washed with water (10 ml) and extracted with ethyl acetate (3 x 50 ml). The organic layers were combined, dried over Na_2_SO_4_, concentrated and the residue was purified by flash chromatography on silica gel using light petroleum/diethyl ether (1:3) as eluent to yield the title compound **8b** (0.108 g, 91%) as a colourless oil, [α]_D_ −48.7 (*c* 0.66 in CHCl_3_); ν_max_(film)/cm^-1^ 3463 (OH), 3262 (C≡C-H), 2985, 2938, 2896, 2827, 2113 (C≡C), 1461, 1376, 1249, 1218, 1153, 1079, 1033, 917, 883, 740; δ_H_ (270 MHz) 1.38 (3 H, s, Me), 1.51 (3 H, s, Me), 1.71 (1 H, bs, OH), 2.52 (1 H, d, *J* 2.14, C≡C-H), 3.43 (3 H, s, OMe), 3.46 (3 H, s, OMe), 3.65–3.73 (1 H, ddd, *J* 4.62, 6.02 and 12.29, C*H*HOH), 3.85–3.94 (1 H, ddd, *J* 2.81, 8.74 and 11.79, CH*H*OH), 4.01–4.08 (1 H, ddd, *J* 2.8, 6.27 and 7.92, C*H*CH_2_OH), 4.23–4.28 (1 H, dd, *J* 6.1 and 7.84, CHOR), 4.38 (1 H, t, *J* 6.02, CHOR), 4.52 (1 H, dd, *J* 2.14 and 5.28, C*H*C≡), 4.67–4.78 (3 H, m, OC*H_2_*O, OC*H*HO), 5.00 (1 H, d, *J* 6.76, OCH*H*O); δ_C_ (67.8 MHz) 25.26 (1 C, Me), 26.71 (1 C, Me), 55.97 and 56.19 (2 C, 2 x OMe), 63.84 (1 C, *C*H_2_OH), 64.93 (1 C, C≡*C*-H), 75.46, 76.43, 76.93, 78.65 (4 C, 4 x CHOR), 79.97 (1 C, *C*≡C-H), 94.39 and 97.11 (2 C, 2 x OCH_2_O), 109.48 (1 C, *C*Me_2_); *m/z* (EI) 305 (M^+^ + H^+^), 290 (M^+^−Me), 273 (-HO), 241, 229, 215, 186, 109 and 58. [Found: MH^+^ 305.1600. C_14_H_25_O_7_ requires 305.1600].

**7-Iodo-1,2,7-trideoxy-3,6-di-*O*-methoxymethyl-4,5-*O*-isopropylidene-d-*allo*-hept-1-ynitol (9a):** A solution of 1,2-dideoxy-3,6-di-*O*-methoxymethyl-4,5-*O*-isopropylidene-d-*allo*-hept-1-ynitol (**8a**, 0.267 g, 0.882 mmol) in dry toluene (20 ml) was placed under reflux. Triphenylphosphine (0.7 g, 2.645 mmol) and imidazole (0.2 g, 2.645 mmol) were added under vigorous stirring. When the temperature of reflux was reached, iodine (0.5 g, 1.94 mmol) was added. After 2 h, the reaction mixture was cooled, poured onto a saturated solution of sodium thiosulphate (30 ml) and the residue was extracted with ethyl acetate (3 x 40 ml). The organic layers were combined, dried (Na_2_SO_4_), concentrated and purified by flash chromatography on silica gel using light petroleum/diethyl ether (3:1) to afford the title compound **9a** (0.267g, 73%) as a colourless oil, [α]_D_ +35 (*c* 16.3 in CHCl_3_); ν_max_(film)/cm^-1^ 3270 (C≡C-H), 2985, 2938, 2896, 2827, 2117 (C≡C), 1446, 1376, 1257, 1214, 1153, 1095, 1045, 917, 890 and 848; δ_H_ (270 MHz) 1.39 (3 H, s, Me), 1.54 (3 H, s, Me), 2.59 (1 H, d, *J* 2.64, C≡C-H), 3.38 (3 H, s, OMe), 3.50 (3 H, s, OMe), 3.54–3.62 (2 H, m, C*H_2_*I), 3.66–3.72 (1 H, m, C*H*CH_2_I), 4.25–4.31 (1 H, t, *J* 6.59, CHOR), 4.38–4.42 (1 H, dd, *J* 2.64 and 6.59, CHOR), 4.64 (2 H, m, OC*H*HO and C*H*C≡), 4.72–4.78 (2 H, dd, *J* 7.25 and 9.89, OC*H_2_*O), 4.98 (1 H, d, *J* 6.59, OCH*H*O); δ_C_ (67.8 MHz) 11.46 (1 C, CH_2_I), 24.35 (1 C, Me), 26.26 (1 C, Me), 55.43 and 56.98 (2 C, 2 x OMe), 64.52 (1 C, C≡*C*-H), 72.91, 76.49, 77.44 and 77.70 (4 C, 4 x CHOR), 79.19 (1 C, *C*≡C-H), 93.45 and 96.24 (2 C, 2 x O*C*H_2_O), 108.67 (1 C, *C*Me_2_); *m/z* (CI, NH_3_) 432 (M^+^ + NH_3_), 415 (M^+^ + H), 383, 306, 244, 213 and 183. [Found: M + H^+^ 415.0617. C_14_H_24_O_6_I requires 415.0617].

**7-Iodo-1,2,7-trideoxy-3,6-di-*O*-methoxymethyl-4,5-*O*-isopropylidene-d-*altro*-hept-1-ynitol (9b):** Triphenylphosphine (0.13 g, 0.711 mmol) and imidazole (0.075 g, 0.711 mmol) were added to a vigorously stirred solution of 1,2-dideoxy-3,6-di-*O*-methoxymethyl-4,5-*O*-isopropylidene-d-*altro*-hept-1-ynitol (**8b**, 0.072 g, 0.237 mmol) in dry toluene (10 ml), placed under reflux and iodine (0.13 g, 0.521 mmol) was added. After 2 h, the reaction mixture was directly poured onto a saturated solution of sodium thiosulphate (30 ml) and extracted with ethyl acetate (3 x 40 ml). The organic layers were combined, dried over Na_2_SO_4_, concentrated and purified by flash chromatography on silica gel using light petroleum/diethyl ether (3:1) to afford the title compound **9b** (0.076 g, 78%) as a white crystalline solid, mp 58–59 °C, [α]_D_ −49 (*c* 0.66 in CHCl_3_); ν_max_(film)/cm^-1^ 3274 (C≡C-H), 2985, 2935, 2892, 2827, 2117 (C≡C), 1461, 1403, 1376, 1249, 1218, 1153, 1091, 1041, 917, 887 and 860; δ_H_ (270 MHz) 1.36 (3 H, s, Me), 1.46 (3 H, s, Me), 2.50 (1 H, d, *J* 1.97, C≡C-*H*), 3.38 (3 H, s, OMe), 3.46 (3 H, s, OMe), 3.50–3.64 (3 H, m, C*H_2_*I and C*H*CH_2_I), 4.20–4.25 (1 H, bt, *J* 5.93, CHOR), 4.30–4.34 (1 H, t, *J* 5.94, CHOR), 4.49 (1 H, dd, *J* 1.98, 2.63 and 4.61, C*H*C≡), 4.64 (1 H, d, *J* 6.6, OC*H*HO), 4.67–4.76 (2 H, q, *J* 7.25, OC*H_2_*O), 4.94 (1 H, d, *J* 6.59, OCH*H*O); δ_C_ (67.8 MHz) 11.38 (1 C, CH_2_I), 25.06 (1 C, Me), 26.64 (1 C, Me), 56.08 and 57.08 (2 C, 2 x OMe), 65.20 (1 C, C≡*C*-H), 73.91, 75.49, 77.99 and 78.18 (4 C, 4 x CHOR), 79.77 (1 C, *C*≡C-H), 94.33 and 96.84 (2 C, 2 x O*C*H_2_O), 109.17 (1 C, *C*Me_2_); *m/z* (CI, NH_3_) 432 (M^+^ +NH_3_), 415 (M^+^ +H), 307, 295, 279, 246, 227, 197, 188 and 170 [Found: M + NH_4_^+^ 414.0883. C_14_H_27_O_6_NI requires 432.0883]; (Found: C, 40.71; H, 5.68. C_14_H_23_O_6_I requires C, 40.59; H, 5.60%).

**(2*S*,3*S*,4*R*,5*R*)-1-Benzylidene-3,4-(isopropylidenedioxy)-2,5-bis(methoxymethoxy)cyclohexane (10):** 7-Iodo-1,2,7-trideoxy-3,6-di-*O*-methoxymethyl-4,5-*O*-isopropylidene-1-phenyl-d-*allo*-hept-1-ynitol (**7a**, 0.353 g, 0.717 mmol) was dissolved in dry benzene (15 ml) and the solution was degassed with argon for 1 h at room temperature. At the same time, a solution of AIBN (0.012 g, 0.07 mmol) in dry benzene (5 ml) was also degassed with argon at room temperature. The solution of the iodo compound **7a** was placed under a vigorous reflux (80 °C). Tributyltin hydride (0.5 equiv, 0.1ml, 0.359 mmol) was added to the solution of AIBN which was added to the solution of the iodo compound **7a** *via* a double-tipped needle. After 15 min under reflux, another portion of tributyltin hydride (0.12 ml, 0.446 mmol) in benzene (10 ml) was added to the reaction mixture. After 1 h, the solution was cooled to room temperature, washed with petroleum (50 ml) and extracted with acetonitrile (2 x 100 ml). The acetonitrile layers were combined, concentrated and purified by flash chromatography on silica gel using light petroleum/diethyl ether (3:1) as eluent and filtered through silica gel with diethyl ether to yield the title compound **10** as a mixture of two inseparable isomers, **10** (0.242 g, 93%) which is a colourless oil, [α]_D_ −172 (*c* 6.7 in CHCl_3_); ν_max_(film)/cm^-1^ 2985, 2935, 2846, 1735, 1600 (C=C), 1450, 1376, 1261, 1214, 1149, 1103, 987, 917 and 867; δ_H_ (270 MHz) (mixture of isomers, *E*:*Z* as 1:1.6) 1.36 (1.73 H, s, major Me), 1.38 (1.27 H, s, minor Me), 1.40 (1.73 H, s, major Me), 1.47 (1.27 H, s, minor Me), 2.61–2.77 (0.44 H, tdd, *J* 1.31, 1.98, 9.89 and 25.72, minor CH_2_), 2.79–2.94 (1.56 H, td, *J* 1.98, 7.91 and 23.75, major CH_2_), 3.21 (1.1 H, s, major OMe), 3.39 and 3.42 (3 H, 2s, minor 2 x OMe), 3.43 (1.9 H, s, major OMe), 4.14 (0.6 H, d, *J* 3.3, major C*H*CH_2_), 4.34–4.43 (1.38 H, qd, *J* 3.3, 5.94 and 12.54, minor C*H*CH_2_ and major C*H*CHCH_2_), 4.43 (1.54 H, m, *J* 2.64, 3.3 and 5.94, major C*H*CHC= and minor C*H*CHC=, minor C*H*CHCH_2_), 4.57 (0.9 H, m, 2 x minor OC*H_2_*O), 4.61 (0.7 H, dd, *J* 1.98, 2.64 and 7.26, major C*H*C=), 4.70–4.73 (0.53 H, dd, *J* 3.3, 2.63 and 5.94, 5.27, minor C*H*C=), 4.74 (2.58 H, m, 2 x major OC*H_2_*O), 6.50 (0.63 H, t, *J* 1.98 and 2.63, major C=C*H*Ph), 6.72 (0.39 H, t, J 1.98, minor C=C*H*Ph), 7.20–7.36 (5.22 H, m, Ph); δ_C_ (67.8 MHz)(major, *Z* isomer) 24.57 (1 C, Me), 26.33 (1 C, Me), 28.38 (1 C, CH_2_), 55.30 (2 C, 2 x OMe), 70.98, 74.95, 76.53 and 78.47 (4 C, 4 x CHOR), 93.19 and 93.90 (2 C, 2 x O*C*H_2_O), 109.57 (1 C, *C*Me_2_), 126.86 (2 C, Ph), 128.13 and 128.54 (2 C, C=C), 130.83 (2 C, Ph), 132.12 (1 C, Ph), 136.56 (1 C, Ph); (minor, *E* isomer) 24.36 (1 C, Me), 26.16 (1 C, Me), 31.12 (1 C, CH_2_), 55.30 (2 C, 2 x OMe), 70.61, 75.12, 77.20, 77.47 (4 C, 4 x CHOR), 95.59 (2 C, 2 x O*C*H_2_O), 109.68 (1 C, *C*Me_2_), 126.61 (2 C, Ph), 128.00 and 128.75 (2 C, C=C), 131.79 (2 C, Ph), 132.86 (1 C, Ph), 136.49 (1 C, Ph); *m/z* (EI) 365 (MH^+^), 333, 303, 262. [Found MH^+^ 365.1964. C_20_H_29_O_6_ requires 365.1964].

**(1*Z*,2*R*,3*S*,4*R*,5*R*)-1-Benzylidene-3,4-(isopropylidenedioxy)-2,5-bis(methoxymethoxy)­cyclohexane (11) and (1*E*,2*R*,3*S*,4*R*,5*R*)-1-benzylidene-3,4-(isopropylidenedioxy)-2,5-bis­(methoxymethoxy)­cyclohexane (12):** 7-Iodo-1,2,7-trideoxy-3,6-di-*O*-methoxymethyl-4,5-*O*-isopropylidene-1-phenyl-d-*altro*-hept-1-ynitol (**7b**, 0.338 g, 0.774 mmol) was dissolved in dry benzene (10 ml) and degassed with argon for 1 h and placed under reflux. At the same time, a solution of AIBN (0.013 g, 0.077mmol) in dry benzene (5 ml) was also degassed with argon. Tributyltin hydride (0.3 ml, 1.0 mmol) was added to the solution of AIBN which was added to the solution of the iodo compound **7b** *via* a double-tipped needle. After 5 h, another solution of AIBN in benzene was added and after 30 min, the reaction was finished and allowed to cool to room temperature. The mixture was washed with petroleum (30 ml) and extracted with acetonitrile (3 x 50 ml). The acetonitrile layers were combined and concentrated to be purified by flash chromatography on silica gel using light petroleum/diethyl ether (2:1) as eluent to yield the two separable isomers **11** (0.094 g, 34%) and **12** (0.076 g, 27%), both as colourless oils.

*R_f_* = 0.49 in light petroleum/diethyl ether (2:1) **11**, [α]_D_ −31 (*c* 2.79 in CHCl_3_); ν_max(_film)/cm^-1^ 2923, 1727, 1600 (C=C), 1461, 1376, 1249, 1214, 1149, 1106, 1033, 991, 917; δ_H_ (270 MHz) 1.42 (3 H, s, Me), 1.64 (3 H, s, Me), 2.36–2.43 (1 H, dd, *J* 5.27, 5.94 and 12.53, C*H*HC=C), 2.88–2.97 (1 H, td, *J* 1.32, 11.88 and 12.53, CH*H*C=C), 3.25 (3 H, s, OMe), 3.45 (3 H, s, OMe), 3.84–3.92 (1 H, dt, *J* 5.28, 4.61 and 11.87, CH_2_C*H*OMOM), 4.20 (1 H, t, *J* 5.28, C*H*CHCH_2_), 4.40 (1 H, d, *J* 7.25, OC*H*HO), 4.49 (1 H, d, *J* 6.6, OCH*H*O), 4.54 (1 H, t, *J* 5.28 and 4.61, C*H*CHC=), 4.61 (1 H, d, *J* 5.28, C*H*C=), 4.78–4.86 (2 H, dd ab, *J* 6.59 and 7.26, OC*H_2_*O), 6.60 (1 H, s, C=C*H*Ph), 7.19–7.32 (5 H, m, Ph); δ_C_ (67.8 MHz) 25.54 (1 C, Me), 25.65 (1 C, Me), 32.30 (1 C, CH_2_), 55.30 and 55.55 (2 C, 2 x OMe), 69.73, 73.76, 74.86 and 76.04 (4 C, 4x CHOR), 94.34 and 95.49 (2 C, 2 x O*C*H_2_O), 110.23 (1 C, *C*Me_2_), 126.99 (2 C, Ph), 128.10 and 128.42 (2 C, C=C), 130.86 (2 C, Ph), 134.34 (1 C, Ph) and 136.25 (1 C, Ph); *m/z* (CI, NH_3_) 382 (M + NH_4_^+^), 365 (MH^+^), 320, 303 and 264. [Found MH^+^ 365.1962. C_20_H_29_O_6_ requires 365.1964].

*R_f_* = 0.26 in light petroleum/diethyl ether (2:1) **12**, [α]_D_ −70.5 (*c* 2.10 in CHCl_3_); ν_max_(film)/cm^-1^ 3054, 2927, 1727, 1658, 1600 (C=C), 1492, 1454, 1376, 1214, 1149, 1106, 1037, 987, 956, 917 and 871; δ_H_ (270 MHz) 1.26 (3 H, s, Me), 1.43 (3 H, s, Me), 2.91 (2 H, dt, *J* 9.23 and 1.31, CH_2_), 3.41 (3 H, s, OMe), 3.47 (3 H, s, OMe), 4.00–4.09 (1 H, td, *J* 1.98, 2.64 and 9.89, MOMOC*H*CH_2_), 4.36–4.42 (1 H, m, *J* 2.64, 4.62 and 9.89, C*H*CHOMOMCH_2_), 4.57 (1 H, d, *J* 2.64, C*H*CHOMOMC=), 4.61 (1 H, t, *J* 3.3, OC*H*HO), 4.64–4.68 (1 H, dd, *J* 1.98, 2.64 and 7.91, C*H*OMOMC=), 4.73 (1 H, s, OCH*H*O), 4.76–4.83 (2 H, dd ab, *J* 3.3, 6.6 and 11.87, OCH_2_O), 6.60 (1 H, dd, *J* 2.64 and 4.62, C=C*H*Ph), 7.13–7.54 (5 H, m, Ph); δ_C_ (67.8 MHz) 24.32 (1 C, Me), 25.80 (1 C, Me), 26.63 (1 C, CH_2_), 55.48 and 55.78 (2 C, 2 x OMe), 69.72, 73.94, 75.22 and 75.47 (4 C, 4 x CHOR), 95.17 and 95.50 (2 C, 2 x O*C*H_2_O), 110.03 (1 C, *C*Me_2_), 124.46 (2 C, Ph), 126.41 (2 C, Ph), 128.17 and 128.69 (2 C, C=C), 131.84 (1 C, Ph) and 137.26 (1 C, Ph); *m/z* (CI, NH_3_) 382 (M + NH_4_^+^), 365 (MH^+^), 320, 303 and 264. [Found MH^+^ 365.1960. C_20_H_29_O_6_ requires 365.1964].

**(2*R*,3*S*,4*R*,5*R*)-1-Methylidene-3,4-(isopropylidenedioxy)-2,5-bis(methoxymethoxy)cyclohexane (13) and (3*S*,4*R*,5*R*)-3,4-(isopropylidenedioxy)-5-(methoxymethoxy)-1-methylcyclohexene (14):** 7-Iodo-1,2,7-trideoxy-3,6-di-*O*-methoxymethyl-4,5-*O*-isopropylidene-d-*altro*-hept-1-ynitol **(9b**, 0.165 g, 0.397 mmol) was dissolved in dry benzene (10 ml) and the solution was degassed with argon and after 30 min placed under reflux. Tributyltin hydride (0.12 ml, 0.437 mmol) and AIBN (as catalyst) were added. After 30 min, tributyltin hydride (0.1 ml, 0.437 mmol) was dissolved in dry benzene (5 ml) and added very slowly to the reaction mixture over 20 min. After 2 h, the reaction mixture was cooled to room temperature, washed with light petroleum (40 ml) and extracted with acetonitrile (2 x 50 ml). The acetonitrile layers were combined and concentrated to be purified by flash chromatography on silica gel using light petroleum/diethyl ether (3:1) as eluent to afford the title compounds as 2 separable colourless oils **13** (0.044 g, 38%) and **14** (0.044 g, 49%).

**13**, *R_f_* = 0.55 in light petroleum:diethyl ether 1:2, [α]_D_ −83.4 (*c* 0.86 in CHCl_3_); ν_max_(film)/cm^-1^ 3077 (R_2_C=CH_2_), 2931, 2823, 1654 (C=C), 1446, 1380, 1261, 1214, 1149, 1106, 1037 and 917; δ_H_ (270 MHz) 1.37 (3 H, s, Me), 1.50 (3 H, s, Me), 2.46–2.70 (2 H, m, C*H_2_*), 3.38 (3 H, s, OMe), 3.40 (3 H, s, OMe), 4.02–4.07 (1 H, m, CH_2_C*H*OMOM), 4.13–4.19 (2 H, dd, *J* 5.28, 5.94 and 11.21, C*H*CH(OMOM)CH_2_ and C*H*CH(OMOM)C=), 4.47–4.50 (1 H, dd, *J* 2.64, 3.3 and 5.93, C*H*(OMOM)C=), 4.60–4.69 (2 H, 2d, *J* 6.59, OC*H_2_*O), 4.71–4.78 (2 H, m, OCH_2_O), 5.09 (2 H, d, *J* 11.21, C=CH_2_); δ_C_ (67.8 MHz) 25.25 (1 C, Me), 26.99 (1 C, Me), 31.61 (1 C, CH_2_), 55.45 (2 C, 2 x OMe), 72.20, 75.52, 76.72 and 78.67 (4 C, 4 x CHOR), 93.89 and 95.65 (2 C, 2 x O*C*H_2_O), 109.60 (1 C, *C*Me_2_), 114.29 and 139.67 (2 C, C=C); *m/z* (EI) 289 (MH^+^), 257, 246 and 188. [Found: M+H^+^ 289.1651. C_14_H_25_O_6_ requires 289.1651].

**14,** *R_f_* = 0.6 in light petroleum:diethyl ether 1:2, ν_max_(film)/cm^-1^ 2985, 2927, 1673 (C=C in a ring), 1442, 1376, 1222, 1149, 1106, 1072, 1037, 917, 875 and 755; δ_H_ (270 MHz) 1.39 (6 H, s, and 2 x Me), 1.73 (3 H, s, =C-C*H_3_*), 2.09–2.16 (1 H, dd, *J* 5.28 and 15.84, C*H*HC(Me)C=), 2.33–2.43 (1 H, bt, *J* 9.89 and 15.83, CH*H*C(Me)C=), 3.42 (3 H, s, OMe), 3.82–3.88 (1 H, ddd, *J* 2.64, 5.28 and 9.9, C*H*OMOM), 4.42 (1 H, d, *J* 3.3, CHOR), 4.60 (1 H, bs, CHOR), 4.74–4.80 (2 H, 2 x d, *J* 6.6 and 7.25, OC*H_2_*O), 5.33 (1 H, s, C*H*C=); δ_C_ (67.8 MHz) 23.34 (1 C, =C*Me*), 26.79 (1 C, Me), 27.66 (1 C, Me), 30.70 (1 C, *C*H_2_), 55.40 (1 C, OMe), 72.78, 74.28 and 74.51 (3 C, 3 x CHOR), 95.23 (1 C, O*C*H_2_O), 109.48 (1 C, *C*Me_2_), 120.62 and 134.65 (2 C, C=C); *m/z* (CI, NH_3_) 246 (M + NH^4+^), 229 and 188. [Found: M + NH_4_^+^ 246.1705. C_12_H_24_O_4_N requires 246.1705].

**2,5-Di-*O*-methoxymethyl-3,4-*O*-isopropylidene-5a-carba-α-l-gulopyranose (15):** (2*R*,3*S*,4*R*,5*R*)-1-methylidene-3,4-(isopropylidenedioxy)-2,5-bis(methoxymethoxy)cyclohexane **(13**, 0.041 g, 0.141mmol) was dissolved in dry THF (15 ml) and placed at 0 °C. Borane-methyl sulphide complex (2 M in THF, 0.1 ml, 0.2 mmol) was added and the reaction mixture was warmed up to room temperature. After 12 h, BH_3_-SMe_2_ (2 M, 0.1 ml, 0.2 mmol) was again added at 0 °C. After 2 h at the same temperature, water (1 ml) was added, followed by sodium hydroxide (2 N in water, 0.5 ml) and hydrogen peroxide (27%, 2.5 ml, 0.68 mmol). After 60 h at 0 °C, sodium hydroxide (2 N in water, 5 ml) was added, followed by water (40 ml). The crude product was extracted with ethyl acetate (3 x 40 ml). The organic layers were combined, dried (Na_2_SO_4_), concentrated and purified by flash chromatography on silica gel with diethyl ether to give the title compound **15** (0.041 g, 94%) as a colourless oil, ν_max_(film)/cm^-1^ 3455 (OH), 2927, 2854, 2360, 2337, 1731, 1646, 1461, 1380, 1265, 1214, 1149, 1106, 1037, 917 and 863; δ_H_ (270 MHz) (with the help of a ^1^H-^1^H NMR) 1.36 (3 H, s, Me), 1.51 (3 H, s, Me), 1.55–1.62 (1 H, dd, *J* 7.25 and 13.19, C*H*HCHOMOM), 1.66 (1 H, bs, OH), 1.82–1.87 (1 H, dd, *J* 3.3 and 9.9, CH*H*CHOMOM), 2.31 (1 H, m, C*H*CH_2_OH), 3.40 (3 H, s, OMe), 3.41 (3 H, s, OMe), 3.60 (2 H, d, *J* 6.59, C*H_2_*OH), 3.80–3.83 (1 H, t, *J* 3.3 and 2.64, C*H*(OMOM)CHCH_2_OH), 4.08–4.15 (1 H, qd, *J* 2.64, 7.25 and 9.89, CH_2_C*H*(OMOM)), 4.30–4.34 (1 H, dd, *J* 3.3, 2.63 and 7.26, C*H*CH(OMOM)CHCH_2_OH), 4.49–4.53 (1 H, dd, *J* 2.63 and 7.26, C*H*CH(OMOM)CH_2_), 4.63 (1 H, d, *J* 6.6, OC*H*HO), 4.71 (1 H, d, *J* 6.6, OCH*H*O), 4.75 (2 H, d, *J* 7.26, OC*H_2_*O); δ_C_ (67.8 MHz) 24.42 (1 C, Me), 26.62 (1 C, Me), 29.66 (1 C, *C*H_2_CHOMOM), 34.08 (1 C, *C*H_2_OH), 55.43–56.00 (2 C, 2 x OMe), 63.69 (1 C, *C*HCH_2_OH), 70.69, 75.03, 75.59 and 75.62 (4 C, 4 x CHOR), 95.54 and 96.73 (2 C, 2 x O*C*H_2_O), 109.49 (1 C, *C*Me_2_); *m/z* (EI) 307 (M^+^ + H^+^), 275, 243 and 144 [Found: MH^+^ 307.1757. C_14_H_27_O_7_ requires 307.1757].

**5a-Carba-α-l-gulopyranose (16):** 5,2-Di-*O*-methoxymethyl-4,3-*O*-isopropylidene-5a-carba-α-l-gulopyranose (**15**, 0.016 g, 0.052 mmol) was dissolved in methanol (2 ml) and HCl (6 N in water, 1 ml) was added at room temperature. After 12 h, the methanol was evaporated and the crude title compound **16** (0.010 g, 99%) was obtained and directly analysed, [α]_D_ 0 (*c* 1.56 in methanol), δ_H_ (270 MHz at 50 °C, in CD_3_OD) 1.70–1.74 (2 H, bt, *J* 3.63, CH(OH)C*H_2_*CH), 2.15–2.21 (1 H, bddd, *J* 2.72 and 6.84, C*H*CH_2_OH), 3.52–3.59 (1 H, dd, *J* 6.19 and 10.72, C*H*HOH), 3.64 (1 H, m, CH*H*OH), 3.66–3.73 (1 H, dd, *J* 6.76 and 10.64, C*H*OH), 3.83 (1 H, bs, C*H*OH), 4.01 (2 H, bt, *J* 2.72, 2 x C*H*OH).

**1,2,3,4,6-Penta*-O*-acetyl-5a-carba-α-l-gulopyranose (17):** 5a-Carba-α-l-gulopyranose (**16** ,0.010 g, 0.056 mmol) was dissolved in pyridine (1 ml). Acetic anhydride (1 ml) was added at room temperature. After 12 h, the solvents were evaporated (this operation was facilitated by the use of toluene) and the crude product was purified by a first flash chromatography on silica gel with diethyl ether and a second flash chromatography on silica gel with a solution of light petroleum/diethyl ether (1:4) as eluents to afford the title compound **17** (0.012 g, 57%) as a colourless oil, [α]_D_ −19.0 (*c* 0.42 in CHCl_3_); δ_H_ (270 MHz) 1.86–1.90 (2 H, td, *J* 3.96 and 8.9, C*H_2_*CHOAc), 2.05 (3 H, s, Me), 2.07 (3 H, s, Me), 2.08 (6 H, s, Me), 2.10 (3 H, s, Me), 2.62 (1 H, bm, C*H*CH_2_OAc), 3.97–4.04 (1 H, dd, *J* 6.6 and 11.21, C*H_2_*OAc) 4.06–4.12 (1 H, dd, *J* 6.6 and 11.21, C*H_2_*OAc), 5.23–5.32 (4 H, m, 4 x C*H*OAc); δ_C_ (67.8 MHz) 20.63, 20.66, 20.71, 20.79 and 20.88 (5 C, 5 x CO*Me*), 27.63 (1 C, CHOAc*C*H_2_CH), 29.70 (1 C, AcOCH_2_*C*H), 63.00 (1 C, *C*H_2_OAc), 67.11, 67.96, 68.55 and 69.19 (4 C, 4 x *C*HOAc), 169.33, 169.62, 169.86, 169.88 and 171.5 (5 C, 5 x C=O); *m/z* (CI, NH_3_) 364, 288 and 276. [Found: M + NH_4_^+^ 406.1713. C_17_H_28_O_10_N requires 406.1713].

**6,7-Dideoxy-3,4-*O*-isopropylidene-2,5-bis-*O*-(methoxymethyl)-7-phenyl-l-allohept-6-ynose (20):** The primary alcohol **6a** (1.58 g, 4.16 mmol) was dissolved in dichloromethane (30 ml). To this solution 4 Å molecular sieves (2.0 g) and pyridinium chlorochromate (2.69 g, 12.47 mmol) were added. The reaction mixture was stirred for 2 h and then poured onto a flash chromatography column packed with silica gel. Elution with diethyl ether and light petroleum (1:1) yielded the title compound **20** (1.34 g, 85%) as a colourless oil, which proved to be labile. [α]_D_ +58 (*c* 1.32, CHCl_3_); ν_max_(film)/cm^-1^ 2994, 2941, 2889, 2825, 2225 (C≡C), 1723 (C=O), 1605, 1493, 1446, 1378, 1259, 1213, 1160, 1098, 1028 and 918; δ_H_ (270 MHz) 1.37 (3 H, s, Me), 1.48 (3 H, s, Me), 3.43 (3 H, s, OMe), 3.48 (3 H, s, OMe), 4.51 (1 H, bd, *J* 4.31, C*H*CHO), 4.54–4.60 (2 H, m, *J* 2.1 and 6.5, C*H*CH(OMOM)CHO and C*H*CH(OMOM)C triple bond), 4.74 (1 H, d, *J* 6.3, OC*H*HO), 4.79 (1 H, d, *J* 6.3, OC*H*HO), 4.84 (1 H, d, *J* 6.3, OCH*H*O), 4.98 (1 H, d, *J* 6.6, C*H*(OMOM)C triple bond), 5.07 (1 H, d, *J* 6.3, OCH*H*O), 7.29–7.34 (3 H, m, Ph), 7.39–7.42 (2 H, m, Ph) and 9.78 (1 H, d, *J* 1.95, C*H*O); δ_C_ (67.8 MHz) 25.17 (1 C, Me), 26.78 (1 C, Me), 55.89 and 56.19 (2 C, 2 x OMe), 65.73 (1 C, *C*HCHO), 75.28, 78.49 and 79.89 (3 C, 3 x CHOR), 84.78 and 88.23 (2 C, C≡C), 94.30 and 97.38 (2 C, 2 x O*C*H_2_O), 109.68 (1 C, *C*Me_2_), 121.38 (1 C, Ph), 127.39 (2 C, Ph), 128.76 (1 C, Ph), 131.67 (2 C, Ph) and 200.18 (1 C, *C*HO).

**6,7-Dideoxy-3,4-*O*-isopropylidene-2,5-bis-*O*-(methoxymethyl)-7-phenyl-d-talohept-6-ynose (21):** The primary alcohol **6b** (1.24 g, 3.26 mmol) was dissolved in dichloromethane (30 ml); to this solution 4 Å molecular sieves (2.0 g) and pyridinium chlorochromate (2.11 g, 9.79 mmol) were added. After stirring for 2 h at room temperature, the reaction mixture was poured onto a flash chromatography column packed with silica gel and eluted with diethyl ether and light petroleum (1:1) to yield the title compound **21** (1.11 g, 90%) as a labile, colourless oil. [α]_D_ −43 (*c* 1.2, CHCl_3_); ν_max_(film)/cm^-1^ 3053, 2989, 2935, 2893, 2825, 2227 (C≡C), 1728 (C=O), 1601, 1494, 1445, 1380, 1258, 1211, 1158, 1105, 1029, and 917; δ_H_ (270 MHz) 1.37 (3 H, s, Me), 1.46 (3 H, s, Me), 3.36 (3 H, s, OMe), 3.46 (3 H, s, OMe), 4.48 (1 H, dd, *J* 2.0 and 4.9, C*H*CHO), 4.53–4.65 (2 H, m, C*H*CH(OMOM)CHO and C*H*CH(OMOM)C C≡C), 4.67–4.78 (3 H, m, OC*H*HO and OC*H_2_*O), 4.93 (1 H, d, *J* 6.59, C*H*(OMOM)C), 5.07 (1 H, d, *J* 6.0, OCH*H*O), 7.29–7.38 (3 H, m, Ph), 7.43–7.46 (2 H, m, Ph) and 9.73 (1 H, d, *J* 1.84, C*H*O); δ_C_ (67.8 MHz) 25.19 (1 C, Me), 26.85 (1 C, Me), 54.80 and 55.12 (2 C, 2 x OMe), 65.17 (1 C, *C*HCHO), 77.28, 78.29 and 80.29 (3 C, 3 x CHOR), 84.79 and 88.11 (2 C, Carbon triple bond), 94.19 and 96.38 (2 C, 2 x O*C*H_2_O), 109.58 (1 C, *C*Me_2_), 120.58 (1 C, Ph), 127.54 (2 C, Ph), 128.46 (1 C, Ph), 130.98 (2 C, Ph) and 197.88 (1 C, *C*HO).

**1,2-Dideoxy-1,7-diphenyl-3,6-di-*O*-methoxymethyl-4,5-*O*-isopropylidene-d-*allo*-hept-1-ynitol (22):** Phenylmagnesium chloride (1 M in THF, 39.3 ml, 39.3 mmol) was added to a solution of aldehyde **20** (1.47 g, 3.93 mmol) in dry THF (40 ml) at 0 °C. After 1 h, the solvent was removed under reduced pressure and the residue was dissolved in ethyl acetate (80 ml), washed with saturated aqueous NH_4_Cl (50 ml) and extracted with ethyl acetate (2 x 80 ml). The organic layers were combined, dried over Na_2_SO_4_, concentrated and purified by flash chromatography, using light petroleum/diethyl ether (1:1) as eluent to yield the title compound **22** (1.27 g, 72%) as colourless oil.

Diastereoisomer, *R_f_* = 0.36 in light petroleum/diethyl ether (2:1) **22**, [α]_D_ +9 (*c* 1.7, CHCl_3_); ν_max_(film)/cm^-1^ 3455 (OH), 3062, 3033, 2987, 2933, 2898, 2850, 2825, 2231 (CºC),1598, 1490, 1454, 1373, 1257, 1214, 1151, 1043, 970 and 919; δ_H_ (270 MHz) 1.37 (3 H, s, Me), 1.64 (3 H, s, Me), 3.40 (3 H, s, OMe), 3.43 (3 H, s, OMe), 4.05 (1 H, dd, *J* 2.0 and 7.3, C*H*(OH)Ph), 4.29 (1 H, d, *J* 7.3, O*H*), 4.38–4.41 (1 H, dd, *J* 3.3 and 4.6, C*H*CH(OH)Ph), 4.42 (1 H, dd, *J* 1.3 and 2.6, C*H*CHC), 4.62 (1 H, d, *J* 6.6, OC*H*HO), 4.64 (1 H, d, *J* 6.6, OC*H*HO), 4.70 (1 H, d, *J* 7.3, OCH*H*O), 4.87 (1 H, d, *J* 2.6, C*H*C), 5.01 (1 H, dd, *J* 4.0 and 5.9, C*H*CH CH(OH)Ph), 5.11 (1 H, d, *J* 6.6, OCH*H*O), 7.32–7.38 (6 H, m, Ph), and 7.42–7.49 (4 H, m, Ph); δ_C_ (67.8 MHz) 24.82 (1 C, Me), 26.78 (1 C, Me), 55.78 and 56.42 (2 C, 2 x OMe), 65.80 (1 C, *C*H(OH)Ph), 74.59, 76.53, 77.47 and 81.78 (4 C, 4 x CHOR), 85.08 and 87.83 (2 C, CºC), 93.99 and 98.50 (2 C, 2 x O*C*H_2_O), 109.32 (1 C, *C*Me_2_), 122.42 (1 C, Ph), 127.50 (2 C, Ph), 127.69 (1 C, Ph), 127.90 (2 C, Ph), 128.35 (2 C, Ph), 128.56 (1 C, Ph), 131.66 (2 C, Ph) and 139.83 (1 C, Ph); *m/z* (CI, NH_3_), 474 (M + NH_4_^+^), [Found: M + NH_4_^+^, 474.2484. C_26_H_36_O_7_N requires M + NH_4_^+^ 474.2492].

Diastereoisomer, *R_f_* = 0.32 in light petroleum/diethyl ether (2:1) **22**, [α]_D_ +11.5 (*c* 2.1, CHCl_3_); ν_max_(film)/cm^-1^ 3480 (OH), 3062, 3014, 2987, 2935, 2896, 2845, 2825, 2231 (CºC),1598, 1490, 1452, 1378, 1257, 1216, 1151, 1085, 1031 and 919; δ_H_ (270 MHz) 1.37 (3 H, s, Me), 1.66 (3 H, s, Me), 3.27 (3 H, s, OMe), 3.40 (3 H, s, OMe), 4.29–4.35 (2 H, dt, *J* 2.6 and 6.6, C*H*(OH)Ph and C*H*CH(OH)Ph), 4.43–4.49 (3 H, m, C*H*CH triple bond, C*H*CHCH(OH)Ph and OC*H*HO), 4.71 (1 H, d, *J* 5.9, OC*H*HO), 4.96 (1 H, d, *J* 3.3, C*H*C), 5.09 (2 H, d, *J* 6.6, OC*HH*O), 5.21 (1 H, s, OH), 7.21–7.38 (6 H, m, Ph), and 7.46–7.51 (4 H, m, Ph); δ_C_ (67.8 MHz) 24.85 (1 C, Me), 26.65 (1 C, Me), 55.74 and 56.09 (2 C, 2 x OMe), 66.09 (1 C, *C*H(OH)Ph), 73.00, 76.53, 78.89 and 79.37 (4 C, 4 x CHOR), 85.55 and 87.62 (2 C, CºC), 94.12 and 97.41 (2 C, 2 x O*C*H_2_O), 109.15 (1 C, *C*Me_2_), 122.48 (1 C, Ph), 126.01 (2 C, Ph), 127.32 (1 C, Ph), 128.11 (2 C, Ph), 128.38 (1 C, Ph), 128.58 (2 C, Ph), 128.66 (1 C, Ph), 131.63 (1 C, Ph) and 140.95 (1 C, Ph); *m/z* (CI, NH_3_), 474 (M + NH_4_^+^), [Found: M + NH_4_^+^, 474.2480. C_26_H_36_O_7_N requires M + NH_4_^+^ 474.2492].

**1,2-Dideoxy-1,7-diphenyl-3,6-di-*O*-methoxymethyl-4,5-*O*-isopropylidene-d-*altro*-hept-1-ynitols (23)** Phenylmagnesium chloride (1 M in THF, 24.76 ml, 24.76 mmol) was added to a solution of compound **21** (0.93 g, 2.48 mmol) in dry THF (20 ml) at 0 °C. After 1 h, the solvent was removed under reduced pressure and the residue was dissolved in ethyl acetate (50 ml), washed with saturated aqueous NH_4_Cl (30 ml) and the aqueous phase back-extracted with ethyl acetate (2 x 50 ml). The organic layers were combined, dried over Na_2_SO_4_, concentrated and purified by flash chromatography on silica gel with light petroleum/diethyl ether (3:1 to 1:1) as eluents to yield the title compounds **23** (0.8 g, 71%) as colourless oil.

Diastereoisomer, *R_f_* = 0.32 in light petroleum/diethyl ether (3:1) **23**, [α]_D_ +15 (*c* 1.2, CHCl_3_); ν_max_(film)/cm^-1^ 3483 (OH), 3089, 3049, 2989, 2946, 2887, 2823, 2223 (C≡C),1603, 1485, 1457, 1381, 1216, 1153, 1079, 1029 and 918; δ_H_ (270 MHz) 1.38 (3 H, s, Me), 1.61 (3 H, s, Me), 3.33 (3 H, s, OMe), 3.47 (3 H, s, OMe), 4.06 (1 H, dd, *J* 5.8 and 8.1, C*H*CHCH(OH)Ph), 4.39 (1 H, dd, *J* 4.7 and 5.0, C*H*CH(OH)Ph), 4.50 (1 H, dd, *J* 4.2 and 7.9, C*H*CHC C≡C), 4.57 (1 H, d, *J* 6.4, OC*H*HO), 4.68 (1 H, d, *J* 6.4, OC*H*HO), 4.73 (1 H, d, *J* 6.5, OC*H*HO), 4.87 (1 H, d, *J* 4.7, C*H*(OH)Ph), 4.98 (1 H, d, *J* 4.1, C*H*C triple bond), 5.1 (1 H, d, *J* 6.4, OC*H*HO), 5.14 (1 H, s, OH), 7.24–7.41 (6 H, m, Ph), and 7.45–7.49 (4 H, m, Ph); δ_C_ (67.8 MHz) 25.58 (1 C, Me), 26.98 (1 C, Me), 56.38 and 56.47 (2 C, 2 x OMe), 66.13 (1 C, *C*H(OH)Ph), 73.69, 77.21, 78.83 and 81.94 (4 C, 4 x CHOR), 85.68 and 87.73 (2 C, C≡C), 94.73 and 98.96 (2 C, 2 x O*C*H_2_O), 109.17 (1 C, *C*Me_2_), 122.38 (1 C, Ph), 127.49 (1 C, Ph), 127.65 (2 C, Ph), 127.78 (2 C, Ph), 128.29 (2 C, Ph), 128.63 (1 C, Ph), 131.83 (2 C, Ph) and 135.28 (1 C, Ph); *m/z* (CI, NH_3_), 474 (M + NH_4_^+^), [Found: M + NH_4_^+^, 474.2488. C_26_H_36_O_7_N requires M + NH_4_^+^ 474.2492].

Diastereoisomer, *R_f_* = 0.26 in light petroleum/diethyl ether (3:1) **23**, [α]_D_ −23 (*c* 1.8, CHCl_3_); ν_max_(film)/cm^-1^ 3521 (OH), 3057, 3029, 2986, 2939, 2886, 2829, 2223 (C≡C),1603, 1494, 1455, 1253, 1221, 1154, 1085, 1033 and 921; δ_H_ (270 MHz) 1.38 (3 H, s, Me), 1.61 (3 H, s, Me), 3.23 (3 H, s, OMe), 3.38 (3 H, s, OMe), 4.33 (1 H, d, *J* 6.4, C*H*CHCH(OH)Ph), 4.4 (1 H, d, *J* 6.5, OC*H*HO), 4.45 (1 H, dd, *J* 5.8 and 9.9, C*H*CH(OH)Ph), 4.52 (1 H, dd, *J* 2.1 and 6.0 , C*H*CHC C≡C), 4.58 (1 H, d, *J* 6.1, OC*H*HO), 4.74 (1 H, d, *J* 6.6, OC*H*HO), 4.93 (1 H, d, *J* 4.7, C*H*(OH)Ph), 5.09 (1 H, d, *J* 6.7, OC*H*HO), 5.18 (1 H, d, *J* 2.1, C*H*C triple bond), 5.24 (1 H, s, OH), 7.20–7.38 (6 H, m, Ph), and 7.43–7.47 (4 H, m, Ph); δ_C_ (67.8 MHz) 25.18 (1 C, Me), 26.33 (1 C, Me), 55.78 and 55.89 (2 C, 2 x OMe), 65.73 (1 C, *C*H(OH)Ph), 73.13, 77.25, 78.67 and 78.73 (4 C, 4 x CHOR), 85.38 and 87.59 (2 C, C≡C), 94.57 and 98.98 (2 C, 2 x O*C*H_2_O), 109.38 (1 C, *C*Me_2_), 122.38 (1 C, Ph), 126.12 (2 C, Ph), 127.09 (1 C, Ph), 127.83 (2 C, Ph), 128.13 (2 C, Ph), 128.37 (1 C, Ph), 131.53 (2 C, Ph) and 139.42 (1 C, Ph); *m/z* (CI, NH_3_), 474 (M + NH_4_^+^), [Found: M + NH_4_^+^, 474.2476. C_26_H_36_O_7_N requires M + NH_4_^+^ 474.2492].

**1,2-Dideoxy-7-methyl-3,6-di-*O*-methoxymethyl-4,5-*O*-isopropylidene-1-phenyl-d-*allo*-hept-1-ynitol (24):** Methylmagnesium bromide (1 M in THF, 44.5 ml, 44.5 mmol) was added to a solution of compound **20** (1.69 g, 4.46 mmol) in dry THF (40 ml) at 0 °C. After 1 h, the solvent was removed under reduced pressure and the residue was dissolved in ethyl acetate (80 ml), washed with saturated aqueous NH_4_Cl (50 ml) and extracted with ethyl acetate (2 x 80 ml). The organic layers were combined, dried over Na_2_SO_4_, concentrated and purified by flash chromatography, using light petroleum/diethyl ether (1:1) as eluent to give the title compound **24** (1.4 g 80%) as colourless oils.

Diastereoisomer, *R_f_* = 0.32 in light petroleum/diethyl ether (2:1) **24** [α]_D_ +68.18 (*c* 1.01 in CHCl_3_); ν_max_(film)/cm^-1^ 3444 (OH), 3079, 3014, 2985, 2937, 2894, 2827, 2284, 2231 (CºC), 1598, 1490, 1444, 1374, 1247, 1214, 1151, 1093, 1041, 973 and 916; δ_H_ (270 MHz) 1.26 (3 H, dd, *J* 7.26 and 13.86, CHCHC*H_3_*OH), 1.39 (3 H, s, Me), 1.57 (3 H, s, Me), 3.43 (3 H, s, OMe), 3.47 (3 H, s, OMe), 3.85 (1 H, d, *J* 9.9, CHO*H*), 4.01 (1 H, dq, J 2.0 and 6.6, CHC*H*CH_3_), 4.08–4.16 (2 H, m, C*H*CH(OH)CH_3_, and C*H*CHC), 4.47 (1 H, dd, *J* 2.6 and 6.6, C*H*CHCH(OH)CH_3_), 4.70 (1 H, d, *J* 7.3, OCH*H*O), 4.79 (3 H, m, OC*HH*O), 4.95 (1 H, d, *J* 4.0, C*H*C), 5.12 (1 H, d, *J* 6.6, OC*H*HO), 7.27–7.35 (3 H, m, Ph), and 7.41–7.46 (2 H, m, Ph); δ_C_ (67.8 MHz) 16.55 (1 C, MeOH), 24.96 (1 C, Me), 26.92 (1 C, Me), 55.85 and 56.10 (2 C, 2 x OMe), 65.35 (1 C, *C*H(OH)CH_3_), 67.42, 76.53, 77.47 and 83.49 (4 C, 4 x CHOR), 84.63 and 87.69 (2 C, CºC), 93.85 and 98.41 (2 C, 2 x O*C*H_2_O), 109.22 (1 C, *C*Me_2_), 122.25 (1 C, Ph), 128.31 (2 C, Ph), 128.57 (1 C, Ph) and 131.64 (2 C, Ph); *m/z* (CI, NH_3_), 412 (M + NH_4_^+^), [Found: M + NH_4_^+^, 412.2338. C_21_H_34_O_7_SiN requires M + NH_4_^+^ 412.2335].

Diastereoisomer, *R_f_* = 0.26 in light petroleum/diethyl ether (2:1) **24** [α]_D_ +51.9 (*c* 1.31 in CHCl_3_); ν_max_(film)/cm^-1^ 3469 (OH), 3054, 3040, 2983, 2935, 2896, 2825, 2782, 2231 (CºC), 1598, 1490, 1444, 1401, 1373, 1259, 1214, 1151, 1097, 1035 and 919; δ_H_ (270 MHz) 1.31 (3 H, dd, *J* 5.9 and 12.4, CHCHC*H_3_*OH), 1.41 (3 H, s, Me), 1.61 (3 H, s, Me), 2.90 (1 H, d, *J* 8.6, CHO*H*), 3.42 (3 H, s, OMe), 3.43 (3 H, s, OMe), 4.06–4.14 (2 H, m, CHC*H*(OH)CH_3_, and CHC*H*C triple bond) 4.41–4.46 (1 H, m, CHC*H*CH(OH)CH_3_), 4.52 (1 H, dd, *J* 2.6 and 7.3, C*H*CHC triple bond), 4.69–4.71 (1 H, d, *J* 7.3, OC*H*HO), 4.75–4.80 (2 H, m, OC*HH*O), 4.9 (1 H, d, *J* 2.6, CHC*H*CHC triple bond), 5.09 (1 H, d, *J* 7.3, OC*H*HO), 7.30–7.32 (3 H, m, Ph), and 7.42–7.45 (2 H, m, Ph); δ_C_ (67.8 MHz) 18.0 (1 C, MeOH), 24.84 (1 C, Me), 26.57 (1 C, Me), 55.77 and 56.09 (2 C, 2 x OMe), 65.85 (1 C, *C*H(OH)CH_3_), 68.35, 76.53, 78.46 and 79.08 (4 C, 4 x CHOR), 85.01 and 87.61 (2 C, carbon triple bond), 93.92 and 97.47 (2 C, 2 x O*C*H_2_O), 109.30 (1 C, *C*Me_2_), 122.34 (1 C, Ph), 128.28 (2 C, Ph), 128.47 (1 C, Ph) and 131.52 (2 C, Ph); *m/z* (CI, NH_3_) 412 (M + NH_4_^+^), [Found: M + NH_4_^+^, 412.2342. C_21_H_34_O_7_N requires M + NH_4_^+^ 412.2335].

**1,2-Dideoxy-7-methyl-3,6-di-*O*-methoxymethyl-4,5-*O*-isopropylidene-1-phenyl-d-*altro*-hept-1-ynitol (25):** Methylmagnesium bromide (1 M in THF, 22.38 ml, 22.38 mmol) was added to a solution of compound **21** (0.85 g, 2.24 mmol) in dry THF (20 ml) at 0 °C. After 1 h, the solvent was removed under reduced pressure and the residue was dissolved in ethyl acetate (50 ml), washed with saturated aqueous NH_4_Cl (30 ml) and extracted with ethyl acetate (2 x 50 ml). The organic layers were combined, dried over Na_2_SO_4_, concentrated and purified by flash chromatography, using light petroleum/diethyl ether (3:1 to 1:1) as eluent to yield the title compounds **25** (0.7 g, 79%) as colourless oil.

Diastereoisomer, *R_f_* = 0.34 in light petroleum/diethyl ether (3:1) **25**, [α]_D_ −14 (*c* 1.3, CHCl_3_); ν_max_(film)/cm^-1^ 3463 (OH), 3084, 3039, 2981, 2937, 2894, 2825, 2231 (C≡C), 1598, 1490, 1444, 1378, 1249, 1214, 1151, 1097, 1041 and 918; δ_H_ (270 MHz) 1.27 (3 H, dd, *J* 6.6 and 12.5, CHCHC*H_3_*OH), 1.37 (3 H, s, Me), 1.55 (3 H, s, Me), 2.89 (1 H, d, *J* 2.6, O*H*), 3.40 (3 H, s, OMe), 3.41 (3 H, s, OMe), 4.8 (1 H, dd, *J* 5.7 and 8.0, C*H*CHCH(OH)CH_3_), 4.42 (1 H, dd, *J* 6.6 and 7.9, C*H*CH(OH)CH_3_), 4.51 (1 H, dd, *J* 2.6 and 4.0, C*H*CHC triple bond), 4.67 (1 H, d, *J* 6.6, OC*H*HO), 4.76 (2 H, d, *J* 7.3, OC*HH*O), 4.88 (1 H, d, *J* 8.6, C*H*(OH)CH_3_), 4.95 (1 H, d, *J* 4.0, C*H*C), 5.07 (1 H, d, *J* 7.3, OC*H*HO), 7.26–7.30 (3 H, m, Ph), and 7.41–7.43 (2 H, m, Ph); δ_C_ (67.8 MHz) 17.55 (1 C, Me), 25.59 (1 C, Me), 27.02 (1 C, Me), 56.63 and 56.75 (2 C, 2 x OMe), 66.23 (1 C, *C*H(OH)CH_3_), 73.78, 77.25, 78.93 and 81.96 (4 C, 4 x CHOR), 85.69 and 87.59 (2 C), 94.78 and 98.93 (2 C, 2 x O*C*H_2_O), 109.13 (1 C, *C*Me_2_), 122.39 (1 C, Ph), 127.76 (1 C, Ph), 127.93 (2 C, Ph) and 128.28 (2 C, Ph); *m/z* (CI, NH_3_), 412 (M + NH_4_^+^), [Found: M + NH_4_^+^, 412.2336. C_21_H_34_O_7_N requires M + NH_4_^+^ 412.2335].

Diastereoisomer, *R_f_* = 0.26 in light petroleum/diethyl ether (3:1) **25** [α]_D_ −19 (*c* 1.1 in CHCl_3_); ν_max_(film)/cm^-1^ 3459 (OH), 3083, 3040, 2985, 2935, 2896, 2829, 2229 (C≡C), 1596, 1494, 1447, 1377, 1251, 1211, 1154, 1095, 1039 and 917; δ_H_ (270 MHz) 1.25 (3 H, dd, *J* 6.6 and 12.4, CHCHC*H_3_*OH), 1.35 (3 H, s, Me), 1.58 (3 H, s, Me), 2.90 (1 H, d, *J* 2.7, O*H*), 3.39 (3 H, s, OMe), 3.40 (3 H, s, OMe), 4.06 (1 H, dd, *J* 5.8 and 5.4, C*H*CHCH(OH)CH_3_), 4.42 (1 H, dd, *J* 6.6 and 4.5, C*H*CH(OH)CH_3_), 4.49 (1 H, dd, *J* 2.6 and 3.9, C*H*CHC triple bond), 4.65 (1 H, d, *J* 6.6, OC*H*HO), 4.73 (2 H, d, *J* 7.2, OC*HH*O), 4.85 (1 H, d, *J* 8.0, C*H*(OH)CH_3_), 4.94 (1 H, d, *J* 5.0, C*H*C), 5.06 (1 H, d, *J* 7.2, OC*H*HO), 7.25–7.29 (3 H, m, Ph), and 7.40–7.42 (2 H, m, Ph); δ_C_ (67.8 MHz) 17.52 (1 C, Me), 25.63 (1 C, Me), 27.13 (1 C, Me), 56.61 and 56.72 (2 C, 2 x OMe), 66.24 (1 C, *C*H(OH)CH_3_), 73.75, 77.26, 78.88 and 81.94 (4 C, 4 x CHOR), 85.73 and 87.62 (2 C, C≡C), 94.82 and 98.89 (2 C, 2 x O*C*H_2_O), 109.11 (1 C, *C*Me_2_), 122.42 (1 C, Ph), 127.79 (1 C, Ph), 127.96 (2 C, Ph) and 128.31 (2 C, Ph); *m/z* (CI, NH_3_), 412 (M + NH_4_^+^), [Found: M + NH_4_^+^, 412.2344. C_21_H_34_O_7_SiN requires M + NH_4_^+^ 412.2335].

**1,2-Dideoxy-7-iodo-1,7-diphenyl-3,6-di-*O*-methoxymethyl-4,5-*O*-isopropylidene-d-*allo*-hept-1-ynitol (26):** The secondary alcohols **22** (1.40 g, 3.08 mmol) were converted to the iodides **26** using the same method as for compound **27**, which afforded the title compound **26** as an inseparable mixture of isomers (1.36 g, 78%) and as a colourless oil. ν_max_(film)/cm^-1^ 3083, 3059, 3029, 2987, 2931, 2896, 2831, 2253 (CºC),1604, 1495, 1453, 1385, 1250, 1210, 1149, 1106, 1043 and 983; δ_H_ (270 MHz) 1.18 (3 H, s, Me), 1.59 (3 H, s, Me), 3.40 (3 H, s, OMe), 3.43 (3 H, s, OMe), 3.92 (1 H, dd, *J* 3.9 and 9.2, C*H*CHC), 4.13 (1 H, d, *J* 7.3, OC*H*HO), 4.39 (1 H, dd, *J* 2.6 and 9.2, C*H*CHCHIPh), 4.61 (1 H, dd, *J* 2.6 and 8.3, C*H*CHI), 4.69 (1 H, d, *J* 6.6, OC*H*HO), 5.05–5.08 (2 H, m, C*H*I and OC*H*HO), 5.21 (1 H, d, *J* 6.6, OC*H*HO), 5.63 (1 H, d, *J* 2.6, C*H*C triple bond), 7.23–7.26 (3 H, m, Ph), 7.33–7.35 (3 H, m, Ph), 7.45–7.48 (2 H, m, *ortho*-Ph) and 7.53–7.56 (2 H, m, *ortho*-Ph); δ_C_ (67.8 MHz) 24.66 (1 C, Me), 26.62 (1 C, Me), 32.09 (1 C, *C*HI), 55.87 and 56.26 (2 C, 2 x OMe), 65.85, 76.53, 76.68, and 77.73 (4 C, 4 x CHOR), 85.48 and 87.67 (2 C, CºC), 95.27 and 98.78 (2 C, 2 x O*C*H_2_O), 108.77 (1 C, *C*Me_2_), 122.54 (1 C, Ph), 127.72 (2 C, Ph), 127.94 (1 C, Ph), 128.32 (2 C, Ph), 128.45 (1 C, Ph), 130.13 (2 C, Ph), 131.62 (2 C, Ph) and 138.98 (1 C, Ph).

*NOTE: A molecular ion was not observed in the MS*.

**1,2-Dideoxy-7-iodo-1,7-diphenyl-3,6-di-*O*-methoxymethyl-4,5-*O*-isopropylidene-d-*altro*-hept-1-ynitol (27):** The secondary alcohols **23** (0.70 g, 1.54 mmol) were dissolved in dry toluene (20 ml); to this solution triphenylphosphine (1.21 g, 4.17 mmol) and imidazole (0.31 g, 4.17 mmol) were added. The reaction mixture was warmed to 60 °C. To the resultant mixture, iodine (0.39 g, 3.08 mmol) was added. After 2 h, the reaction was poured directly onto saturated aqueous sodium thiosulphate (50 ml) and extracted with ethyl acetate (3 x 70 ml). The organic layers were combined, dried over Na_2_SO_4_ and evaporated under reduced pressure. It was observed that the product decomposed during chromatography as evidenced by colouration of the silica gel. Flash chromatography was thus conducted as rapidly as possible, on silica gel with light petroleum/diethyl ether (8:1) as eluent to yield the title compound **27** as an inseparable mixture of isomers (0.64 g, 73%) as a colourless oil. ν_max_(film)/cm^-1^ 3074, 3058, 3022, 2986, 2948, 2898, 2858, 2838, 2236 (CºC),1583, 1494, 1455, 1391, 1253, 1221, 1148, 1085, 1033 and 921; δ_H_ (270 MHz) 1.19 (3 H, s, Me), 1.57 (3 H, s, Me), 3.40 (3 H, s, OMe), 3.48 (3 H, s, OMe), 3.89 (1 H, dd, *J* 5.7 and 8.3, C*H*CHIPh), 4.32 (1 H, dd, *J* 3.1 and 5.7, C*H*CHI), 4.63 (1 H, dd, *J* 2.9 and 8.3, C*H*CHC), 4.67 (1 H, d, *J* 6.7, OC*H*HO), 5.03 (1 H, d, *J* 2.5, C*H*I), 5.06 (1 H, d, *J* 6.5, OC*H*HO), 5.09 (1 H, d, *J* 6.7, OC*H*HO), 5.14 (1 H, d, *J* 6.6, OC*H*HO), 5.70 (1 H, d, *J* 2.9, C*H*C), 7.16–7.23 (3 H, m, Ph), 7.23–7.32 (3 H, m, Ph), 7.42–7.48 (2 H, m, Ph) and 7.53–7.56 (2 H, m, Ph); δ_C_ (67.8 MHz) 23.81 (1 C, Me), 25.72 (1 C, Me), 32.54 (1 C, *C*HI), 55.28 and 54.83 (2 C, 2 x OMe), 65.13, 73.13, 77.25, and 78.73 (4 C, 4 x CHOR), 84.37 and 85.93 (2 C, CºC), 94.27 and 98.45 (2 C, 2 x O*C*H_2_O), 109.09 (1 C, *C*Me_2_), 121.64 (1 C, Ph), 126.97 (2 C, Ph), 127.28 (1 C, Ph), 127.84 (2 C, Ph), 128.13 (1 C, Ph), 129.81 (2 C, Ph), 131.73 (2 C, Ph) and 138.76 (1 C, Ph).

*NOTE: All attempts to obtain mass spectrometry data proved fruitless employing the different ionisation techniques available.*

**1,2-Dideoxy-7-iodo-7-methyl-3,6-di-*O*-methoxymethyl-4,5-*O*-isopropylidene-1-phenyl-d-*allo*-hept-1-ynitol (28):** The secondary alcohols **24** (1.35 g, 3.42 mmol) were converted to the iodides **28** using the same method as for compound **27**, which afforded title compound **28** as an inseparable mixture of isomers (1.41 g, 85%) as a colourless oil. [α]_D_ +16.5 (*c* 0.49 in CHCl_3_); ν_max_(film)/cm^-1^ 3079, 3049, 3018, 2993, 2935, 2894, 2843, 2825, 2782, 2231 (CºC), 1598, 1490, 1392, 1380, 1249, 1214, 1149, 1108, 1039 and 968; δ_H_ (270 MHz) 1.40 (3 H, s, Me), 1.60 (3 H, s, Me), 1.93 (3 H, d, *J* 7.3, CHIC*H*_3_), 3.37 (3 H, s, OMe), 3.41 (3 H, s, OMe), 3.90 (1 H, dd, *J* 2.0 and 3.2, C*H*CHC triple bond), 4.43 (1 H, dd, *J* 6.6 and 7.9, OC*H*HO), 4.49 (1 H, dd, *J* 2.6 and 6.6, C*H*CHCHCH_3_I), 4.68–4.75 (2 H, m, CHC*H*CHCH_3_I and CHC*H*CH_3_I), 4.84 (1 H, d, *J* 7.3, OC*H*HO), 4.90–4.94 (2 H, m, OC*HH*O), 5.09 (1 H, d, *J* 3.3, C*H*C), 7.29–7.33 (3 H, m, Ph) and 7.43–7.47 (2 H, m, Ph); δ_C_ (67.8 MHz) 22.72 (1 C, Me), 24.79 (1 C, Me), 26.62 (1 C, MeI), 31.20 (1 C, *C*HI), 55.57 and 56.73 (2 C, 2 x OMe), 65.96, 76.53, 77.47, and 78.20 (4 C, 4 x CHOR), 85.18 and 87.66 (2 C, CºC), 94.28 and 98.97 (2 C, 2 x O*C*H_2_O), 109.25 (1 C, *C*Me_2_), 122.44 (1 C, Ph), 128.36 (2 C, Ph), 128.55 (1 C, Ph) and 131.60 (2 C, Ph).

**1,2-Dideoxy-7-iodo-7-methyl-3,6-di-*O*-methoxymethyl-4,5-*O*-isopropylidene-1-phenyl-d-*altro*-hept-1-ynitol (29):** The secondary alcohols **25** (0.68 g, 1.71 mmol), were dissolved in dry toluene (20 ml); to this solution triphenylphosphine (1.21 g, 4.17 mmol) and imidazole (0.31 g, 4.17 mmol) were added. The reaction mixture was warmed to 60 °C. To the resultant mixture, iodine (0.39 g, 3.08 mmol) was added. After 2 h, the reaction was poured directly onto saturated aqueous sodium thiosulphate (50 ml) and extracted with ethyl acetate (3 x 70 ml). The organic layers were combined, dried over Na_2_SO_4_ and evaporated under reduced pressure. It was observed that the product decomposed during chromatography as evidenced by colouration of the silica gel. Flash chromatography was thus conducted as rapidly as possible, on silica gel with light petroleum/diethyl ether (8:1) as eluent to yield the title compound as an inseparable mixture of isomers **29** (0.73 g, 85%) as a colourless oil. [α]_D_ −18.48 (*c* 0.55 in CHCl_3_); ν_max_(film)/cm^-1^ 3080, 3055, 3021, 2985, 2943, 2898, 2855, 2832, 2773, 2232 (CºC), 1587, 1483, 1391, 1384, 1252, 1216, 1148, 1108, 1034 and 974; δ_H_ (270 MHz) 1.38 (3 H, s, Me), 1.58 (3 H, s, Me), 1.91 (3 H, d, *J* 7.21, CHICH_3_), 3.38 (3 H, s, OMe), 3.42 (3 H, s, OMe), 3.89 (1 H, dd, *J* 2.0 and 8.4, C*H*CHC triple bond), 4.42 (1 H, dd, *J* 5.9 and 7.9, OC*H*HO), 4.49 (1 H, dd, *J* 2.6 and 6.6, C*H*CHCHCH_3_I), 4.65–4.73 (2 H, m, CHC*H*CHCH_3_I and CHC*H*CH_3_I), 4.84 (1 H, d, *J* 7.3, OC*H*HO), 4.89–4.93 (2 H, m, OC*HH*O), 5.03–5.06 (1 H, d, *J* 8.6, C*H*C), 7.28–7.34 (3 H, m, Ph) and 7.37–7.39 (2 H, m, Ph); δ_C_ (67.8 MHz) 22.82 (1 C, Me), 24.93 (1 C, Me), 26.82 (1 C, MeI), 31.73 (1 C, *C*HI), 55.63 and 56.13 (2 C, 2 x OMe), 66.12, 76.73, 77.38, and 78.19 (4 C, 4 x CHOR), 85.83 and 87.58 (2 C, CºC), 96.28 and 97.18 (2 C, 2 x O*C*H_2_O), 110.28 (1 C, *C*Me_2_), 124.82 (1 C, Ph), 128.27 (2 C, Ph), 128.78 (1 C, Ph) and 133.62 (2 C, Ph).

**(1*Z*,2*R*,3*S*,4*R*,5*R*,6*S*)-1-Benzylidene-3,4-(isopropylidenedioxy)-2,5-bis(methoxymethoxy)-6-phenylcyclohexane (30), (1*E*,2*R*,3*S*,4*R*,5*R*,6*S*)-1-benzylidene-3,4-(isopropylidenedioxy)-2,5-bis(methoxymethoxy)-6-phenylcyclohexane (31), (1*Z*,2*R*,3*S*,4*R*,5*R*,6*R*)-1-benzylidene-3,4-(isopropylidenedioxy)-2,5-bis(methoxymethoxy)-6-phenylcyclohexane (32), (1*E*,2*R*,3*S*,4*R*,5*R*,6*R*)-1-benzylidene-3,4-(isopropylidenedioxy)-2,5-bis(methoxymethoxy)-6-phenylcyclohexane (33):** The iodides **27** (0.37 g, 0.65 mmol) were dissolved in dry benzene (50 ml) and the solution was degassed by bubbling argon gas through the solution for 40 min. The solution was placed under reflux at 80 °C, a catalytic amount of AIBN was added followed by the addition of tributyltin hydride (0.21 g, 0.19 ml, 0.72 mmol) over 20 min. After 3 h at reflux the reaction mixture was cooled to RT and concentrated. The residue was dissolved in acetonitrile (100 ml), washed with light petroleum (40 ml). The acetonitrile phase was evaporated under reduced pressure. The resultant product was purified by flash chromatography using light petroleum/diethyl ether (3:1 to 1:1) as eluent to give the title compounds as colourless oils **30** (0.09 g, 32%), **31** (0.07 g, 24%), **32** (0.05 g, 16%) and **33** (0.06 g, 19%). Data detailed below:

**(1*Z*,2*R*,3*S*,4*R*,5*R*,6*S*)-1-Benzylidene-3,4-(isopropylidenedioxy)-2,5-bis(methoxymethoxy)-6-phenylcyclohexane (30):** [α]_D_ +56.4 (*c* 1.3 in CHCl_3_); ν_max_(film)/cm^-1^ 3074, 3058, 3023, 2987, 2950, 2923, 2895, 2854, 2838, 1734, 1598, 1494, 1455, 1391, 1378, 1253, 1216, 1148, 1107, 1075, 1030 and 918; δ_H_ (270 MHz) (with the aid of 2D ^1^H NMR) 1.47 (3 H, s, Me), 1.68 (3 H, s, Me), 3.09 (3 H, s, OMe), 3.24 (3 H, s, OMe), 4.06 (1 H, d, *J* 6.7, OC*H*HO), 4.12 (1 H, d, *J* 9.6, CHC*H*(Ph)C=), 4.14 (1 H, d, *J* 9.6, CHC*H*(OMOM)C=), 4.31 (2 x 1H, d, *J* 6.5,OC*H*HO and OCH*H*O), 4.45 (1 H, dd, *J* 4.2 and 9.6, CHC*H*(OMOM)CH(Ph)C=), 4.58 (1 H, d, *J* 6.8, OCH*H*O), 4.69 (1 H, dd, *J* 4.7 and 9.7 CHC*H*CH(OMOM)C=), 4.70 (1 H, dd, *J* 4.5 and 6.6, C*H*OR), 6.43 (1 H, s, C=C*H*Ph), 7.16–7.41 (10 H, m, Ph); δ_C_ (67.8 MHz) 25.08 (1 C, Me), 25.72 (1 C, Me), 48.27 (1 C, C*H*Ph), 55.46 and 55.67 (2 C, 2 x OMe), 72.58, 75.02, 76.25, and 76.45 (4 C, 4 x CHOR), 94.27 and 96.45 (2 C, 2 x O*C*H_2_O), 110.39 (1 C, *C*Me_2_), 126.67 (1 C, Ph), 126.77 (1 C, Ph), 127.28 (2 C, Ph), 128.51 (2 C, Ph), 128.89 (2 C, Ph), 129.18 (1 C, Ph), 131.73 (2 C, Ph) and 136.66 (1 C, Ph), 137.30 and 141.75 (2 C, *C*=*C*HPh); *m/z* (CI, NH_3_), 458 (M + NH_4_^+^), [Found: M + NH_4_^+^, 458.2543. C_26_H_36_O_6_N requires M + NH_4_^+^ 458.2543].

**(1*E*,2*R*,3*S*,4*R*,5*R*,6*S*)-1-Benzylidene-3,4-(isopropylidenedioxy)-2,5-bis(methoxymethoxy)-6-phenylcyclohexane (31):** [α]_D_ −19.4 (*c* .8 in CHCl_3_); ν_max_(film)/cm^-1^ 3074, 3058, 3023, 2987, 2950, 2923, 2895, 2854, 2838, 1734, 1598, 1494, 1455, 1391, 1378, 1253, 1216, 1148, 1107, 1075, 1030 and 918; δ_H_ (270 MHz) (with the help of 2D ^1^H NMR) 1.47 (3 H, s, Me), 1.68 (3 H, s, Me), 3.09 (3 H, s, OMe), 3.24 (3 H, s, OMe), 4.06 (1 H, d, *J* 6.7, OC*H*HO), 4.11 (1 H, d, *J* 9.8, CHC*H*(Ph)C=), 4.11 (1 H, d, *J* 9.8, CHC*H*(OMOM)C=), 4.32 (2 x 1H, d, *J* 6.5, OC*H*HO and OCH*H*O), 4.44 (1 H, dd, *J* 4.2 and 9.8, CHC*H*(OMOM)CH(Ph)C=), 4.58 (1 H, d, *J* 6.8, OCH*H*O), 4.69 (1 H, dd, *J* 4.7 and 9.8 CHC*H*CH(OMOM)C=), 4.72 (1 H, dd, *J* 4.5 and 6.6, C*H*OR), 6.42 (1 H, s, C=C*H*Ph), 7.15–7.41 (10 H, m, Ph); δ_C_ (67.8 MHz) 25.08 (1 C, Me), 25.72 (1 C, Me), 48.27 (1 C, C*H*Ph), 55.46 and 55.67 (2 C, 2 x OMe), 72.58, 75.02, 76.25, and 76.45 (4 C, 4 x CHOR), 94.27 and 96.45 (2 C, 2 x O*C*H_2_O), 110.39 (1 C, *C*Me_2_), 126.67 (1 C, Ph), 126.77 (1 C, Ph), 127.28 (2 C, Ph), 128.51 (2 C, Ph), 128.89 (2 C, Ph), 129.18 (1 C, Ph), 131.74 (2 C, Ph) and 136.68 (1 C, Ph), 137.29 and 141.39 (2 C, *C*=*C*HPh); *m/z* (CI, NH_3_), 458 (M + NH_4_^+^), [Found: M + NH_4_^+^, 458.2546. C_26_H_36_O_6_N requires M + NH_4_^+^ 458.2548].

**(1*Z*,2*R*,3*S*,4*R*,5*R*,6*R*)-1-Benzylidene-3,4-(isopropylidenedioxy)-2,5-bis(methoxymethoxy)-6-phenylcyclohexane (32):** [α]_D_ +47.2 (*c* 1.1 in CHCl_3_); ν_max_(film)/cm^-1^ 3071, 3058, 3021, 2986, 2953, 2925, 2895, 2851, 2838, 1737, 1598, 1492, 1457, 1391, 1380, 1255, 1218, 1148, 1106, 1075, 1032 and 919; δ_H_ (270 MHz) (with the help of 2D ^1^H NMR) 1.46 (3 H, s, Me), 1.67 (3 H, s, Me), 3.08 (3 H, s, OMe), 3.23 (3 H, s, OMe), 4.06 (1 H, d, *J* 6.7, OC*H*HO), 4.12 (1 H, d, *J* 5.6, CHC*H*(Ph)C=), 4.16 (1 H, d, *J* 9.6, CHC*H*(OMOM)C=), 4.33 (2 x 1H, d, *J* 6.5, OC*H*HO and OCH*H*O), 4.46 (1 H, dd, *J*4.2 and 5.6, CHC*H*(OMOM)CH(Ph)C=), 4.58 (1 H, d, *J* 6.8, OCH*H*O), 4.69 (1 H, dd, *J* 4.7 and 9.7 CHC*H*CH(OMOM)C=), 4.71 (1 H, dd, *J* 4.5 and 6.6, C*H*OR), 6.43 (1 H, s, C=C*H*Ph), 7.16–7.41 (10 H, m, Ph); δ_C_ (67.8 MHz) 25.07 (1 C, Me), 25.77 (1 C, Me), 48.29 (1 C, C*H*Ph), 55.47 and 55.69 (2 C, 2 x OMe), 72.82, 75.14, 76.29, and 76.45 (4 C, 4 x CHOR), 94.46 and 96.69 (2 C, 2 x O*C*H_2_O), 111.09 (1 C, *C*Me_2_), 126.67 (1 C, Ph), 126.77 (1 C, Ph), 127.28 (2 C, Ph), 128.51 (2 C, Ph), 128.89 (2 C, Ph), 129.18 (1 C, Ph), 131.77 (2 C, Ph) and 136.68 (1 C, Ph), 137.33 and 141.78 (2 C, *C*=*C*HPh); *m/z* (CI, NH_3_), 458 (M + NH_4_^+^), [Found: M + NH_4_^+^, 458.2545. C_26_H_36_O_6_N requires M + NH_4_^+^ 458.2544].

**(1*E*,2*R*,3*S*,4*R*,5*R*,6*R*)-1-Benzylidene-3,4-(isopropylidenedioxy)-2,5-bis(methoxymethoxy)-6-phenylcyclohexane (33):** [α]_D_ −26.4 (*c* 0.8 in CHCl_3_); ν_max_(film)/cm^-1^ 3069, 3059, 3021, 2986, 2953, 2925, 2895, 2851, 2838, 1737, 1598, 1492, 1457, 1391, 1380, 1255, 1218, 1148, 1106, 1075, 1032 and 918; δ_H_ (270 MHz) (with the help of 2D ^1^H NMR) 1.45 (3 H, s, Me), 1.67 (3 H, s, Me), 3.08 (3 H, s, OMe), 3.22 (3 H, s, OMe), 4.06 (1 H, d, *J* 6.7, OC*H*HO), 4.14 (1 H, d, *J* 5.6, CHC*H*(Ph)C=), 4.17 (1 H, d, *J* 9.8, CHC*H*(OMOM)C=), 4.31 (2 x 1H, d, *J* 6.5, OC*H*HO and OCH*H*O), 4.46 (1 H, dd, *J*4.2 and 5.6, CHC*H*(OMOM)CH(Ph)C=), 4.58 (1 H, d, *J* 6.8, OCH*H*O), 4.70 (1 H, dd, *J* 4.7 and 9.8 CHC*H*CH(OMOM)C=), 4.71 (1 H, dd, *J* 4.5 and 6.6, C*H*OR), 6.41 (1 H, s, C=C*H*Ph), 7.16–7.41 (10 H, m, Ph); δ_C_ (67.8 MHz) 25.07 (1 C, Me), 25.77 (1 C, Me), 48.29 (1 C, C*H*Ph), 55.49 and 55.70 (2 C, 2 x OMe), 72.81, 75.19, 76.29, and 76.48 (4 C, 4 x CHOR), 94.48 and 96.73 (2 C, 2 x O*C*H_2_O), 111.09 (1 C, *C*Me_2_), 126.67 (1 C, Ph), 126.77 (1 C, Ph), 127.28 (2 C, Ph), 128.51 (2 C, Ph), 128.89 (2 C, Ph), 129.18 (1 C, Ph), 131.77 (2 C, Ph) and 137.18 (1 C, Ph), 137.38 and 141.48 (2 C, *C*=*C*HPh); *m/z* (CI, NH_3_), 458 (M + NH_4_^+^), [Found: M + NH_4_^+^, 458.2547. C_26_H_36_O_6_N requires M + NH_4_^+^ 458.2546].

**(1*Z*,2*R*,3*S*,4*R*,5*R*,6*S*)-1-Benzylidene-3,4-(isopropylidenedioxy)-2,5-bis(methoxymethoxy)-6-methylcyclohexane (34), (1*E*,2*R*,3*S*,4*R*,5*R*,6*S*)-1-benzylidene-3,4-(isopropylidenedioxy)-2,5-bis(methoxymethoxy)-6-methylcyclohexane (35), (1*Z*,2*R*,3*S*,4*R*,5*R*,6*R*)-1-benzylidene-3,4-(isopropylidenedioxy)-2,5-bis(methoxymethoxy)-6-methylcyclohexane (36), (1*E*,2*R*,3*S*,4*R*,5*R*,6*R*)-1-benzylidene-3,4-(isopropylidenedioxy)-2,5-bis(methoxymethoxy)-6-methylcyclohexane (37):** The iodides **29** (0.39 g, 0.77 mmol) were dissolved in dry benzene (50 ml) and the solution was degassed by bubbling argon through the solution for 40 min. The solution was refluxed and a catalytic amount of AIBN was added. Tributyltin hydride (0.25 g, 0.23 ml, 0.84 mmol) was added dropwise to the boiling mixture over 30 min. After a further 3 h under reflux the reaction mixture was concentrated to give a yellow oil. The residue was dissolved in acetonitrile (100 ml), washed with light petroleum (40 ml). The petroleum layer extracted with acetonitrile (2 x 100 ml). The layers of acetonitrile were combined and evaporated under reduced pressure. The residue was purified by flash chromatography with light petroleum/diethyl ether (3:1 to 1:1) as eluent to obtain the title compounds as colourless oils **34** (0.09 g, 30%), **35** (0.07 g, 23%), **36** (0.05 g, 17%) and **37** (0.04 g, 14%).

**(1*Z*,2*R*,3*S*,4*R*,5*R*,6*S*)-1-Benzylidene-3,4-(isopropylidenedioxy)-2,5-bis(methoxymethoxy)-6-methylcyclohexane (34):** *R_f_* = 0.72 in light petroleum/diethyl ether (4:6); [α]_D_ +38.2 (*c* 1.65 in CHCl_3_); ν_max_ (film)/cm^-1^ 3057, 3021, 2985, 2934, 2857, 2828, 2782, 2253, 1726, 1606, 1495, 1464, 1380, 1246, 1217, 1153, 1106, 1074, 1035 and 938; δ_H_ (270 MHz) (with the help of 2D ^1^H NMR) 1.27 (3 H, d, *J* 6.6, CHMe), 1.43 (3 H, s, Me), 1.65 (3 H, s, Me), 3.06 (1 H, dq, *J* 10.6 and 6.6, CHC*H*Me), 3.19 (3 H, s, OMe), 3.49 (3 H, s, OMe), 4.20–4.25 (2 H, m, CHC*H*CH(OMOM)C= and CHC*H*CH(OMOM)CH), 4.26 (1 H, d, *J* 6.6, OC*H*HO), 4.39 (1 H, d, *J* 6.6, OC*H*HO), 4.54 (1 H, d, *J* 9.6, CHC*H*(OMOM)C=), 4.61 (1 H, dd, *J* 4.0 and 9.5 CHC*H*(OMOM)CH), 4.73 (1 H, d, *J* 6.3, OCH*H*O), 4.86 (1 H, d, *J* 6.6, OCH*H*O), 6.63 (1 H, s, C=C*H*Ph), 7.16–7.32 (5 H, m, Ph); δ_C_ (67.8 MHz) 15.45 (1 C, CH*Me*), 25.41 (1 C, Me), 25.69 (1 C, Me), 41.37 (1 C, *C*HMe), 55.32 and 55.74 (2 C, 2 x OMe), 71.35, 74.37, 76.35, and 79.74 (4 C, 4 x CHOR), 94.35 and 96.15 (2 C, 2 x O*C*H_2_O), 111.14 (1 C, *C*Me_2_), 126.74 (1 C, Ph), 127.88 (2 C, Ph), 128.08 (1 C, Ph), 128.56 (2 C, Ph), 136.64 and 139.11 (2 C, *C*=*C*HPh); *m/z* (CI, NH_3_), 396 (M + NH_4_^+^), [Found: M + NH_4_^+^, 396.2383. C_21_H_34_O_6_N requires M + NH_4_^+^ 396.2386].

**(1*E*,2*R*,3*S*,4*R*,5*R*,6*S*)-1-Benzylidene-3,4-(isopropylidenedioxy)-2,5-bis(methoxymethoxy)-6-methylcyclohexane (35):** *R_f_* = 0.43 in light petroleum/diethyl ether (4:6); [α]_D_-39.0 (*c* 1.68 in CHCl_3_); ν_max_ (film)/cm^-1^ 3049, 3012, 2954, 2986, 2879, 2858, 2778, 2358, 2341, 1729, 1669, 1598, 1494, 1461, 1387, 1247, 1214, 1157, 1109, 1039, 945 and 917; δ_H_ (270 MHz) (with the help of 2D ^1^H NMR) 1.14 (3 H, d, *J* 5.3, CHMe), 1.34 (3 H, s, Me), 1.44 (3 H, s, Me), 3.16 (1 H, dq, *J* 10.2 and 5.3, CHC*H*Me), 3.44 (3 H, s, OMe), 3.47 (3 H, s, OMe), 3.60 (1 H, dd, *J* 2.6 and 10.2, CHC*H*(OMOM)CHMe), 4.24 (1 H, m, CHC*H*CH(OMOM)CH), 4.32 (1 H, d, *J* 6.6, OC*H*HO), 4.56 (1 H, dd, *J* 9.9 and 7.3, CHC*H*CH(OMOM)C=), 4.71 (1 H, d, *J* 7.2, OC*H*HO), 4.79 (1 H, d, *J* 9.9, CHC*H*(OMOM)C=), 4.79 (1 H, d, *J* 7.3, OCH*H*O), 4.83 (1 H, d, *J* 7.2, OCH*H*O), 6.56 (1 H, s, C=C*H*Ph), 7.18–7.39 (5 H, m, Ph); δ_C_ (67.8 MHz) 17.87 (1 C, CH*Me*), 24.47 (1 C, Me), 25.79 (1 C, Me), 34.54 (1 C, *C*HMe), 55.64 and 55.78 (2 C, 2 x OMe), 72.59, 75.45, 77.47, and 77.89 (4 C, 4 x CHOR), 94.91 and 96.58 (2 C, 2 x O*C*H_2_O), 110.11 (1 C, *C*Me_2_), 123.43 (1 C, Ph), 126.39 (2 C, Ph), 128.31 (1 C, Ph), 128.50 (2 C, Ph), 137.65 and 137.43 (2 C, *C*=*C*HPh); *m/z* (CI, NH_3_), 396 (M + NH_4_^+^), [Found: M + NH_4_^+^, 396.2378. C_21_H_34_O_6_N requires M + NH_4_^+^ 396.2376].

**(1*Z*,2*R*,3*S*,4*R*,5*R*,6*R*)-1-Benzylidene-3,4-(isopropylidenedioxy)-2,5-bis(methoxymethoxy)-6-methylcyclohexane (36):** *R_f_* = 0.56 in light petroleum/diethyl ether (4:6); [α]_D_ +28.6 (*c* 1.18 in CHCl_3_); ν_max_ (film)/cm^-1^ 3057, 3021, 2953, 2923, 2852, 2782, 2341, 1729, 1689, 1653, 1598, 1517, 1461, 1378, 1285, 1258, 1214, 1149, 1118, 1033, 989 and 914; δ_H_ (270 MHz) (with the help of 2D ^1^H NMR) 1.15 (3 H, d, *J* 6.6, CHMe), 1.38 (3 H, s, Me), 1.58 (3 H, s, Me), 3.15 (1 H, dq, *J* 3.4 and 6.6, CHC*H*Me), 3.39 (3 H, s, OMe), 3.43 (3 H, s, OMe), 3.92 (1 H, dd, *J* 3.4 and 5.9, CHC*H*(OMOM)CH), 4.28–4.39 (2 H, m, CHC*H*CH(OMOM)C= and CHC*H*CH(OMOM)CH), 4.52–4.63 (2 x 1H, d, *J* 6.6, OCH*H*O), 4.74 (2 x 1H, d, *J* 6.6, OC*H*HO), 4.76–4.81 (1 H, d, *J* 9.9, CHC*H*(OMOM)C=), 6.71 (1 H, s, C=C*H*Ph), 7.23–7.39 (5 H, m, Ph); δ_C_ (67.8 MHz) 14.19 (1 C, CH*Me*), 24.15 (1 C, Me), 25.44 (1 C, Me), 41.78 (1 C, *C*HMe), 55.64 and 55.69 (2 C, 2 x OMe), 71.79, 76.54, 76.09, and 77.44 (4 C, 4 x CHOR), 94.83 and 95.19 (2 C, 2 x O*C*H_2_O), 110.95 (1 C, *C*Me_2_), 126.78 (1 C, Ph), 127.29 (2 C, Ph), 128.37 (1 C, Ph), 128.34 (2 C, Ph), 137.67 and 137.74 (2 C, *C*=*C*HPh); *m/z* (CI, NH_3_), 396 (M + NH_4_^+^), [Found: M + NH_4_^+^, 396.2384. C_21_H_34_O_6_N requires M + NH_4_^+^ 396.2385].

**(1*E*,2*R*,3*S*,4*R*,5*R*,6*R*)-1-Benzylidene-3,4-(isopropylidenedioxy)-2,5-bis(methoxymethoxy)-6-methylcyclohexane (37):** *R_f_* = 0.11 in light petroleum/diethyl ether (4:6); [α]_D_ -23.5 (*c* 0.58 in CHCl_3_); ν_max_ (film)/cm^-1^ 3064, 3032, 2994, 2943, 2924, 2856, 2777, 2344, 1738, 1696, 1658, 1596, 1524, 1467, 1387, 1285, 1258, 1225, 1157, 1114, 1036, 997 and 914; δ_H_ (270 MHz) (with the help of 2D ^1^H NMR) 1.15 (3 H, d, *J* 6.6, CHMe), 1.36 (3 H, s, Me), 1.54 (3 H, s, Me), 3.2 (1 H, dq, *J* 4.2 and 6.6, CHC*H*Me), 3.41 (3 H, s, OMe), 3.46 (3 H, s, OMe), 3.91–3.98 (1 H, dd, *J* 4.2 and 5.9, CHC*H*(OMOM)CH), 4.27–4.37 (2 H, m, CHC*H*CH(OMOM)C= and CHC*H*CH(OMOM)CH), 4.53 (2 x 1H, d, *J* 6.6, OC*H*HO), 4.73 (2 x 1H, d, *J* 6.6, 2 x OC*H*HO), 4.80 (1 H, d, *J* 9.6, CHC*H*(OMOM)C=), 6.72 (1 H, s, C=C*H*Ph), 7.24–7.43 (5 H, m, Ph); δ_C_ (67.8 MHz) 14.31 (1 C, CH*Me*), 24.42 (1 C, Me), 25.58 (1 C, Me), 42.18 (1 C, *C*HMe), 55.18 and 55.19 (2 C, 2 x OMe), 71.27, 76.59, 76.76, and 77.75 (4 C, 4 x CHOR), 93.98 and 95.38 (2 C, 2 x O*C*H_2_O), 110.23 (1 C, *C*Me_2_), 126.76 (1 C, Ph), 127.38 (2 C, Ph), 128.37 (1 C, Ph), 128.49 (2 C, Ph), 137.73 and 138.12 (2 C, *C*=*C*HPh); *m/z* (CI, NH_3_), 396 (M + NH_4_^+^), [Found: M + NH_4_^+^, 396.2388. C_21_H_34_O_6_N requires M + NH_4_^+^ 396.2393].

**(2*S*,3*R*,4*R*,5*R*,6*R*)-3,4-(Isopropylidenedioxy)-2,5-bis(methoxymethoxy)-6-phenylcyclohexanone (38):** The alkenes **30** (0.09 g, 0.19 mmol) and **31** (0.045 g, 0.11 mmol) were dissolved in a mixture of DCM (5 ml) and methanol (5 ml). The mixture was cooled to −78 °C and ozone was passed for 40 min. The pale blue solution was purged with argon and dimethyl sulfide (2 ml) was added to destroy the ozonide. The reaction mixture was concentrated and the residue was dissolved in DCM (10 ml), washed with water (5 ml). The aqueous layer was further extracted with DCM (2 x 10 ml). The combined organic layers were dried over Na_2_SO_4_ and evaporated under reduced pressure. The residue was purified by flash chromatography with light petroleum/ethyl acetate (3:7) as eluent. The title compound was isolated as a colourless oil **38** (0.08 g, 77%).[α]_D_ +28.4 (*c* 0.48 in CHCl_3_); ν_max_(film)/cm^-1^ 3074, 3058, 3023, 2987, 2950, 2923, 2895, 2854, 2838, 1730 (C=O), 1598, 1494, 1455, 1391, 1378, 1253, 1216, 1148, 1107, 1075, 1030 and 918; δ_H_ (270 MHz) (with the help of 2D ^1^H NMR) 1.47 (3 H, s, Me), 1.68 (3 H, s, Me), 3.09 (3 H, s, OMe), 3.24 (3 H, s, OMe), 4.05 (1 H, d, *J* 6.67, OC*H*HO), 4.09 (1 H, d, *J* 10.4, CHC*H*(Ph)C=O), 4.09 (1 H, d, *J* 10.4, CHC*H*(OMOM)C=O), 4.36 (2 x 1H, d, *J* 6.3, OC*H*HO and OCH*H*O), 4.38 (1 H, dd, *J* 4.2 and 10.4, CHC*H*(OMOM)CH(Ph)C=), 4.57 (1 H, d, *J* 6.7, OCH*H*O), 4.67 (1 H, dd, *J* 4.7 and 10.2 CHC*H*CH(OMOM)C=), 4.73 (1 H, dd, *J* 4.4 and 6.6, C*H*OR), 7.26–7.39 (5 H, m, Ph); δ_C_ (67.8 MHz) 24.88 (1 C, Me), 25.21 (1 C, Me), 47.29 (1 C, C*H*Ph), 55.49 and 55.39 (2 C, 2 x OMe), 72.65, 74.84, 76.25, and 76.45 (4 C, 4 x CHOR), 93.29 and 96.78 (2 C, 2 x O*C*H_2_O), 111.09 (1 C, *C*Me_2_), 126.67 (1 C, Ph), 126.77 (1 C, Ph), 127.28 (2 C, Ph), 129.18 (1 C, Ph), and 136.68 (1 C, Ph), and 205.08 (1 C, *C*=O); *m/z* (CI, NH_3_), 384 (M + NH_4_^+^), [Found: M + NH_4_^+^, 384.2024 C_19_H_30_O_7_N requires M + NH_4_^+^ 384.2022].

**(2*S*,3*R*,4*R*,5*R*,6*S*)-3,4-(Isopropylidenedioxy)-2,5-bis(methoxymethoxy)-6-phenylcyclohexanone (39):** The alkenes **32** (0.07 g, 0.15 mmol) and **33** (0.04 g, 0.10 mmol) were dissolved in a mixture of DCM (5 ml) and methanol (5 ml). Ozonolysis, conducted as described above, gave the title compound **39** as a colourless oil (0.07 g, 74%). [α]_D_ −38.4 (*c* 0.35 in CHCl_3_); ν_max_ (film)/cm^-1^ 3069, 3059, 3021, 2986, 2953, 2925, 2895, 2851, 2838, 1729 (C=O), 1598, 1492, 1457, 1391, 1380, 1255, 1218, 1148, 1106, 1075, 1032 and 918; δ_H_ (270 MHz) (with the help of 2D ^1^H NMR) 1.45 (3 H, s, Me), 1.67 (3 H, s, Me), 3.08 (3 H, s, OMe), 3.22 (3 H, s, OMe), 4.06 (1 H, d, *J* 6.7, OC*H*HO), 4.14 (1 H, d, *J* 5.6, CHC*H*(Ph)C= ), 4.17 (1 H, d, *J* 9.8, CHC*H*(OMOM)C=), 4.31 (2 x 1H, d, *J* 6.5, OC*H*HO and OCH*H*O), 4.46 (1 H, dd, *J*4.1 and 5.5, CHC*H*(OMOM)CH(Ph)C=), 4.58 (1 H, d, *J* 6.8, OCH*H*O), 4.70 (1 H, dd, *J* 4.7 and 9.8 CHC*H*CH(OMOM)C=), 4.71 (1 H, dd, *J* 4.5 and 6.6, C*H*OR), 7.23–7.39 (5 H, m, Ph); δ_C_ (67.8 MHz) 25.07 (1 C, Me), 25.77 (1 C, Me), 48.29 (1 C, C*H*Ph), 55.49 and 55.70 (2 C, 2 x OMe), 72.81, 75.19, 76.29, and 76.48 (4 C, 4 x CHOR), 94.48 and 96.73 (2 C, 2 x O*C*H_2_O), 111.09 (1 C, *C*Me_2_), 126.67 (1 C, Ph), 126.77 (1 C, Ph), 127.28 (2 C, Ph),129.18 (1 C, Ph), 137.18 (1 C, Ph) and 205.24 (1 C, *C*=O); *m/z* (CI, NH_3_), 384 (M + NH_4_^+^), [Found: M + NH_4_^+^, 384.2026. C_19_H_30_O_7_N requires M + NH_4_^+^ 384.2022].

**(2*S*,3*R*,4*R*,5*R*,6*R*)-3,4-(Isopropylidenedioxy)-2,5-bis(methoxymethoxy)-6-methylcyclohexanone (40):** The alkenes **34** (70 mg, 0.19 mmol) and **35** (60 mg, 0.15 mmol) were dissolved in a mixture of DCM (5 ml) and methanol (5 ml) and cooled to −78 °C. Ozone was bubbled through the mixture at −78 °C for 40 min. The excess ozone was removed by bubbling argon through the solution. To the resultant pale blue solution dimethyl sulfide (2 ml) was added in order to decompose the ozonide. The reaction mixture was concentrated to afford a residual oil. The latter was dissolved in DCM (10 ml), washed with water (5 ml) and then the aqueous layer was back-extracted with DCM (2 x 10 ml). The organic layers were combined, dried over Na_2_SO_4_ and evaporated under reduced pressure. The residue was purified by flash chromatography with light petroleum/ethyl acetate (3:7) as eluent to afford compound **40** (0.08 g, 78%) as a colourless oil; *R_f_* = 0.21 in light petroleum/diethyl ether (1:1); [α]_D_ −47.6 (*c* 0.76 in CHCl_3_); ν_max_ (film)/cm^-1^ 3023, 2989, 2974, 2935, 2890, 2829, 2787, 2368, 2346, 1734 (C=O), 1628, 1459, 1378, 1274, 1214, 1153, 1095, 1037 and 918; δ_H_ (270 MHz) (with the help of 2D ^1^H NMR) 1.23 (3 H, d, *J* 7.2, CHMe), 1.39 (3 H, s, Me), 1.45 (3 H, s, Me), 2.74 (1 H, dq, *J* 10.9 and 7.2, CHC*H*Me), 3.39 (3 H, s, OMe), 3.44 (3 H, s, OMe), 3.76 (1 H, dd, *J* 2.6 and 10.9, CHC*H*(OMOM)CH), 4.1 (1 H, d, *J* 6.6, OC*H*HO), 4.27 (1 H, d, *J* 9.8, CHC*H*(OMOM)C=O), 4.39 (1 H, d, *J* 6.6, OC*H*HO), 4.54–4.63 (2 H, m, CHC*H*CH(OMOM)C=O and CHC*H*CH(OMOM)CH)), 4.68 (1 H, d, *J* 6.6 OCH*H*O), 4.72 (1 H, d, *J* 6.6, OCH*H*O); δ_C_ (67.8 MHz) 13.85 (1 C, CH*Me*), 23.61 (1 C, Me), 25.63 (1 C, Me), 43.89 (1 C, *C*HMe), 55.84 and 56.17 (2 C, 2 x OMe), 74.07, 74.47, 74.89, and 75.74 (4 C, 4 x CHOR), 95.89 and 96.23 (2 C, 2 x O*C*H_2_O), 110.31 (1 C, *C*Me_2_), 206.65 (1 C, *C*=O); *m/z* (CI, NH_3_), 322 (M + NH_4_^+^), [Found: M + NH_4_^+^, 322.1868. C_14_H_28_O_7_N requires M + NH_4_^+^ 322.1866].

**(2*S*,3*R*,4*R*,5*R*,6*S*)-3,4-(Isopropylidenedioxy)-2,5-bis(methoxymethoxy)-6-methylcyclohexanone (41):** The alkenes **36** (40 mg, 0.12 mmol) and **37** (30 mg, 0.09 mmol) were dissolved in a mixture of DCM (5 ml) and methanol (5 ml). Ozonolysis, conducted as described above, allowed the title compound **41** to be obtained as a colourless oil (0.05 g, 74%); *R_f_* = 0.19 in light petroleum/diethyl ether (1:1); [α]_D_ +24.8 (*c* 0.67 in CHCl_3_);ν_max_ (film)/cm^-1^ 3037, 3002, 2964, 2935, 2891, 2824, 2773, 2364, 2347, 1735 (C=O), 1628, 1457, 1383, 1276, 1217, 1154, 1096, 1038 and 923; δ_H_ (270 MHz) (with the help of 2D ^1^H NMR) 1.26 (3 H, d, *J* 6.2, CHMe), 1.39 (3 H, s, Me), 1.41 (3 H, s, Me), 2.75 (1 H, dq, *J* 4.7 and 6.3, CHC*H*Me), 3.41 (3 H, s, OMe), 3.45 (3 H, s, OMe), 3.76 (1 H, dd, *J* 2.6 and 4.7, CHC*H*(OMOM)CH), 4.11 (1 H, d, *J* 6.6, OC*H*HO), 4.28 (1 H, d, *J* 9.7, CHC*H*(OMOM)C=O), 4.41 (1 H, d, *J* 6.6, OC*H*HO), 4.54–4.63 (2 H, m, CHC*H*CH(OMOM)C=O and CHC*H*CH(OMOM)CH), 4.67 (1 H, d, *J* 6.6 OCH*H*O), 4.72 (1 H, d, *J* 6.6, OCH*H*O); δ_C_ (67.8 MHz) 13.76 (1 C, CH*Me*), 23.75 (1 C, Me), 25.48 (1 C, Me), 44.05 (1 C, *C*HMe), 55.37 and 56.07 (2 C, 2 x OMe), 73.89, 74.74, 74.49, and 75.74 (4 C, 4 x CHOR), 95.79 and 96.57 (2 C, 2 x O*C*H_2_O), 110.84 (1 C, *C*Me_2_), 206.11 (1 C,*C*=O); *m/z* (CI, NH_3_), 322 (M + NH_4_^+^), [Found: M + NH_4_^+^, 322.1860. C_14_H_28_O_7_N requires M + NH_4_^+^ 322.1866].

**(1*Z*,2*S*,3*S*,4*R*,5*R*,6*S*)-1-Benzylidene-3,4-(isopropylidenedioxy)-2,5-bis(methoxymethoxy)-6-phenylcyclohexane (42), (1*E*,2*S*,3*S*,4*R*,5*R*,6*S*)-1-benzylidene-3,4-(isopropylidenedioxy)-2,5-bis(methoxymethoxy)-6-phenylcyclohexane** **(43), (1*Z*,2*S*,3*S*,4*R*,5*R*,6*R*)-1-benzylidene-3,4-(isopropylidenedioxy)-2,5-bis(methoxymethoxy)-6-phenylcyclohexane** **(44), (1*E*,2*S*,3*S*,4*R*,5*R*,6*R*)-1-benzylidene-3,4-(isopropylidenedioxy)-2,5-bis(methoxymethoxy)-6-phenylcyclohexane** **(45):** The iodides **26** (0.60 g, 1.06 mmol) were dissolved in dry benzene (50 ml) and the solution was degassed with argon for 40 min. The solution was heated to reflux at 80 °C and a catalytic amount of AIBN was added followed by tributyltin hydride (0.34 g, 0.31 ml, 1.16 mmol). After 3 h at reflux the reaction mixture was cooled to RT and concentrated to afford a residual oil. The product was dissolved in acetonitrile (100 ml), washed with light petroleum (40 ml). The petroleum layer was back-extracted with acetonitrile (2 x 100 ml). The acetonitrile extracts were combined and evaporated under reduced pressure. The residue was purified by flash chromatography with light petroleum/diethyl ether (3:1 to 1:1) as eluent to obtain the title compounds as a colourless oils **42** (0.15 g, 29%), **43** (0.08 g, 17%), **44** (0.11 g, 23%) and **45** (0.07 g, 14%).

**(1*Z*,2*S*,3*S*,4*R*,5*R*,6*S*)-1-Benzylidene-3,4-(isopropylidenedioxy)-2,5-bis(methoxymethoxy)-6-phenylcyclohexane (42):** [α]_D_ −48.6(*c* 1.05 in CHCl_3_); ν_max_(film)/cm^-1^ 3073, 3059, 3022, 2985, 2953, 2923, 2895, 2853, 2839, 1735, 1599, 1495, 1457, 1391, 1378, 1253, 1218, 1149, 1109, 1077, 1032 and 918; δ_H_ (270 MHz) (with the help of 2D ^1^H NMR) 1.48 (3 H, s, Me), 1.67 (3 H, s, Me), 3.08 (3 H, s, OMe), 3.23 (3 H, s, OMe), 4.06 (1 H, d, *J* 6.7, OC*H*HO), 4.12 (1 H, d, *J* 9.6, CHC*H*(Ph)C=), ), 4.14 (1 H, d, *J* 4.3, CHC*H*(OMOM)C=), 4.33 (2 x 1H, d, *J* 6.5, OC*H*HO and OCH*H*O), 4.47 (1 H, dd, *J* 4.7 and 9.6, CHC*H*(OMOM)CH(Ph)C=), 4.58 (1 H, d, *J* 6.8, OCH*H*O), 4.67 (1 H, dd, *J* 4.3 and 6.6 CHC*H*CH(OMOM)C=), 4.70 (1 H, dd, *J* 4.3 and 6.6, C*H*OR), 6.45 (1 H, s, C=C*H*Ph), 7.17–7.44 (10 H, m, Ph); δ_C_ (67.8 MHz) 25.08 (1 C, Me), 25.72 (1 C, Me), 48.27 (1 C, C*H*Ph), 55.46 and 55.67 (2 C, 2 x OMe), 72.58, 75.02, 76.25, and 76.45 (4 C, 4 x CHOR), 94.27 and 96.45 (2 C, 2 x O*C*H_2_O), 110.39 (1 C, *C*Me_2_), 126.67 (1 C, Ph), 126.77 (1 C, Ph), 127.28 (2 C, Ph), 128.51 (2 C, Ph), 128.89 (2 C, Ph), 129.18 (1 C, Ph), 131.73 (2 C, Ph) and 136.66 (1 C, Ph), 137.32 and 141.77 (2 C, *C*=*C*HPh); *m/z* (CI, NH_3_) 458 (M + NH_4_^+^), [Found: M + NH_4_^+^, 458.2542. C_26_H_36_O_6_N requires M + NH_4_^+^ 458.2543].

**(1*E*,2*S*,3*S*,4*R*,5*R*,6*S*)-1-Benzylidene-3,4-(isopropylidenedioxy)-2,5-bis(methoxymethoxy)-6-phenylcyclohexane (43):** [α]_D_+28.6(*c* 0.85 in CHCl_3_); ν_max_(film)/cm^-1^ 3073, 3059, 3022, 2985, 2953, 2923, 2895, 2853, 2839, 1735, 1599, 1495, 1457, 1391, 1378, 1253, 1218, 1149, 1109, 1077, 1032 and 918; δ_H_ (270 MHz) (with the help of 2D ^1^H NMR) 1.48 (3 H, s, Me), 1.67 (3 H, s, Me), 3.08 (3 H, s, OMe), 3.23 (3 H, s, OMe), 4.06 (1 H, d, *J* 6.7, OC*H*HO), 4.11 (1 H, d, *J* 9.8, CHC*H*(Ph)C=), 4.13 (1 H, d, *J* 4.3, CHC*H*(OMOM)C=), 4.33 (2H, d, *J* 6.5, OC*H*HO and OCH*H*O), 4.47 (1 H, dd, *J* 4.7 and 9.8, CHC*H*(OMOM)CH(Ph)C=), 4.58 (1 H, d, *J* 6.8, OCH*H*O), 4.67 (1 H, dd, *J* 4.3 and 6.6 CHC*H*CH(OMOM)C=), 4.70 (1 H, dd, *J* 4.3 and 6.6, C*H*OR), 6.43 (1 H, s, C=C*H*Ph), 7.17–7.44 (10 H, m, Ph); δ_C_ (67.8 MHz) 25.08 (1 C, Me), 25.72 (1 C, Me), 48.27 (1 C, C*H*Ph), 55.46 and 55.67 (2 C, 2 x OMe), 72.58, 75.02, 76.25, and 76.45 (4 C, 4 x CHOR), 94.27 and 96.45 (2 C, 2 x O*C*H_2_O), 110.39 (1 C, *C*Me_2_), 126.67 (1 C, Ph), 126.77 (1 C, Ph), 127.28 (2 C, Ph), 128.51 (2 C, Ph), 128.89 (2 C, Ph), 129.18 (1 C, Ph), 131.73 (2 C, Ph) and 136.66 (1 C, Ph), 137.32 and 141.77 (2 C, *C*=*C*HPh); *m/z* (CI, NH_3_) 458 (M + NH_4_^+^), [Found: M + NH_4_^+^, 458.2581. C_26_H_36_O_6_N requires M + NH_4_^+^ 458.2582].

**(1*Z*,2*S*,3*S*,4*R*,5*R*,6*R*)-1-Benzylidene-3,4-(isopropylidenedioxy)-2,5-bis(methoxymethoxy)-6-phenylcyclohexane (44):** [α]_D_ −47.6(*c* 1.08 in CHCl_3_); ν_max_(film)/cm^-1^ 3070, 3059, 3022, 2985, 2953, 2921, 2895, 2853, 2839, 1735, 1599, 1495, 1459, 1391, 1378, 1252, 1219, 1148, 1109, 1077, 1032 and 918; δ_H_ (270 MHz) (with the help of 2D ^1^H NMR) 1.47 (3 H, s, Me), 1.69 (3 H, s, Me), 3.07 (3 H, s, OMe), 3.24 (3 H, s, OMe), 4.07 (1 H, d, *J* 6.7, OC*H*HO), 4.10 (1 H, d, *J* 5.2, CHC*H*(Ph)C=), 4.12 (1 H, d, *J* 4.3, CHC*H*(OMOM)C=), 4.31 (2 x 1H, d, *J* 6.5, OC*H*HO and OCH*H*O), 4.47 (1 H, dd, *J* 3.3 and 5.6, CHC*H*(OMOM)CH(Ph)C=), 4.59 (1 H, d, *J* 6.8, OCH*H*O), 4.67 (1 H, dd, *J* 3.4 and 6.6 CHC*H*CH(OMOM)C=), 4.67 (1 H, dd, *J* 4.3 and 6.6, C*H*OR), 6.43 (1 H, s, C=C*H*Ph), 7.14–7.39 (10 H, m, Ph); δ_C_ (67.8 MHz) 25.12 (1 C, Me), 25.69 (1 C, Me), 48.32 (1 C, C*H*Ph), 55.49 and 55.73 (2 C, 2 x OMe), 72.68, 75.12, 76.29, and 76.57 (4 C, 4 x CHOR), 94.78 and 96.89 (2 C, 2 x O*C*H_2_O), 110.09 (1 C, *C*Me_2_), 126.69 (1 C, Ph), 126.73 (1 C, Ph), 127.22 (2 C, Ph), 128.58 (2 C, Ph), 128.89 (2 C, Ph), 129.18 (1 C, Ph), 131.74 (2 C, Ph) and 136.66 (1 C, Ph), 137.33 and 141.76 (2 C, *C*=*C*HPh); *m/z* (CI, NH_3_) 458 (M + NH_4_^+^), [Found: M + NH_4_^+^, 458.2584. C_26_H_36_O_6_N requires M + NH_4_^+^ 458.2585].

**(1*E*,2*S*,3*S*,4*R*,5*R*,6*R*)-1-Benzylidene-3,4-(isopropylidenedioxy)-2,5-bis(methoxymethoxy)-6-phenylcyclohexane (45):** [α]_D_ +32.8(*c* 0.65 in CHCl_3_); ν_max_(film)/cm^-1^ 3070, 3059, 3022, 2985, 2953, 2921, 2895, 2853, 2839, 1735, 1599, 1495, 1459, 1391, 1378, 1252, 1219, 1148, 1109, 1077, 1032 and 918; δ_H_ (270 MHz) (with the help of 2D ^1^H NMR) 1.47 (3 H, s, Me), 1.69 (3 H, s, Me), 3.07 (3 H, s, OMe), 3.24 (3 H, s, OMe), 4.07 (1 H, d, *J* 6.7, OC*H*HO), 4.10 (1 H, d, *J* 5.1, CHC*H*(Ph)C=), 4.12 (1 H, d, *J* 4.3, CHC*H*(OMOM)C=), 4.31 (2 x 1H, d, *J* 6.5, OC*H*HO and OCH*H*O), 4.47 (1 H, dd, *J* 3.3 and 5.6, CHC*H*(OMOM)CH(Ph)C=), 4.59 (1 H, d, *J* 6.8, OCH*H*O), 4.67 (1 H, dd, *J* 4.3 and 6.6 CHC*H*CH(OMOM)C=), 4.67 (1 H, dd, *J* 4.3 and 6.6, C*H*OR), 6.43 (1 H, s, C=C*H*Ph), 7.14–7.39 (10 H, m, Ph); δ_C_ (67.8 MHz) 25.12 (1 C, Me), 25.69 (1 C, Me), 48.32 (1 C, C*H*Ph), 55.49 and 55.73 (2 C, 2 x OMe), 72.68, 75.12, 76.29, and 76.57 (4 C, 4 x CHOR), 94.78 and 96.89 (2 C, 2 x O*C*H_2_O), 110.09 (1 C, *C*Me_2_), 126.69 (1 C, Ph), 126.73 (1 C, Ph), 127.22 (2 C, Ph), 128.58 (2 C, Ph), 128.89 (2 C, Ph), 129.18 (1 C, Ph), 131.74 (2 C, Ph) and 136.66 (1 C, Ph), 137.33 and 141.76 (2 C, *C*=*C*HPh); *m/z* (CI, NH_3_) 458 (M + NH_4_^+^), [Found: M + NH_4_^+^, 458.2543. C_26_H_36_O_6_N requires M + NH_4_^+^ 458.2543].

**(1*Z*,2*S*,3*S*,4*R*,5*R*,6*S*)-1-Benzylidene-3,4-(isopropylidenedioxy)-2,5-bis(methoxymethoxy)-6-methylcyclohexane (46), (1*E*,2*S*,3*S*,4*R*,5*R*,6*S*)-1-benzylidene-3,4-(isopropylidenedioxy)-2,5-bis(methoxymethoxy)-6-methylcyclohexane (47), (1*Z*,2*S*,3*S*,4*R*,5*R*,6*R*)-1-benzylidene-3,4-(isopropylidenedioxy)-2,5-bis(methoxymethoxy)-6-methylcyclohexane (48), (1*E*,2*S*,3*S*,4*R*,5*R*,6*R*)-1-benzylidene-3,4-(isopropylidenedioxy)-2,5-bis(methoxymethoxy)-6-methylcyclohexane (49):** The iodides **28** (0.85 g, 1.68 mmol) were dissolved in dry benzene (50 ml) and the solution was degassed by bubbling argon through the solution for 40 min. The solution was heated to reflux and a catalytic amount of AIBN was added. Tributyltin hydride (0.54 g, 0.50 ml, 1.85 mmol) was added dropwise to the reaction mixture over 30 min. After a further 3 h under reflux the reaction mixture was concentrated to afford a residue. The latter was dissolved in acetonitrile (100 ml), washed with light petroleum (40 ml). The petroleum layer was extracted with acetonitrile (2 x 100 ml). The layers of acetonitrile were combined and evaporated under reduced pressure. The residue was purified by flash chromatography with light petroleum/diethyl ether (4:1 to 1:1) as eluent to obtain the title compounds as colourless oils **46** (0.21 g, 33%), **47** (0.12 g, 19%), **48** (0.17 g, 26%) and **49** (0.09 g, 14%).

**(1*Z*,2*S*,3*S*,4*R*,5*R*,6*S*)-1-Benzylidene-3,4-(isopropylidenedioxy)-2,5-bis(methoxymethoxy)-6-methylcyclohexane (46):** *R_f_* = 0.68 in light petroleum/diethyl ether (4:6); [α]_D_ −46.9 (*c* 2.48 in CHCl_3_); ν_max_ (film)/cm^-1^ 3058, 3021, 2983, 2931, 2854, 2825, 2780, 2252, 1725, 1600, 1492, 1461, 1378, 1247, 1214, 1147, 1101, 1070, 1033 and 998; δ_H_ (270 MHz) (with the help of 2D ^1^H NMR) 1.27 (3 H, d, *J* 6.6, CHMe), 1.40 (3 H, s, Me), 1.62 (3 H, s, Me), 3.06 (1 H, dq, *J* 10.5 and 6.6, CHC*H*Me), 3.22 (3 H, s, OMe), 3.46 (3 H, s, OMe), 4.20–4.25 (2 H, m, CHC*H*CH(OMOM)C= and CHC*H*CH(OMOM)CH), 4.25 (1 H, d, *J* 6.6, OC*H*HO), 4.4 (1 H, d, *J* 6.6, OC*H*HO), 4.54 (1 H, d, *J* 4.6, CHC*H*(OMOM)C=), 4.61 (1 H, dd, *J* 4.0 and 5.9 CHC*H*(OMOM)CH), 4.74 (1 H, d, *J* 6.3, OCH*H*O), 4.86 (1 H, d, *J* 6.6, OCH*H*O), 6.60 (1 H, s, C=C*H*Ph), 7.17–7.31 (5 H, m, Ph); δ_C_ (67.8 MHz) 15.40 (1 C, CH*Me*), 25.39 (1 C, Me), 25.60 (1 C, Me), 40.77 (1 C, *C*HMe), 55.30 and 55.67 (2 C, 2 x OMe), 70.65, 74.73, 76.02, and 79.71 (4 C, 4 x CHOR), 94.31 and 96.59 (2 C, 2 x O*C*H_2_O), 110.10 (1 C, *C*Me_2_), 126.74 (1 C, Ph), 127.88 (2 C, Ph), 128.08 (1 C, Ph), 128.56 (2 C, Ph), 136.64 and 139.11 (2 C, *C*=*C*HPh); *m/z* (CI, NH_3_), 396 (M + NH_4_^+^), [Found: M + NH_4_^+^, 396.2386. C_21_H_34_O_6_N requires M + NH_4_^+^ 396.2386].

**(1*E*,2*S*,3*S*,4*R*,5*R*,6*S*)-1-Benzylidene-3,4-(isopropylidenedioxy)-2,5-bis(methoxymethoxy)-6-methylcyclohexane (47):** *R_f_* = 0.40 in light petroleum/diethyl ether (4:6); [α]_D_ +46.9 (*c* 2.48 in CHCl_3_); ν_max_ (film)/cm^-1^ 3055, 3018, 2954, 2987, 2873, 2854, 2778, 2358, 2341, 1725, 1652, 1598, 1494, 1461, 1380, 1249, 1214, 1151, 1103, 1037, 948 and 916; δ_H_ (270 MHz) (with the help of 2D ^1^H NMR) 1.14 (3 H, d, *J* 5.3, CHMe), 1.35 (3 H, s, Me), 1.43 (3 H, s, Me), 3.17 (1 H, dq, *J* 9.3 and 5.3, CHC*H*Me), 3.44 (3 H, s, OMe), 3.46 (3 H, s, OMe), 3.62 (1 H, dd, *J* 2.6 and 9.2, CHC*H*(OMOM)CHMe), 4.24–4.28 (1 H, m, CHC*H*CH(OMOM)CH), 4.31 (1 H, d, *J* 6.6, OC*H*HO), 4.56 (1 H, dd, *J* 4.0 and 7.3, CHC*H*CH(OMOM)C=), 4.71 (1 H, d, *J* 7.2, OC*H*HO), 4.76 (1 H, d, *J* 3.3, CHC*H*(OMOM)C=), 4.79 (1 H, d, *J* 7.3, OCH*H*O), 4.82 (1 H, d, *J* 7.3, OCH*H*O), 6.50 (1 H, s, C=C*H*Ph), 7.19–7.38 (5 H, m, Ph); δ_C_ (67.8 MHz) 17.87 (1 C, CH*Me*), 24.42 (1 C, Me), 25.78 (1 C, Me), 34.54 (1 C, *C*HMe), 55.64 and 55.68 (2 C, 2 x OMe), 72.28, 75.04, 77.47, and 77.94 (4 C, 4 x CHOR), 94.95 and 96.50 (2 C, 2 x O*C*H_2_O), 110.11 (1 C, *C*Me_2_), 123.43 (1 C, Ph), 126.39 (2 C, Ph), 128.31 (1 C, Ph), 128.50 (2 C, Ph), 137.00 and 137.38 (2 C, *C*=*C*HPh); *m/z* (CI, NH_3_), 396 (M + NH_4_^+^), [Found: M + NH_4_^+^, 496.2384. C_21_H_34_O_6_N requires M + NH_4_^+^ 396.2388].

**(1*Z*,2*S*,3*S*,4*R*,5*R*,6*R*)-1-Benzylidene-3,4-(isopropylidenedioxy)-2,5-bis(methoxymethoxy)-6-methylcyclohexane (48):** *R_f_* = 0.52 in light petroleum/diethyl ether (4:6); [α]_D_ −24.3 (*c* 0.97 in CHCl_3_); ν_max_ (film)/cm^-1^ 3056, 3022, 2954, 2923, 2852, 2782, 2341, 1729, 1691, 1650, 1598, 1515, 1461, 1378, 1282, 1255, 1214, 1149, 1108, 1033, 995 and 917; δ_H_ (270 MHz) (with the help of 2D ^1^H NMR) 1.15 (3 H, d, *J* 6.6, CHMe), 1.38 (3 H, s, Me), 1.58 (3 H, s, Me), 3.16 (1 H, dq, *J* 3.3 and 6.6, CHC*H*Me), 3.41 (3 H, s, OMe), 3.45 (3 H, s, OMe), 3.94 (1 H, dd, *J* 3.3 and 5.9, CHC*H*(OMOM)CH), 4.28–4.37 (2 H, m, CHC*H*CH(OMOM)C= and CHC*H*CH(OMOM)CH), 4.56 (2 x 1H, d, *J* 6.6, OC*H*HO), 4.74 (2 x 1H, d, *J* 6.6, OC*H*HO), 4.77 (1 H, d, *J* 4.6, CHC*H*(OMOM)C=), 6.72 (1 H, s, C=C*H*Ph), 7.21–7.35 (5 H, m, Ph); δ_C_ (67.8 MHz) 14.21 (1 C, CH*Me*), 24.10 (1 C, Me), 25.48 (1 C, Me), 41.76 (1 C, *C*HMe), 55.62 and 55.71 (2 C, 2 x OMe), 71.79, 76.53, 76.08, and 77.47 (4 C, 4 x CHOR), 94.83 and 95.18 (2 C, 2 x O*C*H_2_O), 110.11 (1 C, *C*Me_2_), 126.70 (1 C, Ph), 127.27 (2 C, Ph), 128.30 (1 C, Ph), 128.40 (2 C, Ph), 137.69 and 137.89 (2 C, *C*=*C*HPh); *m/z* (CI, NH_3_), 396 (M + NH_4_^+^), [Found: M + NH_4_^+^, 496.2377. C_21_H_34_O_6_N requires M + NH_4_^+^ 396.2379].

**(1*E*,2*S*,3*S*,4*R*,5*R*,6*R*)-1-Benzylidene-3,4-(isopropylidenedioxy)-2,5-bis(methoxymethoxy)-6-methylcyclohexane (49):** *R_f_* = 0.08 in light petroleum/diethyl ether (4:6); [α]_D_ +18.4 (*c* 0.65 in CHCl_3_); ν_max_ (film)/cm^-1^ 3058, 3025, 2989, 2944, 2923, 2855, 2776, 2344, 1734, 1689, 1650, 1595, 1515, 1464, 1377, 1284, 1255, 1214, 1149, 1114, 1031, 990 and 916; δ_H_ (270 MHz) (with the help of 2D ^1^H NMR) 1.14 (3 H, d, *J* 6.7, CHMe), 1.37 (3 H, s, Me), 1.55 (3 H, s, Me), 3.2 (1 H, dq, *J* 3.8 and 6.6, CHC*H*Me), 3.42 (3 H, s, OMe), 3.47 (3 H, s, OMe), 3.94 (1 H, dd, *J* 3.7 and 5.9, CHC*H*(OMOM)CH), 4.25–4.34 (2 H, m, CHC*H*CH(OMOM)C= and CHC*H*CH(OMOM)CH), 4.53 (2 x 1H, d, *J* 6.6, OC*H*HO), 4.75 (2 x 1H, d, *J* 6.6, OC*H*HO), 4.80 (1 H, d, *J* 4.6, CHC*H*(OMOM)C=), 6.74 (1 H, s, C=C*H*Ph), 7.23–7.41 (5 H, m, Ph); δ_C_ (67.8 MHz) 14.29 (1 C, CH*Me*), 24.13 (1 C, Me), 25.68 (1 C, Me), 41.86 (1 C, *C*HMe), 55.68 and 55.76 (2 C, 2 x OMe), 71.82, 76.65, 76.88, and 77.87 (4 C, 4 x CHOR), 94.58 and 95.26 (2 C, 2 x O*C*H_2_O), 110.17 (1 C, *C*Me_2_), 126.75 (1 C, Ph), 127.28 (2 C, Ph), 128.35 (1 C, Ph), 128.44 (2 C, Ph), 137.70 and 137.93 (2 C, *C*=*C*HPh); *m/z* (CI, NH_3_), 396 (M + NH_4_^+^), [Found: M + NH_4_^+^, 396.2384. C_21_H_34_O_6_N requires M + NH_4_^+^ 396.2386].

**(2*R*,3*R*,4*R*,5*R*,6*R*)-3,4-(Isopropylidenedioxy)-2,5-bis(methoxymethoxy)-6-phenylcyclohexanone (50):** The alkenes **42** (0.13 g, 0.29 mmol) and **43** (0.07 g, 0.16 mmol) were dissolved in a mixture of DCM (5 ml) and methanol (5 ml) and cooled to −78 °C. Ozonolysis of the resultant solution was carried out for 40 min at which point a persistent blue colour was observed. The excess ozone was then removed by bubbling argon through the solution. The ozonide was destroyed *via* the addition of dimethyl sulfide (2 ml). The reaction mixture was concentrated to leave the residue, which was dissolved in DCM (10 ml), washed with water (5 ml). The aqueous layer was further extracted with DCM (2 x 10 ml). The organic layers were combined, dried over Na_2_SO_4_ and evaporated under reduced pressure. The residue was purified by flash chromatography with light petroleum/ethyl acetate (3:7) as eluent, to give **50** as a colourless oil (0.13 g, 76%). [α]_D_ +14.8 (*c* 0.6 in CHCl_3_); ν_max_(film)/cm^-1^ 3073, 3059, 3022, 2985, 2953, 2923, 2895, 2853, 2839, 1739 (C=O), 1599, 1495, 1457, 1391, 1378, 1253, 1218, 1149, 1109, 1077, 1032 and 919; δ_H_ (270 MHz) (with the help of 2D ^1^H NMR) 1.46 (3 H, s, Me), 1.65 (3 H, s, Me), 3.09 (3 H, s, OMe), 3.22 (3 H, s, OMe), 4.06 (1 H, d, *J* 6.64, OC*H*HO), 4.12 (1 H, d, *J* 9.7, CHC*H*(Ph)C=), 4.13 (1 H, d, *J* 4.3, CHC*H*(OMOM)C=), 4.32 (2 x 1H, d, *J* 6.5, OC*H*HO and OCH*H*O), 4.47 (1 H, dd, *J* 4.7 and 9.6, CHC*H*(OMOM)CH(Ph)C=), 4.58 (1 H, d, *J* 6.8, OCH*H*O), 4.67 (1 H, dd, *J* 4.3 and 6.6 CHC*H*CH(OMOM)C=), 4.70 (1 H, dd, *J* 4.3 and 6.6, C*H*OR), 7.27–7.39 (5 H, m, Ph); δ_C_ (67.8 MHz) 25.08 (1 C, Me), 25.72 (1 C, Me), 48.27 (1 C, C*H*Ph), 55.49 and 55.77 (2 C, 2 x OMe), 72.58, 75.02, 76.25, and 76.45 (4 C, 4 x CHOR), 94.25 and 96.38 (2 C, 2 x O*C*H_2_O), 110.57 (1 C, *C*Me_2_), 126.67 (1 C, Ph), 126.77 (1 C, Ph), 127.28 (2 C, Ph), 129.18 (1 C, Ph), 136.66 (1 C, Ph), and 203.14 (1 C, *C*=O); *m/z* (CI, NH_3_), 384 (M + NH_4_^+^), [Found: M + NH_4_^+^, 384.2024. C_19_H_30_O_7_N requires M + NH_4_^+^ 384.2022].

**(2*R*,3*R*,4*R*,5*R*,6*S*)-3,4-(Isopropylidenedioxy)-2,5-bis(methoxymethoxy)-6-phenylcyclohexanone (51):** The alkenes **44** (0.10 g, 0.22 mmol) and **45** (0.05 g, 0.12 mmol) were dissolved in a mixture of DCM (5 ml) and methanol (5 ml). Application of the procedure used for the preparation of **50** enabled the title compound **51** to be obtained as a colourless oil (0.10 g, 78%). [α]_D_ −12.4(*c* 0.5 in CHCl_3_); ν_max_(film)/cm^-1^ 3070, 3059, 3022, 2985, 2953, 2921, 2895, 2853, 2839, 1737 (C=O), 1599, 1495, 1391, 1378, 1252, 1217, 1148, 1077, 1032 and 918; δ_H_ (270 MHz) (with the help of 2D ^1^H NMR) 1.49 (3 H, s, Me), 1.72 (3 H, s, Me), 3.05 (3 H, s, OMe), 3.23 (3 H, s, OMe), 4.09 (1 H, d, *J* 6.7, OC*H*HO), 4.11 (1 H, d, *J* 5.1, CHC*H*(Ph)C=), 4.14 (1 H, d, *J* 4.33, CHC*H*(OMOM)C=), 4.33 (2 x 1H, d, *J* 6.5, OC*H*HO and OCH*H*O), 4.47 (1 H, dd, *J* 3.3 and 5.4, CHC*H*(OMOM)CH(Ph)C=), 4.61 (1 H, d, *J* 6.7, OCH*H*O), 4.66 (1 H, dd, *J* 4.3 and 6.6 CHC*H*CH(OMOM)C=), 4.70 (1 H, dd, *J* 4.3 and 6.6, C*H*OR), 7.27–7.39 (5 H, m, Ph); δ_C_ (67.8 MHz) 25.12 (1 C, Me), 25.69 (1 C, Me), 48.32 (1 C, C*H*Ph), 55.49 and 55.73 (2 C, 2 x OMe), 72.68, 75.12, 76.29, and 76.57 (4 C, 4 x CHOR), 94.78 and 96.89 (2 C, 2 x O*C*H_2_O), 110.79 (1 C, *C*Me_2_), 126.69 (1 C, Ph), 126.73 (1 C, Ph), 127.22 (2 C, Ph), 131.74 (1 C, Ph) and 136.66 (1 C, Ph), and 204.67(1 C, *C*=O); *m/z* (CI, NH_3_), 384 (M + NH_4_^+^), [Found: M + NH_4_^+^, 384.2744. C_19_H_30_O_7_N requires M + NH_4_^+^ 384.2746].

**(2*R*,3*R*,4*R*,5*R*,6*R*)-3,4-(Isopropylidenedioxy)-2,5-bis(methoxymethoxy)-6-methylcyclohexanone (52):** The alkenes **46** (0.19 g, 0.49 mmol) and **47** (0.14 g, 0.39 mmol) were dissolved in a mixture of DCM (5 ml) and methanol (5 ml). Employing the same procedure as that used for the preparation of **50** allowed the title compound to be obtained as a colourless oil (0.19 g, 71%); *R_f_* = 0.2 in light petroleum/diethyl ether (1:1); [α]_D_ +55.8 (*c* 0.43 in CHCl_3_); ν_max_(film)/cm^-1^ 3019, 2994, 2973, 2935, 2900, 2827, 2778, 2360, 2341, 1737 (C=O), 1623, 1455, 1384, 1276, 1214, 1153, 1095, 1033 and 917; δ_H_ (270 MHz) (with the help of 2D ^1^H NMR) 1.24 (3 H, d, *J* 7.3, CHMe), 1.37 (3 H, s, Me), 1.44 (3 H, s, Me), 2.72 (1 H, dq, *J* 11.2 and 7.3, CHC*H*Me), 3.41 (3 H, s, OMe), 3.45 (3 H, s, OMe), 3.78 (1 H, dd, *J* 2.6 and 11.2, CHC*H*(OMOM)CH), 4.1 (1 H, d, *J* 6.6, OC*H*HO), 4.27 (1 H, d, *J* 3.3, CHC*H*(OMOM)C=O), 4.41 (1 H, d, *J* 6.6, OC*H*HO), 4.53–4.62 (2 H, m, CHC*H*CH(OMOM)C=O and CHC*H*CH(OMOM)CH), 4.69 (1 H, d, *J* 6.6 OCH*H*O), 4.74 (1 H, d, *J* 6.6, OCH*H*O); δ_C_ (67.8 MHz) 13.88 (1 C, CH*Me*), 23.64 (1 C, Me), 25.60 (1 C, Me), 43.91 (1 C, *C*HMe), 55.86 and 55.97 (2 C, 2 x OMe), 73.87, 74.41, 74.84, and 75.65 (4 C, 4 x CHOR), 95.94 and 96.12 (2 C, 2 x O*C*H_2_O), 110.08 (1 C, *C*Me_2_), 206.07 (1 C, *C*=O); *m/z* (CI, NH_3_), 322 (M + NH_4_^+^), [Found: M + NH_4_^+^, 322.1866. C_14_H_28_O_7_N requires M + NH_4_^+^ 322.1866].

**(2*R*,3*R*,4*R*,5*R*,6*S*)-3,4-(Isopropylidenedioxy)-2,5-bis(methoxymethoxy)-6-methylcyclohexanone (53):** The alkenes **48** (0.12 g, 0.30 mmol) and **49** (0.07 g, 0.20 mmol) were dissolved in a mixture of DCM (5 ml) and methanol (5 ml). Employing the same procedure as that used for the preparation of **50** allowed the title compound **53** to be obtained as a colourless oil (0.12 g, 76%); *R_f_* = 0.17 in light petroleum/diethyl ether (1:1); [α]_D_ −27.3(*c* 0.68 in CHCl_3_); ν_max_(film)/cm^-1^ 3018, 2996, 2973, 2937, 2892, 2823, 2775, 2363, 2342, 1735 (C=O), 1626, 1454, 1387, 1274, 1216, 1154, 1096, 1038 and 921; δ_H_ (270 MHz) (with the help of 2D ^1^H NMR) 1.25 (3 H, d, *J* 6.2, CHMe), 1.38 (3 H, s, Me), 1.43 (3 H, s, Me), 2.69 (1 H, dq, *J* 4.6 and 6.2, CHC*H*Me), 3.41 (3 H, s, OMe), 3.45 (3 H, s, OMe), 3.76 (1 H, dd, *J* 2.6 and 4.6, CHC*H*(OMOM)CH), 4.11 (1 H, d, *J* 6.6, OC*H*HO), 4.28 (1 H, d, *J* 3.5, CHC*H*(OMOM)C=O), 4.41 (1 H, d, *J* 6.6, OC*H*HO), 4.54–4.63 (2 H, m, CHC*H*CH(OMOM)C=O and CHC*H*CH(OMOM)CH), 4.67 (1 H, d, *J* 6.6 OCH*H*O), 4.73 (1 H, d, *J* 6.6, OCH*H*O); δ_C_ (67.8 MHz) 13.87 (1 C, CH*Me*), 23.58 (1 C, Me), 25.64 (1 C, Me), 43.95 (1 C, *C*HMe), 55.83 and 55.97 (2 C, 2 x OMe), 73.79, 74.37, 74.95, and 75.69 (4 C, 4 x CHOR), 95.76 and 96.14 (2 C, 2 x O*C*H_2_O), 110.23 (1 C, *C*Me_2_), 206.11 (1 C,*C*=O); *m/z* (CI, NH_3_), 322 (M + NH_4_^+^), [Found: M + NH_4_^+^, 322.1858. C_14_H_28_O_7_N requires M + NH_4_^+^ 322.1866].

**Methoxymethyl 2,3-*O*-isopropylidene-5a-carba-β-d-rhamnopyranoside (54):** In a dry round bottom flask, (3*S*,4*R*,5*R*)-3,4-(isopropylidenedioxy)-5-(methoxymethoxy)-1-methylcyclohexene (**14**) (0.288 g, 1.0 mmol) was dissolved in dry THF (15 ml) and placed at 0 °C. Borane-methylsulphide complex (2 M in THF, 0.6 ml, 1.2 mmol) was added dropwise and the reaction mixture was stirred at room temperature for 2 h. After this time elapsed, water (1 ml) was added, followed by aqueous sodium hydroxide (2 M in water, 0.9 ml, 1.8 mmol) and hydrogen peroxide (27%, 4.8 mmol). During this time the flask was cooled in an ice-water bath. After stirring at room temperature overnight, a further amount of sodium hydroxide (2 M in water, 20 ml) was added with water (10 ml) and the reaction mixture was extracted with ethyl acetate (3 x 50 ml). The organic extracts were combined, dried over Na_2_SO_4_ and the solvent removed under reduced pressure to give the crude product which was purified by flash chromatography on silica gel with light petroleum/diethyl ether (3:1 to 1:4) to yield the title compound **54** (0.19 g, 77%) as a colourless oil, [α]_D_ −27.0 (*c* 1.65 in CHCl_3_); ν_max_(film)/cm^-1^ 3467 (OH), 2985, 2935, 2892, 1727, 1639, 1454, 1380, 1218, 1145, 1106, 1037, 917, 863 and 790; δ_H_ (270 MHz) 1.06 (3 H, d, *J* 5.94, CHC*H_3_*), 1.40 (3 H, s, Me), 1.56 (3 H, s, Me), 1.59–1.64 (1 H, d, *J* 12.53, CH*H*CHCH_3_), 1.77–1.84 (1 H, dt, *J* 3.3 and 12.54, C*H*HCHCH_3_), 2.58 (1 H, bs, C*H*CH_3_), 3.27–3.34 (1 H, dd, *J* 7.26, 7.91 and 10.56, 11.22, C*H*OH), 3.41 (3 H, s, OMe), 3.84–3.89 (2 H, dd, *J* 5.28 and 7.25, C*H*CHOH and C*H*CHOMOM), 4.41 (1 H, t, *J* 3.96, C*H*OMOM), 4.73 (1 H, d, *J* 7.25, OC*H*HO), 4.77 (1 H, d, *J* 7.26, OCH*H*O); δ_C_ (67.8 MHz) 26.30 (1 C, CH*C*H_3_), 28.37 (1 C, Me), 29.62 (1 C, Me), 32.81 (1 C, *C*HCH_3_), 32.98 (1 C, CH_2_), 55.43 (1 C, OMe), 72.93, 76.13 and 77.93 (3 C, 3 x CHOR), 82.01 (1 C, CHOH), 95.39 (1 C, OCH_2_O), 109.85 (1 C, *C*Me_2_); *m/z* (EI) 247 (MH^+^) and 215. [Found: MH^+^ 247.1545. C_12_H_23_O_5_ requires 247.1545].

**5a-Carba-β-d-rhamnopyranose (55):** Methoxymethyl 2,3-*O*-isopropylidene-5a-carba-β-d-rhamnopyranoside (**54**) (0.048 g, 0.194 mmol) was dissolved in methanol (2 ml) and a solution of HCl (6 N in water, 1 ml) was added at room temperature. After 12 h, the solvents were evaporated and the crude crystals of **55** (0.034 g, 99%) obtained were directly analysed, [α]_D_ +7.0 (*c* 1.1 in CH_3_OH); mp 166–167 °C; δ_H_ (270 MHz, CD_3_OD) 1.04 (3 H, d, *J* 6.6, Me), 1.37 (1 H, bm, C*H*Me), 1.59 (2 H, m, *J* 3.3 and 10.56, C*H_2_*), 3.26–3.36 (2 H, bm, *J* 10.55, C*H*OHCH_2_ and C*H*OH), 3.64 (1 H, bm, C*H*OH), 3.98 (1 H, bs, C*H*OH); δ_C_ (67.8 MHz) 18.36 (1 C, Me), 35.26 (1 C, CH_2_), 36.38 (1 C, *C*HMe), 70.76, 75.03, 76.16 and 76.39 (4 C, 4 x CHOH); *m/z* (CI, NH_3_) 180 (M + NH_4_^+^), 162 (M^+^). [Found: M + NH_4_^+^ 180.1238. C_7_H_18_O_4_N requires 180.1236].

**2,3,4,5-Tetra-*O*-acetyl-5a-carba-β-d-rhamnopyranose (56):** 5a-Carba-β-d-rhamnopyranose (**55**) (0.034 g, 0.21 mmol) was dissolved in dry pyridine (1 ml). Acetic anhydride (1 ml) was added at room temperature. After 12 h, the solvents were evaporated and the crude product **56** was purified by flash chromatography on silica gel with light petroleum/diethyl ether (1:1) as eluent to yield the title compound **56** (0.049 g, 99%) as a colourless oil, [α]_D_ +13 (*c* 1.65 in CHCl_3_); δ_H_ (400.1 MHz) (with the help of 400 COSY) 0.99 (3 H, d, *J* 6.05, Me), 1.70–1.75 (1 H, t, *J* 12, C*Hβ*Hα), 1.73 (1 H, m, *J*3.5, C*H*Me), 1.83–1.88 (1 H, m, CHβ*Hα*), 1.98*,* 2.00, 2.05 and 2.17 (12 H, 4s, 4 x OC*Me*), 4.92 (1 H, dd, *J* 2.8 and 10.2, C*H*(OAc)CH(OAc)CHMe), 4.94 (1 H, ddd, *J* 2.7, 4.8 and 11.66, C*H*OAcCH_2_), 5.02 (1 H, t, *J* 10.1, C*H*OAcCHMe), 5.55 (1 H, dt, *J* 1.4, 2.73, C*H*OAcCHOAcCH_2_); δ_C_ (100.6 MHz) (with the help of DEPT and a 400.1 ^1^H-^13^C NMR) 16.4 (1 C, Me), 19.62, 19.78, 19.83 and 19.89 (4 C, 4 x OC*Me*), 30.97 (1 C, *C*HMe), 31.32 (1 C, *C*H_2_), 67.71 (1 C, *C*HOAcCH_2_), 69.04 (1 C, *C*HOAcCHOAcCH_2_), 70.73 (1 C, *C*HOAcCHOAcCHMe), 72.54 (1 C, *C*HOACCHMe), 168.87, 168.8, 169.14 and 169.47 (4 C, 4 x C=O); *m/z* (CI, NH_3_) 348 (M + NH_4_^+^), 230, 153 and 77. [Found: M + NH_4_^+^ 348.1658. C_15_H_26_O_8_N requires 348.1658].
